# Supplementary material for: Ultra-photostable small-molecule dyes facilitate near-infrared biophotonics
Source: Nat Commun. 2024 Mar 22;15:2593. doi: 10.1038/s41467-024-46853-0 (PMC10960032; doi:10.1038/s41467-024-46853-0)
Supplement: Supplementary file 1 — Supplementary Information [file 41467_2024_46853_MOESM1_ESM.pdf]

Supplementary Information

Ultra-photostable Small-molecule Dyes Facilitate Near-infrared Biophotonics

Kui Yan,[1,+] Zhubin Hu,[2,+] Peng Yu,[1] Zuyang He,[1] Ying Chen,[1] Jiajian Chen,[3] Haitao Sun,[2,*] Shangfeng Wang,[1,*] and Fan Zhang[1,*]

[1] Department of Chemistry, State Key Laboratory of Molecular Engineering of Polymers, Shanghai Key Laboratory of Molecular Catalysis and Innovative Materials and iChem, Fudan University, Shanghai 200433, P.R. China;

[2] State Key Laboratory of Precision Spectroscopy, School of Physics and Electronic Science, East China Normal University, Shanghai 200241, P.R. China;

[3] Department of Breast Surgery, Key Laboratory of Breast Cancer in Shanghai, Fudan University Shanghai Cancer Center, Shanghai 200032, P. R. China.

Contents

Supplementary Methods..3

Supplementary Tables 17

Supplementary Figures..20

Supplementary Notes 77

Supplementary References..78

Supplementary Methods

Materials and reagents

Analytical grade solvents including petroleum ether, dichloromethane, and ethyl acetate were purchased from Titan Scientific. Dulbecco's modified eagle medium (DMEM) cell culture was purchased from Gibco. Fetal bovine serum (FBS) was purchased from ExCell. Trypsin (0.25%) and penicillin-streptomycin solution (100X) were purchased from MesGen Biotechnology. All other chemicals were purchased from major manufacturers in China including TCI, Macklin and J&K. All chemicals were used as received without further purification unless otherwise noted.

Characterization

^{1}H-NMR and ^{13}C-NMR spectra were obtained on a Bruker AV-400 spectrometer. Chemicals shifts are referenced to the residue solvent peaks and given in ppm. MALDI-TOF MS analyses were performed in AB SCIEX 4800 Plus MALDITOF/TOF™ mass spectrometer. LC/MS analyses were conducted in LC-20AD linked AB SCIEX Triple TOF 4600. Surface molecular charge, partition coefficient (logD) and hydrophobicity were calculated using MarvinSketch (ChemAxon). Absorbance spectra were acquired with a PerkinElmer Lambda 750S UV-visible-NIR spectrometer. Fluorescence spectra were recorded on Horiba Fluorescence Spectrometer instrument (Fluorolog™ HORIBA Scientific) with external 808 nm (MDL-H-5W) and 980 nm (MDL-H-5W) semiconductor lasers (Changchun New Industries Optoelectronics Tech. Co., Ltd.) as excitation sources. Quartz cuvettes (1 cm) were used for absorbance and emission measurements. NIR-II cellular intravital imaging was conducted on an epifluorescence microscopy system with 640×512 pixel 2D InGaAs NIRvava camera. In vivo NIR-II fluorescence images were captured by a modified home-built InGaAs NIRvana 640 CCD camera (Princeton Instruments Inc.). Photoacoustic signals and images were captured by VevoLAZR (FujiFilm VisualSonics Inc.) system with Nd: YAG laser with optical parametric oscillator (OPO) (680~970 nm) as excitation source. Femtosecond and nanosecond transient absorption (TA) measurements were carried out on commercial transient absorption spectrometers (Helios Fire Transient absorption spectrometer and Helios-EOS fire, respectively, ultrafast system). X-ray crystallography data sets were collected with Bruker D8 VENTURE X-ray diffractometer at 173(2) K, the structure was solved by direct

methods using SHELXS and refined against F2 on all data by full-matrix least-squares with SHELXL-2014 following established refinement strategies.

Measurement of fluorescence quantum yield (QY)

Quantum yields (Φ_{fl}) were determined in various solvents relative to IR26 (Φ_{fl} = 0.05% in DCE),[1] from plots of integrated fluorescence intensity vs. absorbance, according to the following relationship:

$$\Phi_{fl.s} = \Phi_{fl.r} \times \frac{n_s^2}{n_r^2} \times \frac{K_s}{K_r} \quad (1)$$

where subscripts r and s denote standard and test sample, respectively, Φ_{fl} is the fluorescence quantum yield, K is the slop of the integrated fluorescence intensity vs. absorbance plot, and n is the refractive index of the solvent. Measurements were performed with the absorbance at 808 nm of all dye solutions <0.1 in order to maximize illumination homogeneity and optical transparency. The 808 nm laser was used as the excitation source and the emission spectrum in the 850-1500 nm region was acquired in fluorescence spectrometer.

Preparation of various bioactive species

GSH: A 20 mM stock solution of GSH was prepared in deionized water.

H_2O_2: A commercial H_2O_2 (30% in water) was diluted before use. The concentration of H_2O_2 stock was determined by measuring the absorbance at 240 nm with molar extinction coefficient of 43.6 M^{-1} cm^{-1}.

$\cdot O_2^-$: A 10 mM stock solution of KO_2 was dissolved in DMSO directly.

·OH: ·OH was prepared by the Fenton reaction between $FeCl_2$ stock (20 mM) and H_2O_2 stock (20 mM).

ClO^-: ClO^- stock solution (20 mM in deionized water, pH=12) was prepared by dilution of commercial NaClO solution and assayed by measuring the absorbance at 293 nm ($\varepsilon_{293\ nm}$ = 350 M^{-1} cm^{-1}).

$ONOO^-$: Peroxynitrite stock (10 mM in 0.01 M NaOH) was obtained following the literature procedure[2] and the concentration was determined by measuring the absorbance at 302 nm ($\varepsilon_{302\ nm}$ =1670 M^{-1} cm^{-1}).

Cell viability

Mouse breast cancer cells (4T1, catalog no.: TCM32) were provided by Cell Bank, Chinese Academy of Science. All cells were cultured in Dulbecco's Modified Eagle medium (DMEM) supplemented with 10% FBS and 1% Penicillin-Streptomycin at 37 °C in a humidified atmosphere of 5% CO_2. 4T1 cells were cultured in a 96-well plate (8×10^3 cells/well) after 24 h incubation, the medium was replaced with 100 μL of fresh DMEM containing AF2 and AF3 with concentrations of 0, 0.2, 0.5, 1.0, 2.0, 5.0, 10 and 20 μM, respectively. Cells were incubated further for 4 h. To detect the cytotoxicity, 100 μL Cell Counting Kit-8 (CCK-8) solution (CCK-8: DMEM= 1: 9) was added to each well of the microliter plate and the plate was incubated in the CO_2 incubator for additional 4 h. Enzyme dehydrogenase in living cells was oxidized by this kit to orange carapace. The quality was assessed calorimetrically by using a multi-reader (TECAN, Infinite M200, Germany). The measurements were based on the absorbance values at 450 nm. Following formula was used to calculate the viability of cell growth:

$$V = \frac{Abs_t - Abs_b}{Abs_c - Abs_b} \times 100\% \tag{2}$$

Where V is cell viability, subscripts t, b and c represent treatment group, blank group and control group, respectively.

Synthetic procedures

1) General procedure for preparation of compound 1 via C-N cross-coupling

The following procedure for **1c** is representative. A vial was charged with 3,6-dibromo-9-fluorenone (338 mg, 1 mmol), $Pd_2(dba)_3$ (22.8 mg, 25 μmol, 0.025 eq), and tBuONa (576 mg, 6 mmol, 6 eq). The vial was sealed and vacuated/backfilled with nitrogen (3×). Anhydrous toluene (10 mL) was added, followed by addition of $PtBu_3$ (15 mg, 75 μmol, 10% wt in toluene, 0.075eq) and dimethylamine (2 mL, 4 mmol, 2 M in THF, 4 eq). The reaction was then stirred and refluxed at 100 °C for 4 h. It was subsequently cooled to room temperature, diluted with DCM, deposited onto Celite, and concentrated to dryness. The crude mixture was dispersed with ethyl acetate (3 mL), filtrated to afford the title compound as an orange solid (228 mg, 86%).

3,6-bis(*tert*-butyl carbamate)-9H-fluoren-9-one (1a): 3,6-dibromo-9-fluorenone (338 mg, 1 mmol), $Pd_2(dba)_3$ (22.8 mg, 25 μmol, 0.025 eq), tBuONa (576 mg, 6 mmol, 6 eq), $PtBu_3$ (15 mg, 75 μmol, 10% wt in

toluene, 0.075eq) and tert-butyl carbamate (468 mg, 4 mmol, 4 eq) were used, purified with silica gel chromatography (PE/EA, 3/1, v/v), affording **1a** as a yellow solid (295 mg, 72%). ^{1}H NMR (400 MHz, $CDCl_3$) δ 7.72 (d, *J* = 1.5 Hz, 2H), 7.53 (d, *J* = 8.1 Hz, 2H), 7.11 (dd, *J* = 8.1, 1.9 Hz, 2H), 6.82 (s, 2H), 1.54 (s, 18H); ^{13}C NMR (101 MHz, $CDCl_3$) δ 152.24 (C), 145.62 (C), 144.42 (C), 129.77 (C), 125.23 (C), 117.75 (C), 110.31 (C), 28.42 (CH3).

3,6-bis(*tert*-butyl methylcarbamate)-9H-fluoren-9-one (1b): 3,6-dibromo-9-fluorenone (338 mg, 1 mmol), $Pd_2(dba)_3$ (22.8 mg, 25 μmol, 0.025 eq), tBuONa (576 mg, 6 mmol, 6 eq), $PtBu_3$ (15 mg, 75 μmol, 10% wt in toluene, 0.075eq) and tert-butyl methylcarbamate (524 mg, 4 mmol, 4 eq) were used, purified with silica gel chromatography (PE/EA, 3/1, v/v), affording **1b** as a yellow solid (276 mg, 63%). ^{1}H NMR (400 MHz, $CDCl_3$) δ 7.60 (d, *J* = 8.0 Hz, 2H), 7.47 (d, *J* = 1.7 Hz, 2H), 7.15 (dd, *J* = 8.0, 1.9 Hz, 2H), 3.32 (s, 6H), 1.50 (s, 18H); ^{13}C NMR (101 MHz, $CDCl_3$) δ 154.23 (C), 149.79 (C), 144.66 (C), 131.24 (C), 124.97 (C), 124.64 (C), 117.18 (C), 81.42 (C), 37.20 (CH3), 28.46 (CH3).

3,6-bis(dimethylamino)-9H-fluoren-9-one (1c): (86%, orange solid) ^{1}H NMR (400 MHz, $CDCl_3$) δ 7.48 (d, *J* = 8.4 Hz, 2H), 6.75 (d, *J* = 2.2 Hz, 2H), 6.43 (dd, *J* = 8.4, 2.3 Hz, 2H), 3.09 (s, 12H); ^{13}C NMR (101 MHz, $CDCl_3$) δ 191.76 (C), 154.59 (C), 146.19 (C), 125.34 (C), 124.47 (C), 110.38 (C), 103.19 (C), 40.72 (CH3).

3,6-bis(diethylamino)-9H-fluoren-9-one (1d): 3,6-dibromo-9-fluorenone (338 mg, 1 mmol), $Pd_2(dba)_3$ (22.8 mg, 25 μmol, 0.025 eq), tBuONa (576 mg, 6 mmol, 6 eq), $PtBu_3$ (15 mg, 75 μmol, 10% wt in toluene, 0.075eq) and diethylamine (292 mg, 4 mmol, 4 eq) were used, affording **1d** as an orange solid (170 mg, 53%). ^{1}H NMR (400 MHz, $CDCl_3$) δ 7.48 (d, *J* = 8.4 Hz, 2H), 6.72 (d, *J* = 2.3 Hz, 2H), 6.43 (dd, *J* = 8.5, 2.3 Hz, 2H), 3.47 (q, *J* = 7.1 Hz, 8H), 1.24 (t, *J* = 7.1 Hz, 12H); ^{13}C NMR (101 MHz, $CDCl_3$) δ 191.38 (C), 152.27 (C), 146.46 (C), 125.63 (C), 123.92 (C), 109.90 (C), 102.55 (C), 44.98 (CH2), 13.01 (CH3).

3,6-di(azetidin-1-yl)-9H-fluoren-9-one (1e): 3,6-dibromo-9-fluorenone (338 mg, 1 mmol), $Pd_2(dba)_3$ (22.8 mg, 25 μmol, 0.025 eq), tBuONa (576 mg, 6 mmol, 6 eq), $PtBu_3$ (15 mg, 75 μmol, 10% wt in toluene, 0.075eq) and azetidine (228 mg, 4 mmol, 4 eq) were used, affording **1e** as an orange solid (180 mg, 62%). ^{1}H NMR (400 MHz, $CDCl_3$) δ 7.45 (d, *J* = 8.0 Hz, 2H), 6.39 (s, 2H), 6.11 (d, *J* = 8.1 Hz, 2H), 4.01 (t, *J* = 7.1 Hz, 9H), 2.42 (p, *J* = 7.0 Hz, 5H); ^{13}C NMR (101 MHz, $CDCl_3$) δ 155.43 (C), 145.78 (C), 125.21 (C), 124.89 (C), 109.04 (C), 102.06 (C), 51.81 (CH2), 16.67 (CH2).

3,6-bis(3,3-difluoroazetidin-1-yl)-9H-fluoren-9-one (1f): 3,6-dibromo-9-fluorenone (338 mg, 1 mmol), $Pd_2(dba)_3$ (22.8 mg, 25 μmol, 0.025 eq), tBuONa (576 mg, 6 mmol, 6 eq), $PtBu_3$ (15 mg, 75 μmol, 10% wt in toluene, 0.075eq) and 3,3-difluoroazetidine (372 mg, 4 mmol, 4 eq) were used, affording **1f** as an orange solid

(235 mg, 65%). ^{1}H NMR (400 MHz, $CDCl_3$) δ 7.50 (d, J = 8.1 Hz, 2H), 6.46 (d, J = 1.9 Hz, 2H), 6.23 (dd, J = 8.1, 2.0 Hz, 2H), 4.35 (t, J = 11.6 Hz, 8H); ^{13}C NMR (101 MHz, $CDCl_3$) δ 191.34 (C), 153.52 (C), 145.73 (C), 126.51 (C), 125.63 (C), 115.68 (C), 111.08 (C), 103.63 (C), 63.23 (CH2).

3,6-di(pyrrolidin-1-yl)-9H-fluoren-9-one (1g): 3,6-dibromo-9-fluorenone (338 mg, 1 mmol), $Pd_2(dba)_3$ (22.8 mg, 25 μmol, 0.025 eq), tBuONa (576 mg, 6 mmol, 6 eq), $PtBu_3$ (15 mg, 75 μmol, 10% wt in toluene, 0.075eq) and pyrrolidine (284 mg, 4 mmol, 4 eq) were used, affording **1g** as an orange solid (267 mg, 84%). ^{1}H NMR (400 MHz, $CDCl_3$) δ 7.48 (d, *J* = 8.3 Hz, 2H), 6.63 (d, *J* = 2.1 Hz, 2H), 6.30 (dd, *J* = 8.3, 2.2 Hz, 2H), 3.48 – 3.36 (m, 8H), 2.12 – 1.98 (m, 8H); ^{13}C NMR (101 MHz, $CDCl_3$) δ 191.67 (C), 151.93 (C), 146.10 (C), 125.26 (C), 123.93 (C), 110.14 (C), 103.21 (C), 48.02 (CH2), 25.54 (CH2).

3,6-di(piperidin-1-yl)-9H-fluoren-9-one (1h): 3,6-dibromo-9-fluorenone (338 mg, 1 mmol), $Pd_2(dba)_3$ (22.8 mg, 25 μmol, 0.025 eq), tBuONa (576 mg, 6 mmol, 6 eq), $PtBu_3$ (15 mg, 75 μmol, 10% wt in toluene, 0.075eq) and piperidine (340 mg, 4 mmol, 4 eq) were used, affording **1h** as an orange solid (252 mg, 73%). ^{1}H NMR (400 MHz, $CDCl_3$) δ 7.49 (d, *J* = 8.4 Hz, 2H), 6.97 (s, 2H), 6.65 (dd, *J* = 8.4, 1.8 Hz, 2H), 3.45 – 3.33 (m, 8H), 1.75 – 1.61 (m, 12H); ^{13}C NMR (101 MHz, $CDCl_3$) δ 191.44 (C), 155.81 (C), 146.03 (C), 125.67 (C), 125.39 (C), 113.16 (C), 106.06 (C), 49.32 (CH2), 25.64 (CH2), 24.49 (CH2).

3,6-di(azepan-1-yl)-9H-fluoren-9-one (1i): 3,6-dibromo-9-fluorenone (338 mg, 1 mmol), $Pd_2(dba)_3$ (22.8 mg, 25 μmol, 0.025 eq), tBuONa (576 mg, 6 mmol, 6 eq), $PtBu_3$ (15 mg, 75 μmol, 10% wt in toluene, 0.075eq) and azepane (396 mg, 4 mmol, 4 eq) were used, affording **1i** as an orange solid (306 mg, 82%). ^{1}H NMR (400 MHz, $CDCl_3$) δ 7.47 (d, *J* = 8.4 Hz, 2H), 6.75 (d, *J* = 2.2 Hz, 2H), 6.45 (dd, *J* = 8.5, 2.3 Hz, 2H), 3.64 – 3.50 (m, 8H), 1.89 – 1.77 (m, 8H), 1.62 – 1.52 (m, 8H); ^{13}C NMR (101 MHz, $CDCl_3$) δ 153.38 (C), 146.35 (C), 125.52 (C), 123.97 (C), 109.74 (C), 102.43 (C), 49.90 (CH2), 27.73 (CH2), 27.02 (CH2).

3,6-bis(methyl(phenyl)amino)-9H-fluoren-9-one (1j): 3,6-dibromo-9-fluorenone (338 mg, 1 mmol), $Pd_2(dba)_3$ (22.8 mg, 25 μmol, 0.025 eq), tBuONa (576 mg, 6 mmol, 6 eq), $PtBu_3$ (15 mg, 75 μmol, 10% wt in toluene, 0.075eq) and N-methylaniline (428 mg, 4 mmol, 4 eq) were used, affording **1j** as an orange solid (273 mg, 70%). ^{1}H NMR (400 MHz, $CDCl_3$) δ 7.46 (d, *J* = 8.3 Hz, 2H), 7.43 – 7.37 (m, 4H), 7.25 – 7.18 (m, 6H), 6.83 (d, *J* = 2.1 Hz, 2H), 6.57 (dd, *J* = 8.3, 2.1 Hz, 2H), 3.40 (s, 6H); ^{13}C NMR (101 MHz, $CDCl_3$) δ 191.47(C), 154.00(C), 147.78(C), 145.82(C), 130.02(C), 126.36(C), 125.87(C), 125.45(C), 125.26(C), 114.40(C), 106.80(C), 40.96(CH3).

3,6-bis(diphenylamino)-9H-fluoren-9-one (1k): 3,6-dibromo-9-fluorenone (338 mg, 1 mmol), $Pd_2(dba)_3$ (22.8 mg, 25 μmol, 0.025 eq), tBuONa (576 mg, 6 mmol, 6 eq), $PtBu_3$ (15 mg, 75 μmol, 10% wt in toluene,

0.075eq) and diphenylamine (676 mg, 4 mmol, 4 eq) were used, affording **1k** as an orange solid (380 mg, 74%). ^{1}H NMR (400 MHz, $CDCl_3$) δ 7.48 (d, *J* = 8.2 Hz, 2H), 7.33 – 7.27 (m, 8H), 7.16 – 7.07 (m, 12H), 7.01 (d, *J* = 1.9 Hz, 2H), 6.77 (dd, *J* = 8.2, 2.0 Hz, 2H); ^{13}C NMR (101 MHz, $CDCl_3$) δ 191.01 (C), 153.53 (C), 146.82 (C), 145.34 (C), 129.70 (C), 128.60 (C), 125.71 (C), 125.27 (C), 124.52 (C), 121.40 (C), 113.45 (C).

3,6-di(indolin-1-yl)-9H-fluoren-9-one (1m): 3,6-dibromo-9-fluorenone (338 mg, 1 mmol), $Pd_2(dba)_3$ (22.8 mg, 25 μmol, 0.025 eq), tBuONa (576 mg, 6 mmol, 6 eq), $PtBu_3$ (15 mg, 75 μmol, 10% wt in toluene, 0.075eq) and indoline (476 mg, 4 mmol, 4 eq) were used, affording **1m** as a dark red solid (331 mg, 80%). ^{1}H NMR (400 MHz, $CDCl_3$) δ 7.63 (d, *J* = 8.2 Hz, 2H), 7.34 (d, *J* = 2.0 Hz, 2H), 7.31 (d, *J* = 8.0 Hz, 2H), 7.24 (d, *J* = 7.3 Hz, 2H), 7.17 (t, *J* = 7.7 Hz, 2H), 7.04 (dd, *J* = 8.2, 2.0 Hz, 2H), 6.87 (t, *J* = 7.4 Hz, 2H), 4.10 (t, *J* = 8.3 Hz, 4H), 3.19 (t, *J* = 8.3 Hz, 4H); ^{13}C NMR (101 MHz, $CDCl_3$) δ 149.14 (C), 145.68 (C), 145.35 (C), 132.37 (C), 127.57 (C), 127.42 (C), 125.57 (C), 125.54 (C), 120.73 (C), 115.52 (C), 110.33 (C), 107.83 (C), 52.46 (CH2), 28.28 (CH2).

2) General procedure for preparation of AF dyes via nucleophilic addition of aryl lithium reagents

Method 1: The following procedure for AF1 is representative. A vial was charged with 2-bromo-1,3-dimethylbenzene (277.5 mg, 1.5 mmol, 1 eq), sealed, and vacuated/backfilled with nitrogen (3×). After dissolving the bromide in anhydrous THF (2 mL) and cooling the reaction to −78 °C, *n*-butyllithium (0.825 mL, 2 M in cyclohexane, 1.1 eq) was slowly added. The reaction was then stirred in the same temperature for further 5 min. A solution of 3,6-bis(*tert*-butyl carbamate)-9H-fluoren-9-one (**1a**; 205 mg, 0.5 mmol) in anhydrous THF (7 mL) was then added dropwise. The reaction mixture was warmed to room temperature and stirred for 30 min. It was subsequently quenched with water, extracted with EtOAc (2×). The combined organics were dried (Na_2SO_4), filtered, evaporated, and purified with silica gel chromatography (0–50% EtOAc/PE, linear gradient). The alcohol intermediate was then redissolved in CH_2Cl_2 (5 mL), and trifluoroacetic acid (1 mL) was added. The reaction was stirred at room temperature overnight. Toluene (3 mL) was added; the reaction mixture was concentrated to dryness and then triturated with Et_2O/PE (v/v, 1/4) to provide AF1 as a green solid (103 mg, 50% total, TFA salt). Analytical HPLC and NMR indicated that the material was >95% pure and did not require further purification.

AF1 (TFA salt): (50%, green solid) ^{1}H NMR (400 MHz, $CDCl_3$) δ 8.68 (s, 4H), 7.33 – 7.18 (m, 3H), 6.95 (s, 2H), 6.59 (d, J = 8.4 Hz, 2H), 6.18 (d, J = 8.4 Hz, 2H), 2.16 (s, 6H); ^{13}C NMR (101 MHz, $CDCl_3$) δ 172.07 (C), 160.87 (C), 147.57 (C), 135.41 (C), 134.64 (C), 131.10 (C), 129.86 (C), 128.87 (C), 128.37 (C), 114.44

(C), 114.15 (C), 20.31 (CH3); Maldi-Tof/Tof-MS calcd for $C_{21}H_{19}N_2$ $[M]^+$ 299.1543, found 299.1694.

AF2 (TFA salt): Method 1 was applied. 2-bromo-1,3-dimethylbenzene (277.5 mg, 1.5 mmol, 1 eq), *n*-butyllithium (0.825 mL, 2 M in cyclohexane, 1.1 eq), 3,6-bis(*tert*-butyl methylcarbamate)-9H-fluoren-9-one (**1b**; 219 mg, 0.5 mmol) were used, affording AF2 as a green solid (163 mg, 74% total, TFA salt). ^{1}H NMR (400 MHz, $CDCl_3$) δ 9.11 (s, 2H), 7.26 – 7.21 (m, 1H), 7.19 (d, *J* = 1.2 Hz, 2H), 7.11 (d, *J* = 7.6 Hz, 2H), 6.61 (d, *J* = 8.7 Hz, 2H), 6.06 (dd, *J* = 8.7, 1.3 Hz, 2H), 3.09 (s, 6H), 2.17 (s, 6H); ^{13}C NMR (101 MHz, $CDCl_3$) δ 171.59(C), 159.38(C), 146.59(C), 135.65(C), 133.93(C), 131.10(C), 129.95(C), 129.32(C), 127.88(C), 114.50(C), 109.90(C), 30.98(CH3), 20.29(CH3); Maldi-Tof/Tof-MS calcd for $C_{23}H_{23}N_2$ $[M]^+$ 327.1856, found 327.1960.

Method 2: The following procedure for AF3 is representative. A vial was charged with 2-bromo-1,3-dimethylbenzene (277.5 mg, 1.5 mmol, 1 eq), sealed, and vacuated/backfilled with nitrogen (3×). After dissolving the bromide in anhydrous THF (2 mL) and cooling the reaction to −78 °C, *n*-butyllithium (0.825 mL, 2 M in cyclohexane, 1.1 eq) was slowly added. The reaction was then stirred in the same temperature for further 5 min. A solution of 3,6-bis(dimethylamino)-9H-fluoren-9-one (**1c**; 133 mg, 0.5 mmol) in anhydrous THF (7 mL) was then added dropwise. The reaction mixture was warmed to room temperature and stirred for 30 min. It was subsequently quenched with diluted perchloric acid (10 wt%, 1 mL), stirred for another 30 min until product precipitated from solution (147 mg, 65%). Analytical HPLC and NMR indicated that the material was >95% pure and did not require further purification.

AF3 (perchlorate salt): (65%, dark green solid) ^{1}H NMR (400 MHz, $CDCl_3$) δ 7.67 (d, *J* = 2.1 Hz, 2H), 7.24 (d, *J* = 7.5 Hz, 1H), 7.13 (d, *J* = 7.6 Hz, 2H), 6.58 (d, *J* = 8.9 Hz, 2H), 6.08 (dd, *J* = 8.9, 2.1 Hz, 2H), 3.44 (s, 12H), 2.22 (s, 6H); ^{13}C NMR (101 MHz, $CDCl_3$) δ 158.36(C), 147.72(C), 135.39(C), 132.95(C), 129.20(C), 127.71(C), 113.62(C), 111.21(C), 42.17(CH3), 20.09(CH3); Maldi-Tof/Tof-MS calcd for $C_{25}H_{27}N_2$ $[M]^+$ 355.2169, found 355.2269.

AF4 (perchlorate salt): Method 2 was applied. 2-bromo-1,3-dimethoxybenzene (325 mg, 1.5 mmol, 1 eq), *n*-butyllithium (0.825 mL, 2 M in cyclohexane, 1.1 eq) and 3,6-bis(dimethylamino)-9H-fluoren-9-one (**1c**; 133 mg, 0.5 mmol) were used, affording AF4 as a brown solid (216 mg, 89%, perchlorate salt). ^{1}H NMR (400 MHz, DMSO) δ 7.49 (t, *J* = 8.4 Hz, 1H), 7.44 (d, *J* = 2.1 Hz, 2H), 6.84 (d, *J* = 8.5 Hz, 2H), 6.78 (d, *J* = 9.0 Hz, 2H), 6.33 (dd, *J* = 9.0, 2.1 Hz, 2H), 3.73 (s, 6H), 3.34 (s, 12H); ^{13}C NMR (101 MHz, DMSO) δ 158.53(C), 157.76(C), 147.30(C), 133.99(C), 133.58(C), 129.92(C), 112.23(C), 112.14(C), 105.49(C), 56.67(CH3),

42.06(CH3); Maldi-Tof/Tof-MS calcd for $C_{25}H_{27}N_2O_2$ $[M]^+$ 387.2067, found 387.2234.

AF5 (perchlorate salt): Method 2 was applied. 2-bromo-1,3-dimethylbenzene (277.5 mg, 1.5 mmol, 1 eq), *n*-butyllithium (0.825 mL, 2 M in cyclohexane, 1.1 eq) and 3,6-bis(diethylamino)-9H-fluoren-9-one (**1d**; 161 mg, 0.5 mmol) were used, affording AF5 as a brown solid (217 mg, 85%, perchlorate salt). ^{1}H NMR (400 MHz, $CDCl_3$) δ 7.65 (s, 2H), 7.27 – 7.22 (m, 1H), 7.13 (d, *J* = 7.7 Hz, 2H), 6.58 (d, *J* = 8.9 Hz, 2H), 6.09 (s, 2H), 3.75 (d, *J* = 6.4 Hz, 8H), 2.23 (s, 6H), 1.37 (t, *J* = 7.1 Hz, 12H); ^{13}C NMR (101 MHz, $CDCl_3$) δ 156.73(C), 148.18(C), 135.66(C), 133.13(C), 131.20(C), 129.32(C), 127.88(C), 113.40(C), 110.92(C), 47.19(CH2), 20.33(CH3), 13.97(CH3); Maldi-Tof/Tof-MS calcd for $C_{29}H_{35}N_2$ $[M]^+$ 411.2795, found 411.2924.

AF6 (perchlorate salt): Method 2 was applied. 2-bromo-1,3-dimethylbenzene (277.5 mg, 1.5 mmol, 1 eq), *n*-butyllithium (0.825 mL, 2 M in cyclohexane, 1.1 eq) and 3,6-di(azetidin-1-yl)-9H-fluoren-9-one (**1e**; 145 mg, 0.5 mmol) were used, affording AF6 as a dark green solid (220 mg, 92%, perchlorate salt). ^{1}H NMR (400 MHz, $CDCl_3$) δ 7.34 (s, 2H), 7.23 (d, *J* = 7.4 Hz, 1H), 7.12 (d, *J* = 7.6 Hz, 2H), 6.55 (d, *J* = 8.6 Hz, 2H), 5.78 (d, *J* = 8.6 Hz, 2H), 4.49 (t, *J* = 7.6 Hz, 8H), 2.60 – 2.47 (m, 4H), 2.19 (s, 6H); ^{13}C NMR (101 MHz, DMSO) δ 156.66(C), 146.28(C), 135.50(C), 132.48(C), 129.81(C), 128.91(C), 128.42(C), 111.43(C), 110.71(C), 53.46(CH2), 20.25(CH3), 16.22(CH2); Maldi-Tof/Tof-MS calcd for $C_{27}H_{27}N_2$ $[M]^+$ 379.2169, found 379.2245.

AF7 (perchlorate salt): Method 2 was applied. 2-bromo-1,3-dimethylbenzene (277.5 mg, 1.5 mmol, 1 eq), *n*-butyllithium (0.825 mL, 2 M in cyclohexane, 1.1 eq) and 3,6-bis(3,3-difluoroazetidin-1-yl)-9H-fluoren-9-one (**1f**; 181 mg, 0.5 mmol) were used, affording AF7 as a dark green solid (240 mg, 87%, perchlorate salt). ^{1}H NMR (400 MHz, $CDCl_3$) δ 7.36 (d, *J* = 2.1 Hz, 2H), 7.26 (t, *J* = 7.6 Hz, 1H), 7.13 (d, *J* = 7.6 Hz, 2H), 6.63 (d, *J* = 8.6 Hz, 2H), 5.82 (dd, *J* = 8.6, 2.1 Hz, 2H), 4.87 – 4.68 (m, 8H), 2.20 (s, 6H); ^{13}C NMR (101 MHz, $CDCl_3$) δ 178.35(C), 157.17(C), 148.14(C), 135.34(C), 134.53(C), 131.39(C), 130.43(C), 129.95(C), 128.14(C), 114.48(C), 113.34(C), 110.91(C), 64.40(CH2), 20.29(CH3); Maldi-Tof/Tof-MS calcd for $C_{27}H_{23}F_4N_2$ $[M]^+$ 451.1792, found 451.2016.

AF8 (perchlorate salt): Method 2 was applied. 2-bromo-1,3-dimethylbenzene (277.5 mg, 1.5 mmol, 1 eq), *n*-butyllithium (0.825 mL, 2 M in cyclohexane, 1.1 eq) and 3,6-di(pyrrolidin-1-yl)-9H-fluoren-9-one (**1g**; 159 mg, 0.5 mmol) were used, affording AF8 as a brown solid (215 mg, 85%, perchlorate salt). ^{1}H NMR (400 MHz, $CDCl_3$) δ 7.56 (d, *J* = 1.8 Hz, 2H), 7.24 (dd, *J* = 8.1, 7.1 Hz, 1H), 7.12 (d, *J* = 7.7 Hz, 2H), 6.55 (d, *J* = 8.8 Hz, 2H), 5.97 (dd, *J* = 8.8, 1.7 Hz, 2H), 3.81 (s, 8H), 2.21 (s, 6H), 2.09 (t, *J* = 6.6 Hz, 8H); ^{13}C NMR (101

MHz, $CDCl_3$) δ 172.57(C), 155.62(C), 147.49(C), 135.42(C), 132.66(C), 131.06(C), 129.52(C), 129.06(C), 127.65(C), 114.47(C), 111.73(C), 50.19(CH3), 25.14(CH2), 20.04(CH2); Maldi-Tof/Tof-MS calcd for $C_{29}H_{31}N_2$ $[M]^+$ 407.2482, found 407.2552.

AF9 (perchlorate salt): Method 2 was applied. 2-bromo-1,3-dimethylbenzene (277.5 mg, 1.5 mmol, 1 eq), *n*-butyllithium (0.825 mL, 2 M in cyclohexane, 1.1 eq) and 3,6-di(piperidin-1-yl)-9H-fluoren-9-one (**1h**; 173 mg, 0.5 mmol) were used, affording AF9 as a brown solid (216 mg, 81%, perchlorate salt). ^{1}H NMR (400 MHz, $CDCl_3$) δ 7.83 (s, 2H), 7.28 – 7.22 (m, 1H), 7.13 (d, *J* = 7.6 Hz, 2H), 6.62 (d, *J* = 8.9 Hz, 2H), 6.30 (s, 2H), 3.86 (s, 8H), 2.21 (s, 6H), 1.89 – 1.73 (m, 12H); ^{13}C NMR (101 MHz, $CDCl_3$) δ 157.23(C), 148.06(C), 135.71(C), 133.09(C), 131.27(C), 129.61(C), 129.31(C), 127.89(C), 113.74(C), 111.38(C), 50.53(CH2), 27.01(CH2), 24.50(CH2), 20.34(CH3); Maldi-Tof/Tof-MS calcd for $C_{31}H_{35}N_2$ $[M]^+$ 435.2795, found 435.3144.

AF10 (perchlorate salt): Method 2 was applied. 2-bromo-1,3-dimethylbenzene (277.5 mg, 1.5 mmol, 1 eq), *n*-butyllithium (0.825 mL, 2 M in cyclohexane, 1.1 eq) and 3,6-di(azepan-1-yl)-9H-fluoren-9-one (**1i**; 187 mg, 0.5 mmol) were used, affording AF10 as a brown solid (230 mg, 82%, perchlorate salt). ^{1}H NMR (400 MHz, DMSO) δ 7.63 (d, *J* = 2.2 Hz, 2H), 7.32 (dd, *J* = 8.3, 6.9 Hz, 1H), 7.23 (d, *J* = 7.7 Hz, 2H), 6.63 (d, *J* = 9.0 Hz, 2H), 6.43 (dd, *J* = 9.1, 2.2 Hz, 2H), 3.87 (t, *J* = 5.5 Hz, 8H), 2.17 (s, 6H), 1.80 (s, 9H), 1.55 (s, 8H); ^{13}C NMR (101 MHz, DMSO) δ 170.44(C), 157.22(C), 147.57(C), 135.51(C), 133.26(C), 131.25(C), 129.88(C), 129.15(C), 128.42(C), 113.09(C), 112.47(C), 52.24(CH3), 27.74(CH2), 26.21(CH2), 20.37(CH2); Maldi-Tof/Tof-MS calcd for $C_{33}H_{39}N_2$ $[M]^+$ 463.3108, found 463.3435.

AF11 (perchlorate salt): Method 2 was applied. 2-bromo-1,3-dimethoxybenzene (325 mg, 1.5 mmol, 1 eq), *n*-butyllithium (0.825 mL, 2 M in cyclohexane, 1.1 eq) and 3,6-di(azepan-1-yl)-9H-fluoren-9-one (**1i**; 187 mg, 0.5 mmol) were used, affording AF11 as a dark red solid (273 mg, 92%, perchlorate salt). ^{1}H NMR (400 MHz, $CDCl_3$) δ 7.43 (d, *J* = 2.2 Hz, 2H), 7.39 (t, *J* = 8.4 Hz, 1H), 6.72 (d, *J* = 9.0 Hz, 2H), 6.65 (d, *J* = 8.5 Hz, 2H), 6.10 (dd, *J* = 9.0, 2.2 Hz, 2H), 3.91 – 3.72 (m, 8H), 3.77 (s, 6H), 1.95 – 1.84 (m, 8H), 1.60 – 1.55 (m, 8H); ^{13}C NMR (101 MHz, $CDCl_3$) δ 166.42(C), 158.50(C), 157.17(C), 148.24(C), 134.13(C), 132.63(C), 130.34(C), 112.11(C), 110.58(C), 109.75(C), 104.53(C), 56.24(CH3), 52.21(CH2), 27.90(CH2), 26.61(CH2); Maldi-Tof/Tof-MS calcd for $C_{33}H_{39}N_2O_2$ $[M]^+$ 495.3006, found 495.3227.

AF12 (perchlorate salt): Method 2 was applied. 2-bromo-1,3-dimethylbenzene (277.5 mg, 1.5 mmol, 1 eq), *n*-butyllithium (0.825 mL, 2 M in cyclohexane, 1.1 eq) and 3,6-bis(methyl(phenyl)amino)-9H-fluoren-9-one (**1j**; 195 mg, 0.5 mmol) were used, affording AF12 as a brown solid (251 mg, 87%, perchlorate salt). ^{1}H NMR

(400 MHz, $CDCl_3$) δ 7.60 (d, *J* = 2.2 Hz, 2H), 7.50 (dd, *J* = 10.5, 4.8 Hz, 4H), 7.43 – 7.36 (m, 2H), 7.26 – 7.20 (m, 4H), 7.09 (d, *J* = 7.7 Hz, 2H), 6.98 (s, 1H), 6.42 (d, *J* = 8.9 Hz, 2H), 5.88 – 5.85 (m, 2H), 3.83 (s, 6H), 2.22 (s, 6H); ^{13}C NMR (101 MHz, $CDCl_3$) δ 175.57(C), 158.99(C), 148.63(C), 144.83(C), 135.45(C), 133.12(C), 131.07(C), 130.47(C), 129.62(C), 128.76(C), 128.23(C), 127.97(C), 126.73(C), 114.20(C), 114.10(C), 43.23(CH3), 20.35(CH3); Maldi-Tof/Tof-MS calcd for $C_{35}H_{31}N_2$ $[M]^+$ 479.2482, found 479.2677.

AF13 (perchlorate salt): Method 2 was applied. 2-bromo-1,3-dimethylbenzene (277.5 mg, 1.5 mmol, 1 eq), *n*-butyllithium (0.825 mL, 2 M in cyclohexane, 1.1 eq) and 3,6-bis(diphenylamino)-9H-fluoren-9-one (**1k**; 257 mg, 0.5 mmol) were used, affording AF13 as a purple solid (327 mg, 93%, perchlorate salt). ^{1}H NMR (400 MHz, $CDCl_3$) δ 7.45 (t, *J* = 7.7 Hz, 8H), 7.34 (t, *J* = 7.4 Hz, 4H), 7.26 – 7.22 (m, 9H), 7.13 (d, *J* = 7.6 Hz, 2H), 6.75 (d, *J* = 2.0 Hz, 2H), 6.63 (d, *J* = 8.8 Hz, 2H), 6.22 (dd, *J* = 8.8, 1.8 Hz, 2H), 2.28 (s, 6H); ^{13}C NMR (101 MHz, $CDCl_3$) δ 175.99(C), 158.30(C), 147.34(C), 143.64(C), 135.63(C), 134.39(C), 133.64(C), 130.47(C), 130.08(C), 128.70(C), 128.17(C), 127.62(C), 118.48(C), 116.80(C), 20.60(CH3); Maldi-Tof/Tof-MS calcd for $C_{45}H_{35}N_2$ $[M]^+$ 603.2795, found 603.3048.

AF14 (perchlorate salt): Method 2 was applied. 2-bromo-1,3-dimethylbenzene (277.5 mg, 1.5 mmol, 1 eq), *n*-butyllithium (0.825 mL, 2 M in cyclohexane, 1.1 eq) and 3,6-di(indolin-1-yl)-9H-fluoren-9-one (**1m**; 207 mg, 0.5 mmol) were used, affording AF14 as a purple solid (247 mg, 82%, perchlorate salt). ^{1}H NMR (400 MHz, $CDCl_3$) δ 7.87 (s, 2H), 7.43 (d, *J* = 8.1 Hz, 2H), 7.35 – 7.26 (m, 5H), 7.19 – 7.09 (m, 4H), 6.91 (d, *J* = 8.9 Hz, 2H), 6.71 (d, *J* = 8.8 Hz, 2H), 4.67 (t, *J* = 7.5 Hz, 4H), 3.30 (t, *J* = 7.4 Hz, 4H), 2.27 (s, 6H); ^{13}C NMR (101 MHz, $CDCl_3$) δ 172.23(C), 153.17(C), 147.80(C), 142.61(C), 136.93(C), 135.75(C), 132.87(C), 132.50(C), 129.66(C), 128.29(C), 128.07(C), 126.50(C), 125.93(C), 117.30(C), 116.73(C), 114.39(C), 54.74(CH3), 28.63(CH2), 20.49(CH2); Maldi-Tof/Tof-MS calcd for $C_{37}H_{31}N_2$ $[M]^+$ 503.2482, found 503.2802.

3) Procedure for preparation of AF derivatives

Preparation of AF2Ac: AF2 (33 mg, 0.075 mmol, 1 eq) was added into a round bottom flask, and dissolved with dried DCM (5 mL). Triethylamine (38 mg, 0.375 mmol, 5 eq) was then added and the solution was stirred rigorously. Then acetyl chloride (30 mg, 0.375 mol, 5 eq) was added and stirred for further 10 min. It was subsequently concentrated to dryness, washed with PE and EA (1:1, v/v, 10 mL totally) and filtrated (3 times) to afford AF2Ac as a yellow solid (19 mg, 69%). ^{1}H NMR (400 MHz, $CDCl_3$) δ 7.45 (s, 1H), 7.23 (d, J = 7.8 Hz, 1H), 7.15 (d, J = 7.5 Hz, 2H), 7.03 (dd, J = 7.8, 1.7 Hz, 1H), 6.86 (d, J = 7.9 Hz, 1H), 6.74 (s, 1H),

6.50 (d, J = 9.7 Hz, 1H), 5.88 (d, J = 9.1 Hz, 1H), 3.30 (s, 3H), 2.70 (s, 3H), 2.12 (d, J = 6.5 Hz, 6H), 1.94 (s, 3H). Maldi-Tof/Tof-MS calcd for $C_{25}H_{24}N_2O$ $[M+H]^+$ 369.1967, found 368.9492.

Preparation of AF2B: (4-(4,4,5,5-Tetramethyl-1,3,2-dioxaborolan-2-yl)phenyl)methanol (936 mg, 4 mmol) was dissolved with dry THF (30 mL) in a round bottom flask. The solution was stirred in ice bath, added with triethylamine (606 mg, 6 mmol) and triphosgen (594 mg, 2 mmol), and then further stirred for 4 h. The reaction mixture was filtered off through a celite pad, concentrated to dryness to afford chloridate intermediate as yellowish oil. AF2 (44 mg, 0.1 mmol, 1 eq) was dissolved with dry DCM (5 mL) in a round bottom flask. The solution was stirred in ice bath, then added with DIPEA (52 mg, 0.4 mmol, 4eq) and stirred for 5 min. A solution of chloridate intermediate (45 mg, 0.15 mmol, 1.5 eq) in 2 mL DCM was added into the reaction mixture and further stirred for 30 min. The reaction mixture was diluted with 30 mL DCM and washed with 0.1 M HCl, and concentrated to dryness. Et_2O (10 mL) was then added and the solution was filtered to collect the precipitate as crude product. The crude product was purified with HPLC ($MeOH/H_2O$, 0.1% TFA was added) to afford AF2B as a dark green solid (10 mg, 20%). ^1H NMR (400 MHz, $CDCl_3$) δ 8.03 – 7.75 (m, 2H), 7.47 – 7.29 (m, 3H), 7.21 – 7.06 (m, 2H), 6.91 (dd, J = 48.3, 8.6 Hz, 2H), 6.80 – 6.57 (m, 3H), 6.42 (d, J = 9.0 Hz, 1H), 5.24 (s, 2H), 3.47 (s, 3H), 3.29 (s, 3H), 2.17 (s, 6H). HRMS (ESI) calcd for $C_{31}H_{29}BN_2O_4$ $[M+H]^+$ 505.2299, found 505.2292.

Preparation of intermediate 2: A round bottom flask was dried, sealed and vacuated/backfilled with nitrogen (3×), then charged with 2,5-dibromo-1,3-dimethylbenzene (5.28 g, 20 mmol, 1 eq) and dry THF (60 mL). After cooling the solution to -78 °C, *n*-butyllithium (10 mL, 2 M in cyclohexane, 1 eq) was slowly added and the reaction was stirred vigorously for 2 h. A solution of bis(tert-butoxycarbonyl)oxide (4.37 g, 20 mmol, 1 eq) was dissolved in dry THF (15 mL), added into reaction mixture dropwise, and further stirred for 2 h. The reaction was quenched with water and extracted with DCM (2×). The combined organics were dried (Na_2SO_4), filtered, evaporated, and purified with silica gel chromatography (100% PE) to afford intermediate **2** as a colorless oil (1.94 g, 34%).

Preparation of AF3-COOH: Method 2 was applied to afford AF3-COOH *tert*-butyl ester as an intermediate. intermediate **2** (427 mg, 1.5 mmol, 1 eq), *n*-butyllithium (0.825 mL, 2 M in cyclohexane, 1.1 eq) and 3,6-bis(dimethylamino)-9H-fluoren-9-one (**1c**; 133 mg, 0.5 mmol) were used, affording AF3-COOH *tert*-butyl ester as a brown solid. Then the intermediate was dissolved with DCM/TFA (4:1, v/v, 10 mL totally) and stirred under room temperature for 12 h. Toluene (10 mL) was added and the reaction mixture was concentrated to dryness, further dried under vacuum pump overnight to afford AF3-COOH as a brown solid

(161 mg, 63%, TFA salt). ^1H NMR (400 MHz, DMSO) δ 7.79 (s, 2H), 7.62 (d, J = 1.7 Hz, 2H), 6.65 (d, J = 9.0 Hz, 2H), 6.37 (dd, J = 9.0, 1.7 Hz, 2H), 3.38 (s, 12H), 2.21 (s, 6H). ^{13}C NMR (101 MHz, DMSO) δ 169.03 (C), 167.70 (C), 158.07 (C), 146.97 (C), 136.31 (C), 135.88 (C), 132.79 (C), 131.96 (C), 129.14 (C), 128.78 (C), 113.62 (C), 113.04 (C), 42.31 (CH3), 20.20 (CH3). Maldi-Tof/Tof-MS calcd for $C_{26}H_{27}N_2O_2$ $[M]^+$ 399.2067, found 399.2165.

Preparation of AF2-COOH: Method 1 was applied. Intermediate **2** (427 mg, 1.5 mmol, 1 eq), *n*-butyllithium (0.825 mL, 2 M in cyclohexane, 1.1 eq), 3,6-bis(*tert*-butyl methylcarbamate)-9H-fluoren-9-one (**1b**; 219 mg, 0.5 mmol) were used, affording AF2-COOH as a green solid (121 mg, 50% total, TFA salt). ^1H NMR (400 MHz, DMSO) δ 9.29 (s, 2H), 7.78 (s, 2H), 7.20 (s, 2H), 6.65 (d, J = 7.8 Hz, 2H), 6.23 (d, J = 8.5 Hz, 2H), 3.07 (s, 6H), 2.22 (s, 6H); ^{13}C NMR (101 MHz, DMSO) δ 169.18 (C), 167.68 (C), 159.42 (C), 147.03 (C), 136.32 (C), 135.82 (C), 133.45 (C), 131.95 (C), 129.09 (C), 128.77 (C), 31.17 (CH3), 20.20 (CH3); Maldi-Tof/Tof-MS calcd for $C_{24}H_{23}N_2O_2$ $[M]^+$ 371.1754, found 371.1864.

Preparation of intermediate 3: 2-bromo-5-iodo-m-xylene (3.11 g, 10 mmol, 1 eq) was dissolved with diisopropylamine (50 mL) in a round bottom flask, stirred rigorously under room temperature. $[PdCl_2(PPh_3)_2]$ (210 mg, 0.3 mmol, 0.03 eq), CuI (114 mg, 0.6 mmol, 0.06 eq) and ethynyltrimethylsilane (1.18 g, 12 mmol, 1.2 eq) were then added and further stirred for 4 h. The reaction mixture was diluted with EA (200 mL) and washed with water (3×). The organic phase was dried (Na_2SO_4), filtered, evaporated, and purified with silica gel chromatography (100% PE) to afford intermediate **3** as a yellow oil (2.67 g, 95%).

Preparation of intermediate 4: Intermediate **3** (1.40 g, 5 mmol, 1 eq) and K_2CO_3 (1.38 g, 2 eq) were added into a round bottom flask, then MeOH (20 mL) was added, and stirred under room temperature for 12 h. The reaction mixture was diluted with water (200 mL), extracted with Et_2O (3×). The combined organics were dried (Na_2SO_4), filtered and evaporated to afford intermediate **4** as a yellow oil (0.91 g, 87%).

Dextran-N$_3$ was prepared according to the reported procedure. All characterizations of intermediate **5** and **6** can be found in reference.[3]

Preparation of intermediate 5: 3-bromopropan-1-amine hydrobromide (3.28 g, 15 mmol, 1 eq) and NaN_3 (3.32 g, 51 mmol, 3.4 eq) were added into a round bottom flask, dissolved with water (25 mL), stirred, and heated to reflux for 15 h. The reaction mixture was cooled to room temperature, Et_2O (25 mL) was added and cooled to 0 °C, then KOH (4 g) was added. After KOH was totally dissolved, another 25 mL Et_2O was added and the organic phase was then separated. The water phase was further extracted with Et_2O (2×). The combined organics were dried (Na_2SO_4), filtered and evaporated carefully to afford intermediate **5** as a yellow

liquid (608 mg, 40%).

Preparation of intermediate 6: Intermediate **5** (1.8 g, 18 mmol, 1 eq) was added into a round bottom flask, dissolved with DCM (60 mL) and stirred under room temperature, then a solution of bis(4-nitrophenyl) carbonate (5.4 g, 18 mmol, 1 eq) in DCM (40 mL) was added dropwise in 10 min, and further stirred for 4 h. A solution of NaOH (792 mg, 19.8 mmol, 1.1 eq) in water (60 mL) was added and stirred, then organic phase was separated, dried (Na_2SO_4), filtered, evaporated and purified with silica gel chromatography (PE/EA, 3/1, v/v) to afford intermediate **6** as a white solid (2.57 g, 54%).

Preparation of Dextran-N_3: High vacuum dried Dextran-40000 (2 g, 0.05 mmol, 1 eq) and LiCl (2 g) was dissolved with dry DMF in a dried round bottom flask under N_2 atmosphere and stirred. DIPEA (2 mL) and intermediate **6** (172 mg, 0.65 mmol, 13 eq) was then added. The reaction mixture was heated to 70 ºC and further stirred for 24 h, then cooled down and concentrated to dryness, dissolved with water and dialyzed against water for 3 days with 3× per day exchange of dialysate for fresh water. The water solution was finally lyophilized to afford Dextran-N_3 as a white solid (1.76 g, 85%). The amount of N_3 group in each Dextran-N_3 molecule was quantified to be 11.97 based on ^{1}H-NMR.

Preparation of AF3-Alkyne: Method 2 was applied. intermediate **4** (313 mg, 1.5 mmol, 1 eq), *n*-butyllithium (1.575 mL, 2 M in cyclohexane, 2.1 eq, added in excess of 1 equivalent to neutralize the alkynyl-H in intermediate **4**) and 3,6-bis(dimethylamino)-9H-fluoren-9-one (**1c**; 133 mg, 0.5 mmol) were used, affording AF3-Alkyne as a brown solid (119 mg, 50%, perchlorate salt). ^{1}H NMR (400 MHz, $CDCl_3$) δ 7.64 (s, 2H), 7.27 (s, 2H), 6.54 (d, J = 8.9 Hz, 2H), 6.08 (d, J = 8.9 Hz, 2H), 3.44 (s, 12H), 3.11 (s, 1H), 2.19 (s, 6H); ^{13}C NMR (101 MHz, $CDCl_3$) δ 172.04 (C), 158.60 (C), 147.82 (C), 136.04 (C), 132.92 (C), 131.93 (C), 131.46 (C), 129.59 (C), 123.17 (C), 113.94 (C), 111.47 (C), 83.23 (C), 78.26 (CH), 42.40 (CH3), 20.12 (CH3); Maldi-Tof/Tof-MS calcd for $C_{27}H_{27}N_2$ $[M]^+$ 379.2169, found 379.2277.

Preparation of AF3-Dextran: Dextran-N_3 (59 mg, 0.0014 mmol, 1 eq), AF3-Alkyne (8 mg, 0.0168 mmol, 12 eq), copper(II) sulfate pentahydrate (4.2 g, 0.0168 mmol, 12 eq) and DMSO (5 mL) was added in a dried round bottom flask under N_2 atmosphere and stirred under room temperature, then sodium ascorbate (3.9 mg, 0.0192 mmol, 14 eq) was added and the reaction mixture was further stirred for 24 h. The solution was dialyzed against water (3 days, 3× water exchange per day) and lyophilized to afford AF3-Dextran as a brown solid (60 mg, 81%). The amount of AF3 in each AF3-Dextran molecule was measured to be 4.7 according to the absorption spectra (Using the absorption coefficient of AF3 as a reference, the peak absorbance of an AF3-Dextran PBS solution was measured at 0.367, which spectrally correlated to 17.7 μM AF3. Then 50 mL

of this solution (containing 0.885 μmol AF3) underwent lyophilized and was then weighed, registering a mass of 8.1 mg. Considering the molecular weight of AF3-Dextran as 43000 Da and the amount of AF3-Dextran was 0.188 μmol, then the average number of AF3 groups per molecule can be calculated to be 0.885/0.184=4.7).

Supplementary Tables

Supplementary Table 1 | Molecular weights, absorption/emission wavelengths and wavelength-to-molecular-weight ratios of AF dyes and the fluorescent dyes in Figure 1.

Dye	Molecular weight (g/mol)	λ_{abs} (nm)	λ_{em} (nm)	Wavelength-to-molecular-weight ratio (λ_{abs}/Mw)
AF1	299	800	976	2.676
AF2	327	847	933	2.590
AF3	355	959	1014	2.701
AF4	387	983	1046	2.540
AF5	411	966	1015	2.350
AF6	379	962	1014	2.538
AF7	451	946	995	2.098
AF8	407	968	1017	2.378
AF9	435	966	1026	2.221
AF10	463	973	1026	2.102
AF11	495	994	1045	2.008
AF12	479	965	1029	2.015
AF13	603	1045	1196	1.733
AF14	503	1101	1187	2.189
1	851	907	546	1.559
2	701	816	446	1.572
3	641	659	309	2.074
4	646	660	399	1.619
5	721	740	531	1.358
6	700	728	391	1.790
7	732	745	362	2.022
8	654	744	474	1.380
9	817	853	424	1.927
10	880	960	659	1.335
11	880	921	711	1.238
12	665	683	284	2.342
13	621	679	440	1.411
14	596	740	299	1.993
15	668	770	324	2.062
16	686	710	770	0.891
17	723	738	552	1.310
18	848	868	562	1.509
19	636	658	646	0.985
20	688	701	534	1.288
21	910	1060	747	1.218
22	625	698	600	1.042
23	602	618	412	1.461
24	688	715	557	1.235
25	742	764	521	1.424
26	775	831	751	1.032
27	981	1032	547	1.793
28	1046	1080	742	1.410
29	1080	1130	610	1.770
30	650	684	511	1.272
31	746	766	537	1.389
32	862	908	563	1.531
33	1026	1045	664	1.545
34	1072	1103	1019	1.052
35	700	743	439	1.595
36	630	807	603	1.045
37	666	736	495	1.345
38	1014	1070	652	1.555
39	932	980	509	1.831
40	709	975	680	1.043
41	879	1120	845	1.040
42	763	1065	800	0.954
43	932	1230	894	1.043
44	750	1055	969	0.774
45	627	633	314	1.997
46	698	705	514	1.358
47	745	750	714	1.043

Supplementary Table 2 | Photophysical properties of AF3 in different solvents.

Solvent	λ_{abs} (nm)	ε (M^{-1} cm^{-1})	λ_{em} (nm)	Stokes shift (nm)	Quantum yield (10^{-2} %)
DCM	959	23052	1014	55	1.1
Toluene	946	27288	993	47	2.0
Dioxane	940	23765	997	57	1.4
DMF	949	18773	1015	66	0.9
DMSO	951	19618	1022	71	1.1
EtOH	950	20368	1006	55	0.7
PBS	942	20764	1022	80	0.4

Supplementary Table 3 | Crystal data and structure refinements for AF3.

Compound	AF3
CCDC No.	2263552
Formula	$C_{13}H_{14.50}Cl_{1.50}NO_2$
Formula wt.	269.93
T (K)	173
Wavelength (Å)	1.34138 Å
Crystal size	0.23×0.16×0.03
Crystal system	Monoclinic
Space group	$P2_1/n$
a/Å	11.2051 (4)
b/Å	8.7168 (3)
c/Å	25.7043 (10)
α (deg.)	90
β (deg.)	91.984 (2)
γ (deg.)	90
Volume/Å^3	2509.10 (16)
Z	8
Density (calcd.)	1.429
μ (mm^{-1})	2.388
θ (deg.)	2.99 to 59.40
F(000)	1128
Completeness	1.000
Goodness-of-fit	1.045
final R indices ($I>2\sigma(I)$)	0.0428/0.1158
R indices (all data)	0.0495/0.1220
largest diff. peak and hole (e Å^{-3})	0.491/-0.515

Supplementary Table 4 | MCI indexes (in electrons) for AF3 in the S_0, S_1, and T_1 states computed at various levels.

Methods	S_0	S_1	T_1
LC-BLYP*	0.1025	0.1030 (ΔSCF)<br>0.1051/0.1039 (TD/TDA) [a]	0.1126 (UDFT) [b]<br>0.1092 (TDA) [a]
B3LYP	0.0654	0.0670 (TD)	0.0726 (UDFT) [b]
CASSCF(2,2)	0.0159	0.0183	0.0224

[a]the Tamm−Dancoff approximation (TDA) scheme of TD-DFT is shown to reliably predict triplet excited state versus traditional TD-DFT. [b]unrestricted density functional theory (UDFT).

Supplementary Table 5 | Molecular weight, net charge and cLog D of modified and unmodified AF dyes. cLog D was calculated using ChemAxon software.

Name	Molecular weight (Da)	Net charge (pH = 7)	cLogD (pH = 7)
AF2	327	+1	1.92
AF2-COOH	370	0	-2.27
AF3	355	+1	3.30
AF3-COOH	399	0	-0.89
AF3-Dextran	~43300	—	—

Supplementary Table 6 | Calculated $NICS(1)_{zz}$ values (ppm) based on the central five-membered ring of AF3 using three methods including B3LYP, LC-BLYP* and CASSCF(2,2) with a 6-31G(d) basis set. Note that $NICS(1)_{zz}$ was defined as the zz component of the NICS value located 1 angstrom perpendicular to the plane above the five-membered ring. The CASSCF calculations were performed using Dalton 2022 software.[41,42]

State	B3LYP	LC-BLYP*	CASSCF(2,2)
S0	34.42	37.33	32.07
S1	-23.87	-25.30	-66.04

Supplementary Figures

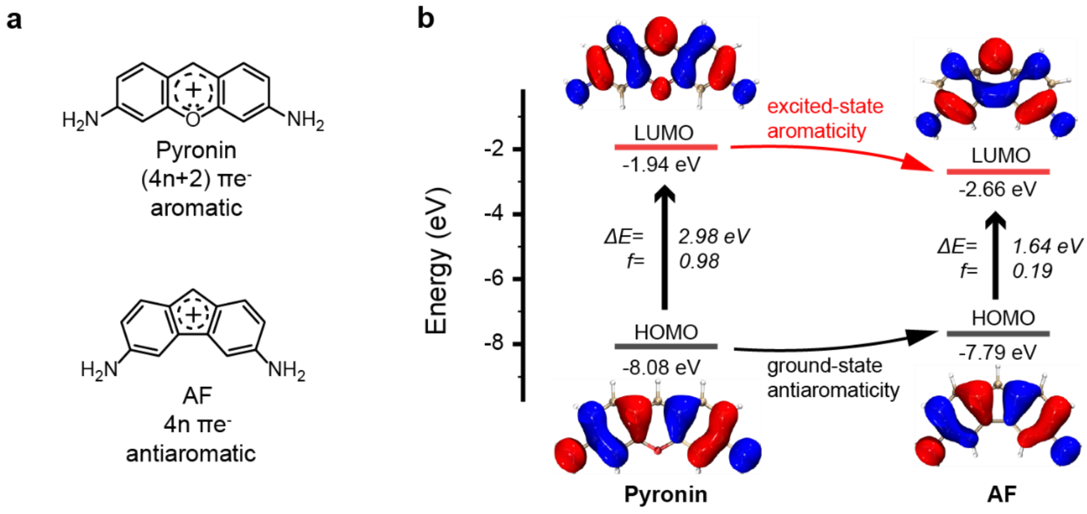

Supplementary Figure 1 | Schematic illustration of aromatic and antiaromatic molecular skeleton. a, Schematically shows the structure of AF skeleton with a 4nπ-electron five-membered ring as an antiaromatic core and corresponding pyronin skeleton with a (4n+2)π-electron aromatic core. **b,** HOMO/LUMO energy level and ΔE comparison of AF and pyronin skeleton shows the gap narrowing effect of antiaromatic skeleton introduction. The calculation was conducted using (TD)DFT at the LC-BLYP*/def-TZVP with DCM in PCM solvent model.

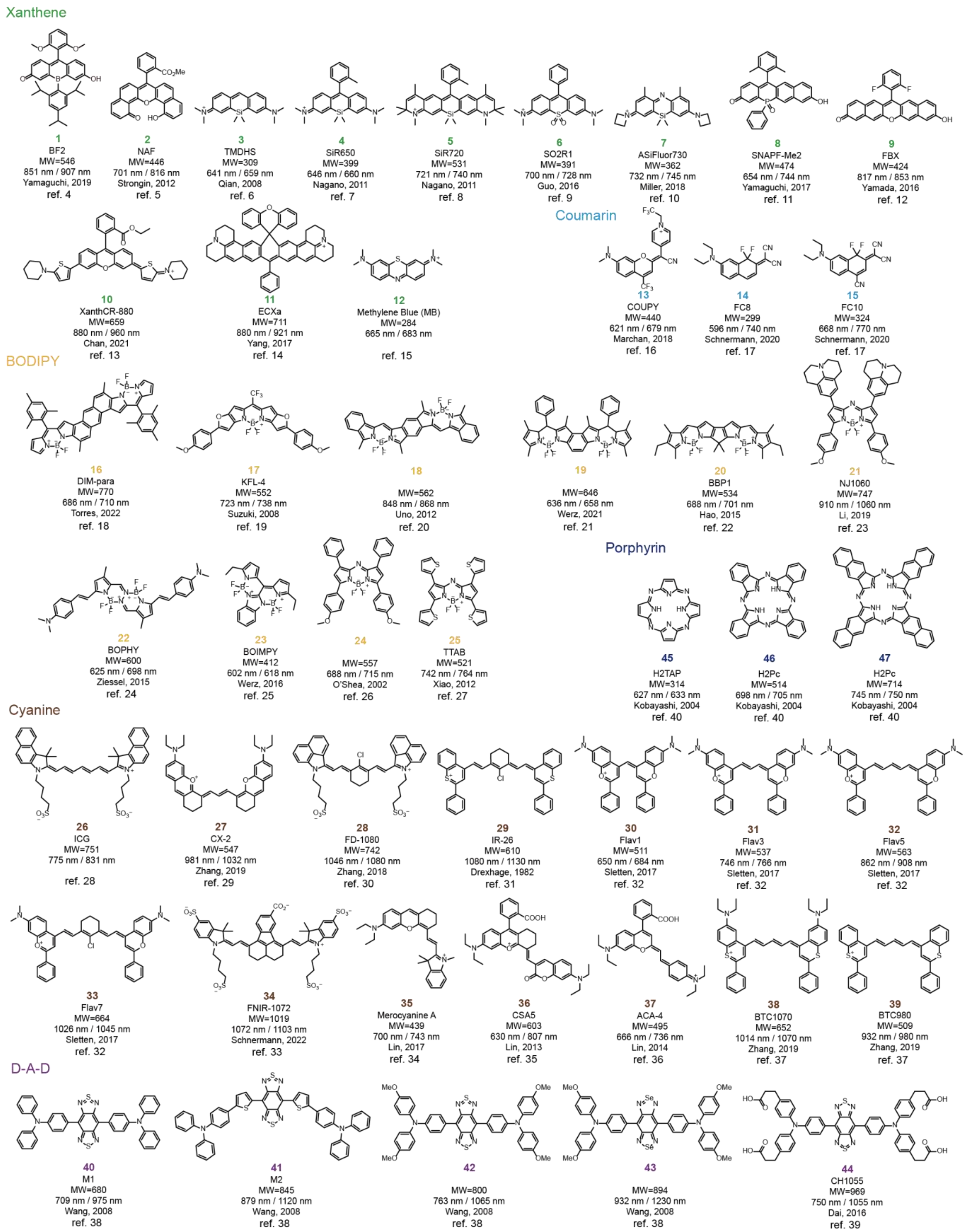

Supplementary Figure 2 | Molecular structures and absorption/emission wavelength of compared fluorescent dyes in Figure 1.

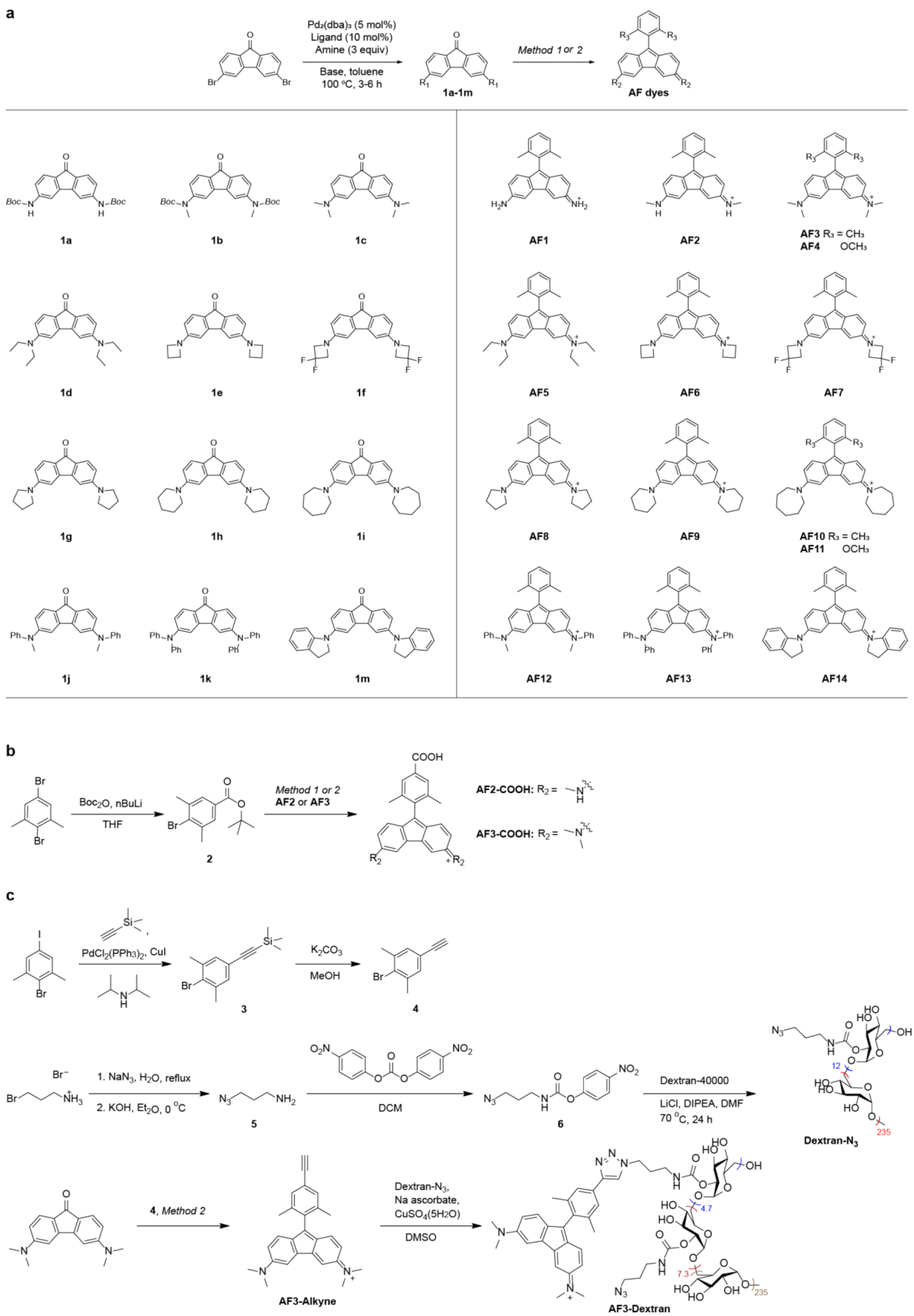

Supplementary Figure 3 | Synthetic route of AF dyes and derivatives. a, Synthetic route and corresponding structure of AF dyes. **b,** Synthetic route of AF2-COOH and AF3-COOH. **c,** Synthetic

route of AF3-Dextran.

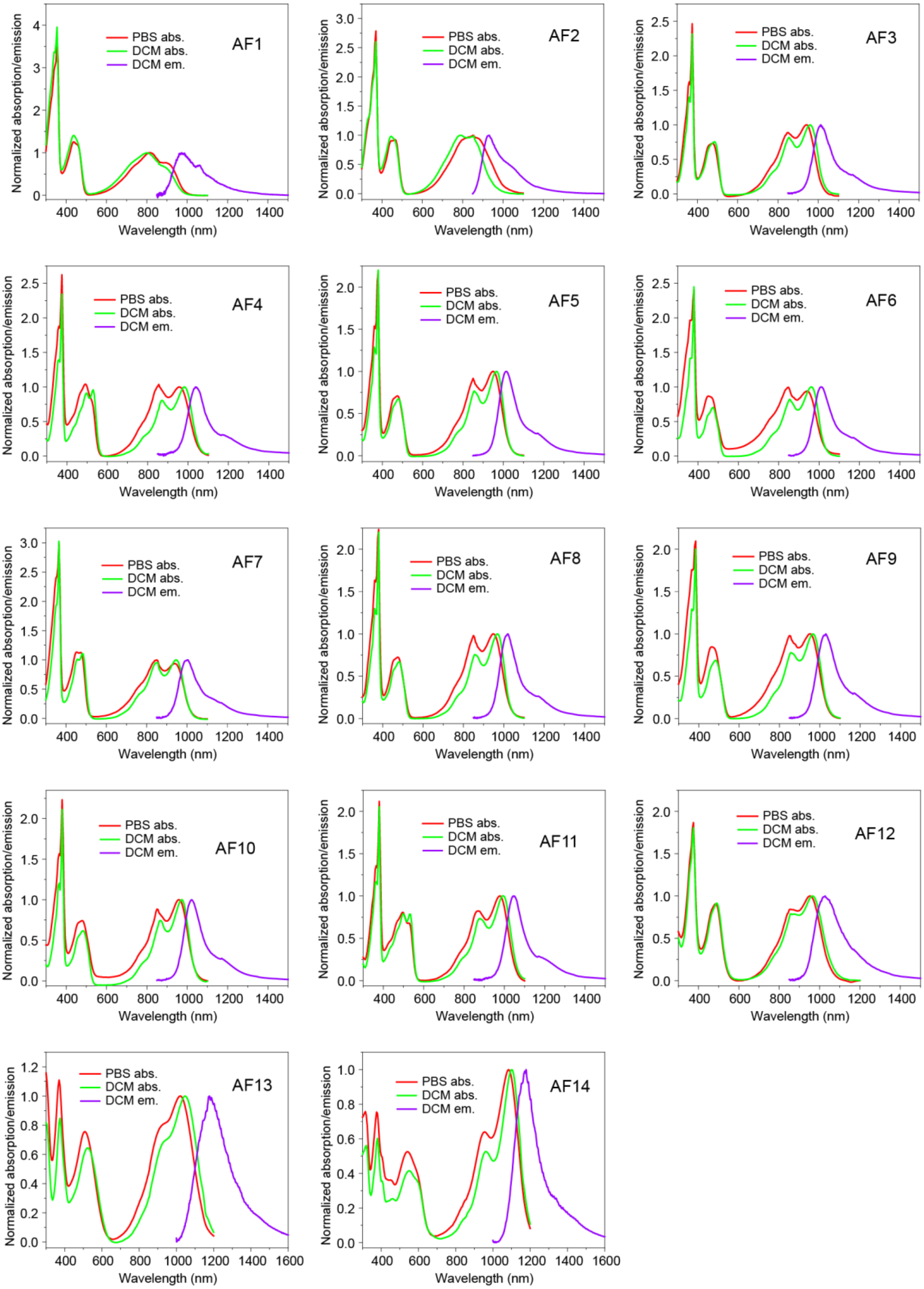

Supplementary Figure 4 | Normalized absorption spectra and emission spectra of AF1-AF14. Absorption spectra were collected in 1×PBS 7.4 solution and DCM, emission spectra were collected

in DCM under 808 nm excitation.

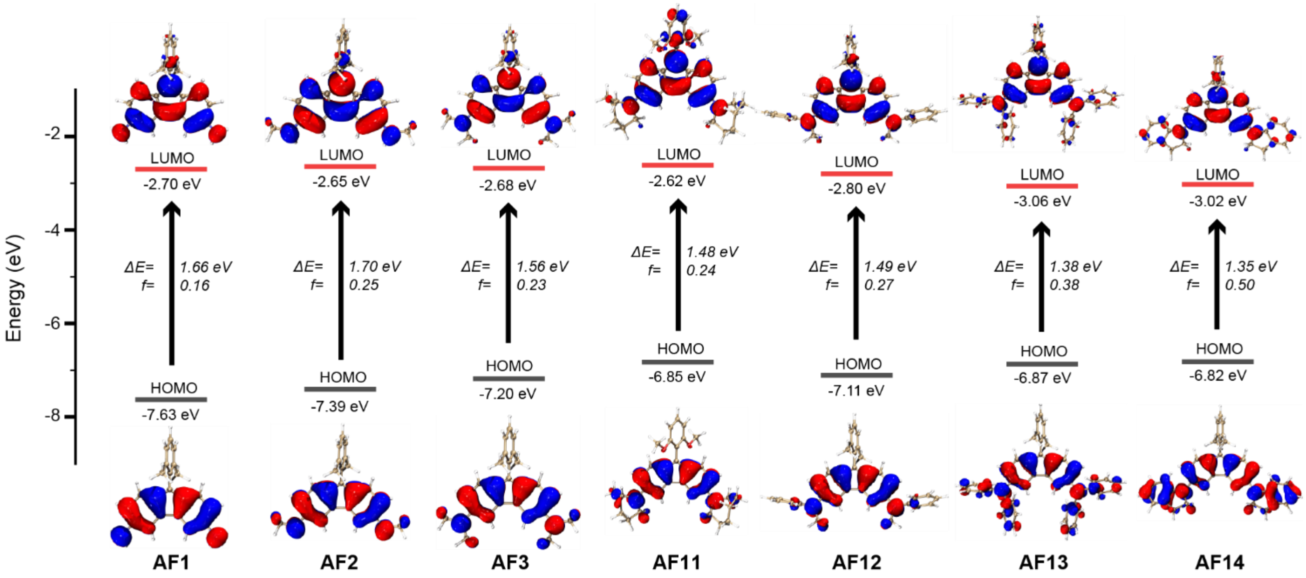

Supplementary Figure 5 | Comparison of the HOMO and LUMO energy levels, S_0-S_1 excitation energies and oscillator strengths for AF1, AF2, AF3, AF11, AF12, AF13 and AF14 based on TDDFT calculations at the LC-BLYP*/def-TZVP with DCM in PCM solvent model.

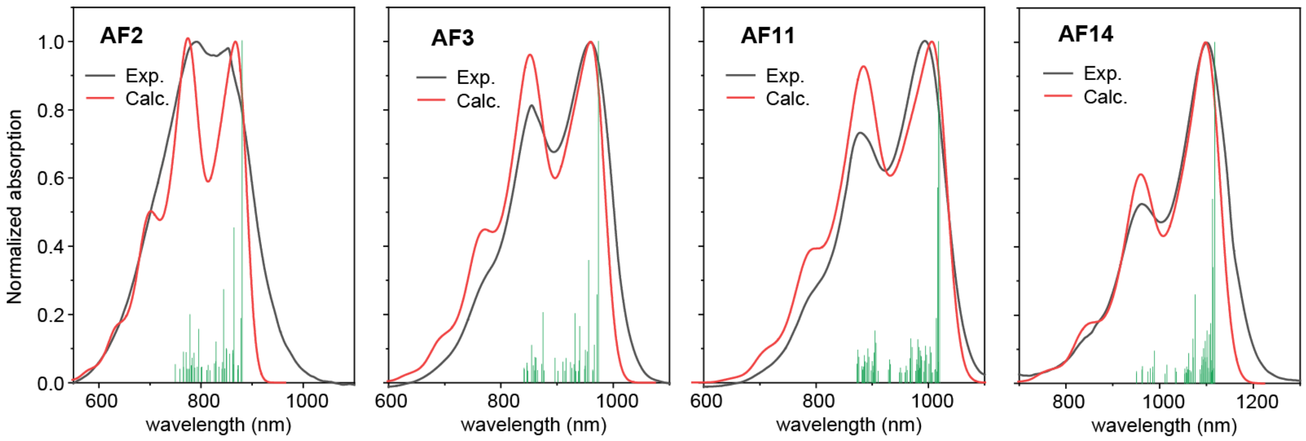

Supplementary Figure 6 | Vibration resolved absorption spectra (red) and experimental spectra (black) of AF dyes. Green sticks show the normalized strength of each mainly contributed vibration resolved transition. Gaussian broadening was applied for the calculated spectra and half width at half maximum (HWHM) was set to 225 cm^{-1}. The calculated results were shifted (AF3: +20 nm; AF14: +10 nm) to fit the experimental results better.

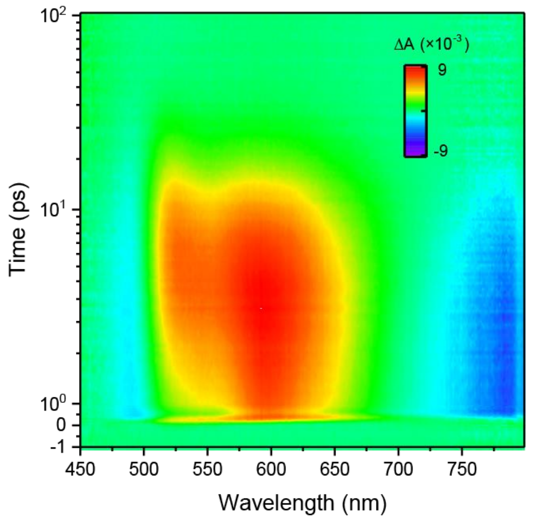

Supplementary Figure 7 | Femtosecond transient absorption contour maps of AF3 under 375 nm pump excitation.

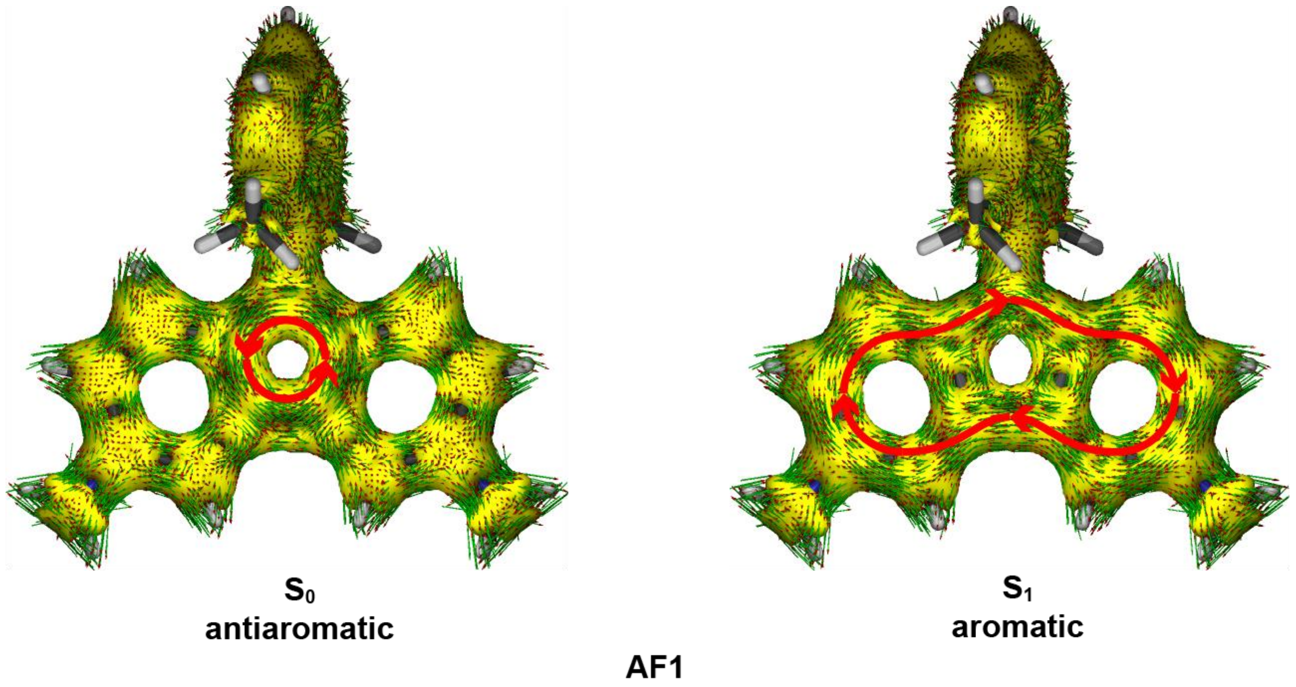

Supplementary Figure 8 | Theoretical calculated AICD diagram of AF1 in S_0 and S_1 state.

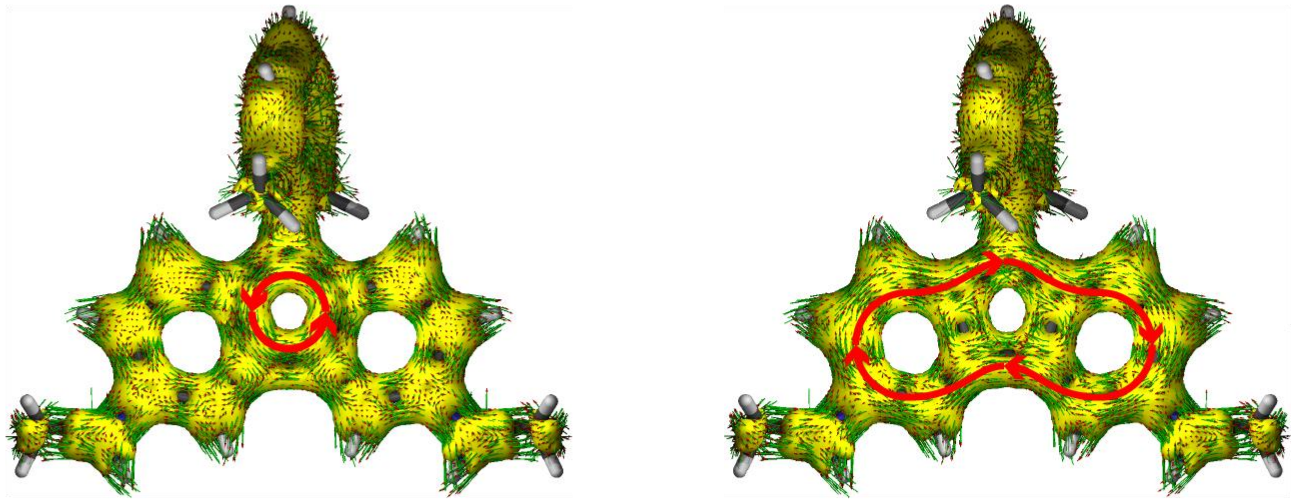

S_0
antiaromatic

S_1
aromatic

AF2

Supplementary Figure 9 | Theoretical calculated AICD diagram of AF2 in S_0 and S_1 state.

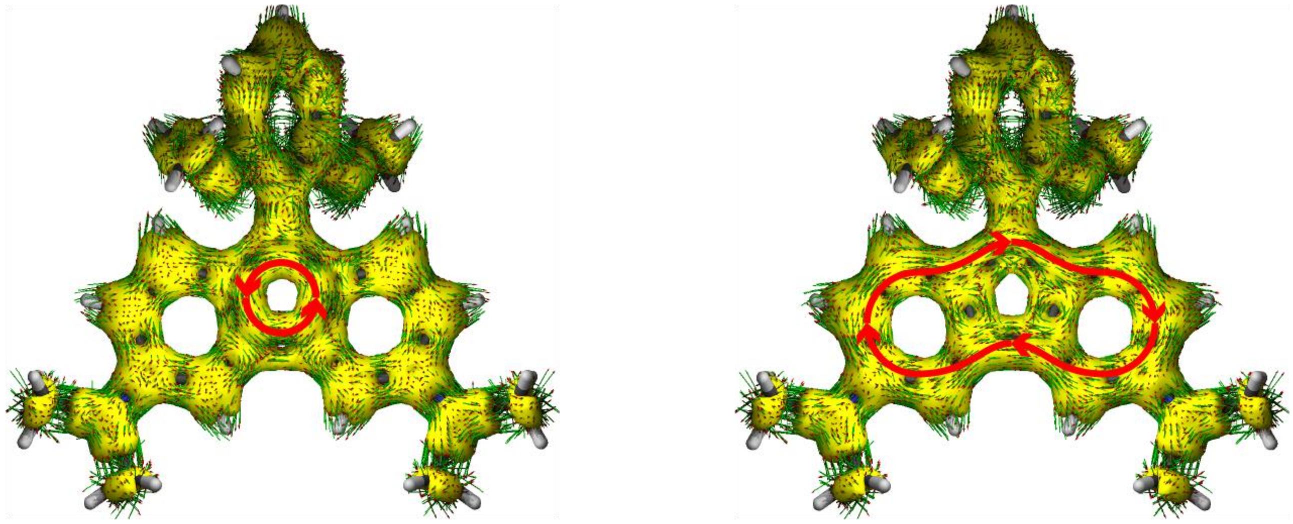

S_0
antiaromatic

S_1
aromatic

AF4

Supplementary Figure 10 | Theoretical calculated AICD diagram of AF4 in S_0 and S_1 state.

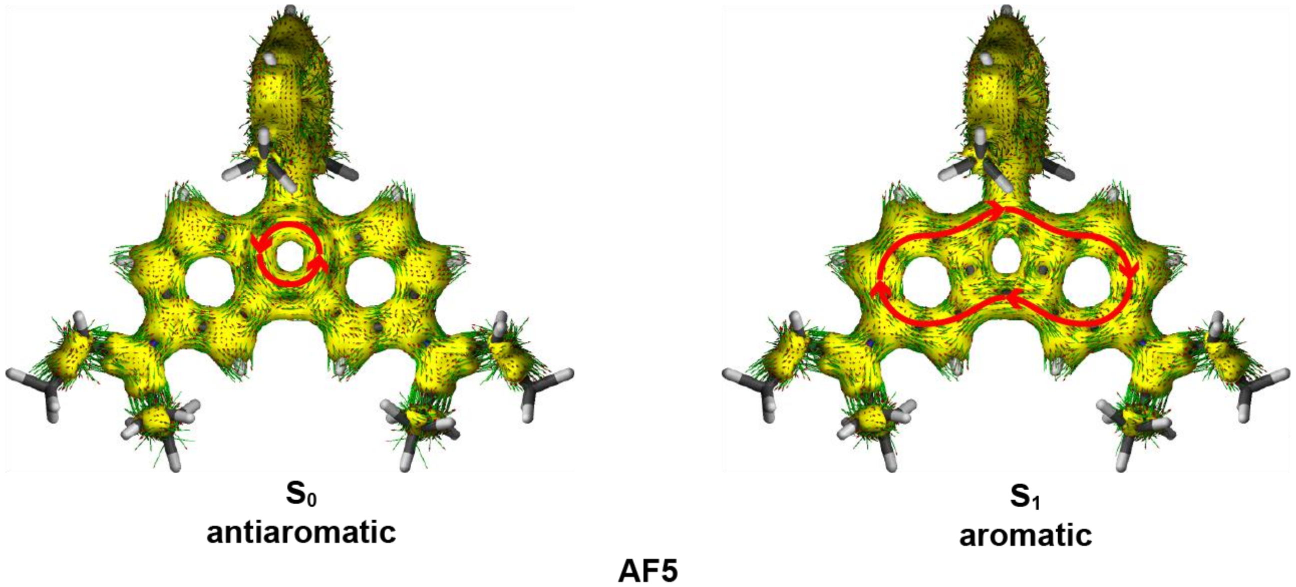

Supplementary Figure 11 | Theoretical calculated AICD diagram of AF5 in S_0 and S_1 state.

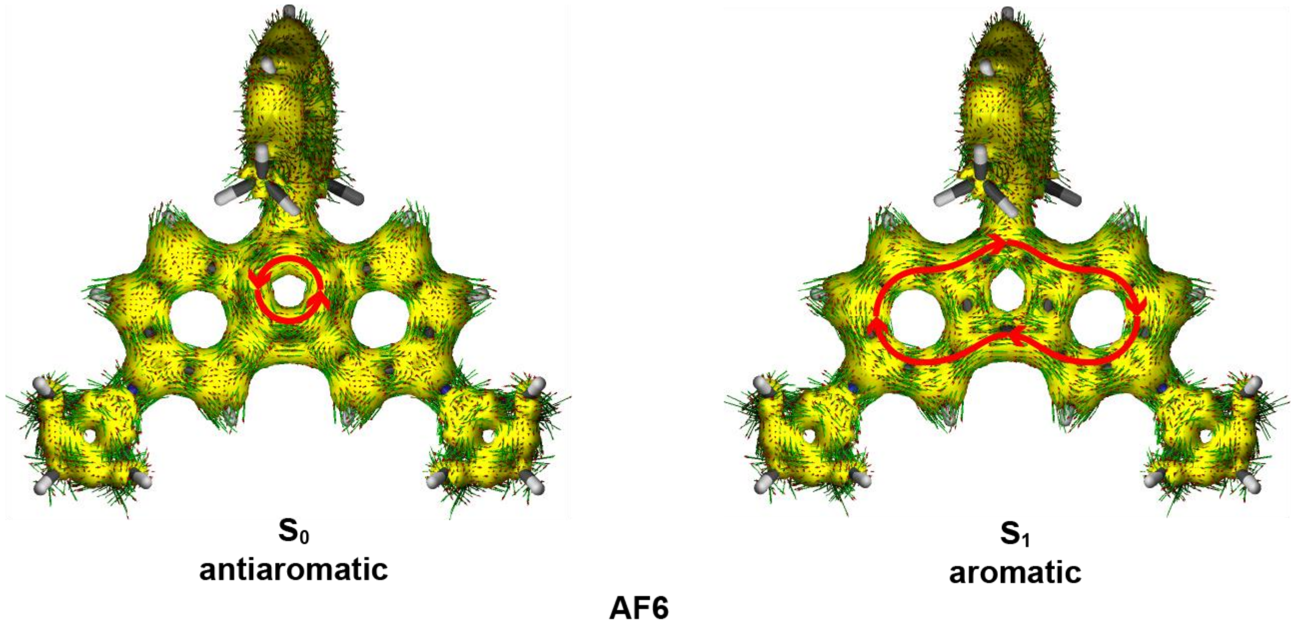

Supplementary Figure 12 | Theoretical calculated AICD diagram of AF6 in S_0 and S_1 state.

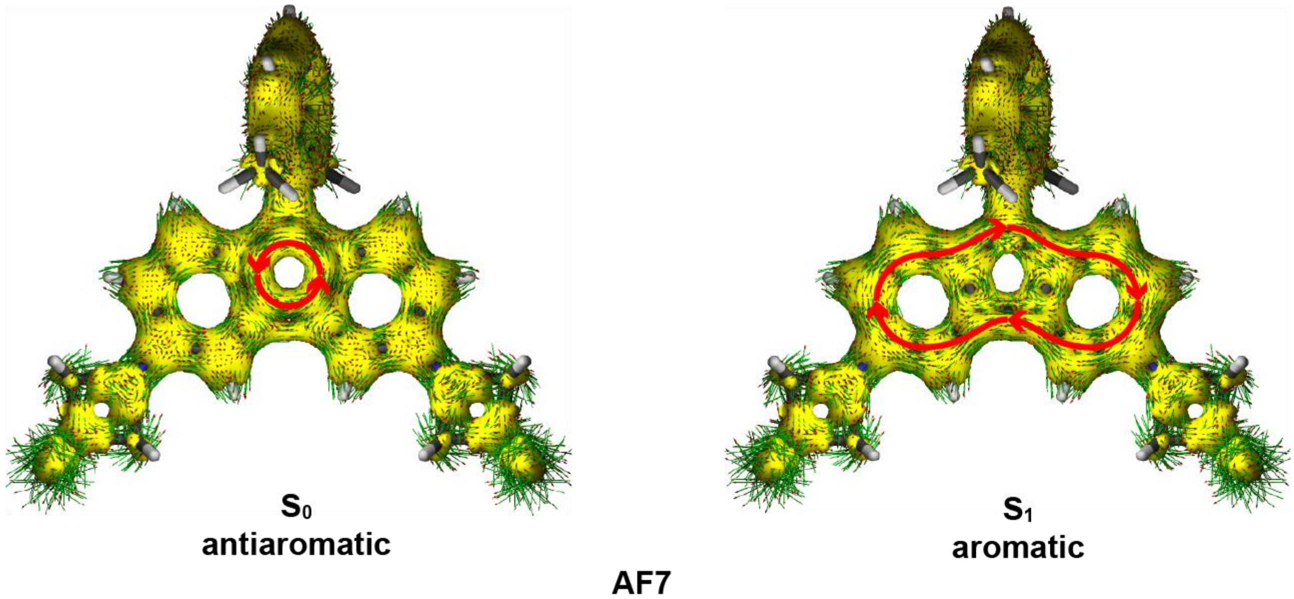

Supplementary Figure 13 | Theoretical calculated AICD diagram of AF7 in S_0 and S_1 state.

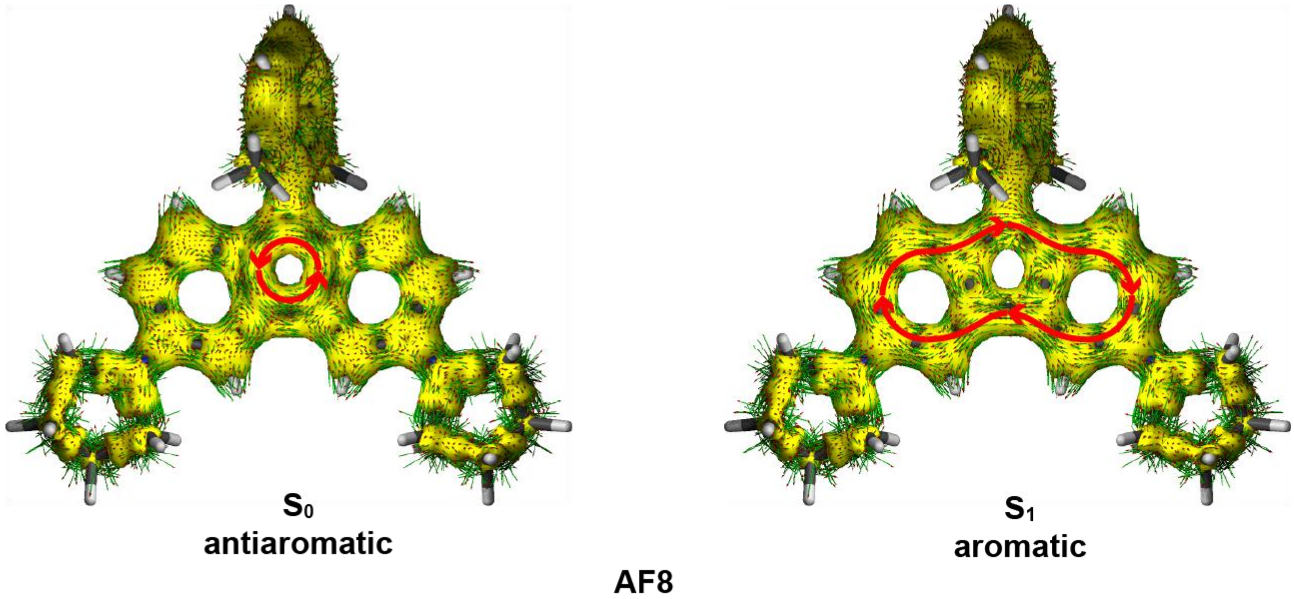

Supplementary Figure 14 | Theoretical calculated AICD diagram of AF8 in S_0 and S_1 state.

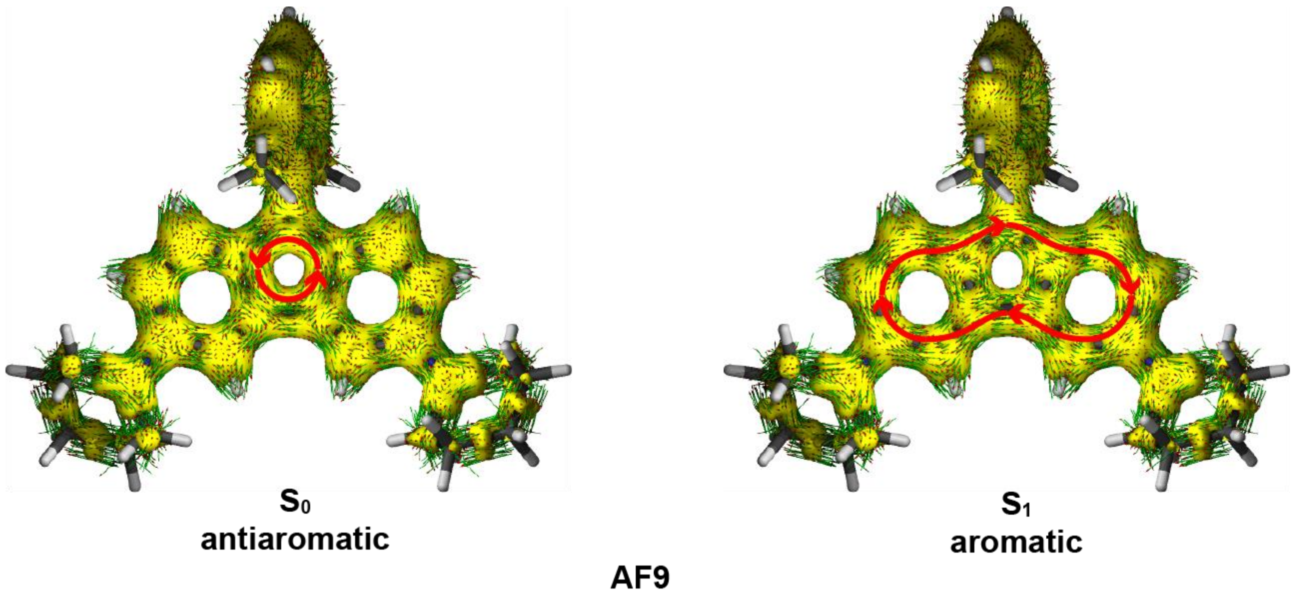

Supplementary Figure 15 | Theoretical calculated AICD diagram of AF9 in S_0 and S_1 state.

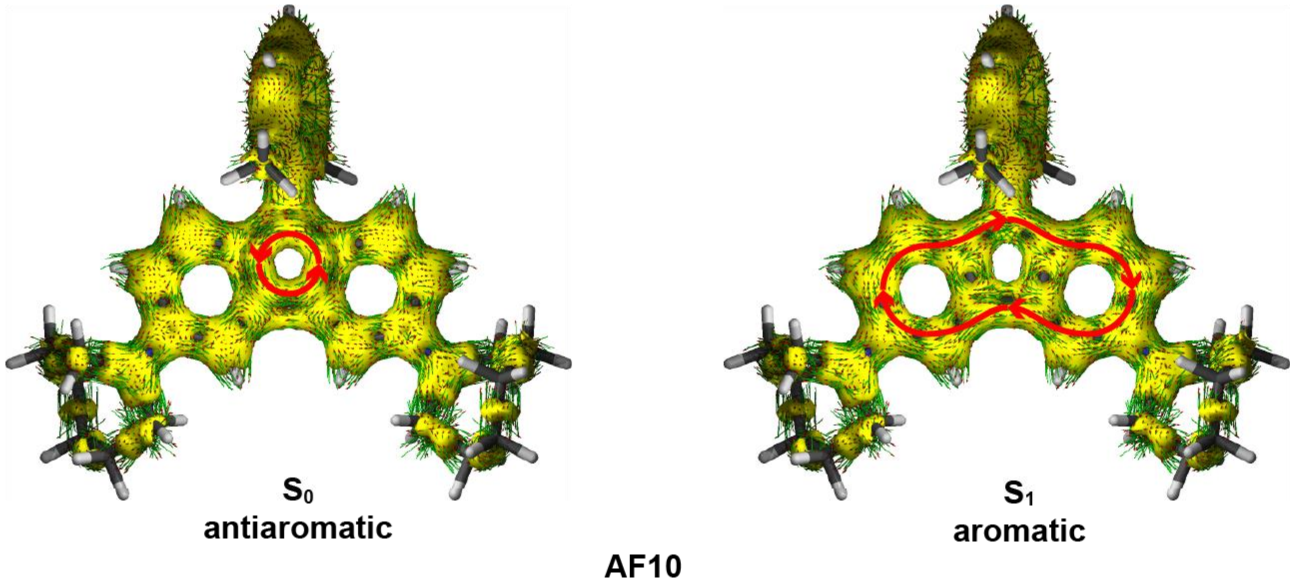

Supplementary Figure 16 | Theoretical calculated AICD diagram of AF10 in S_0 and S_1 state.

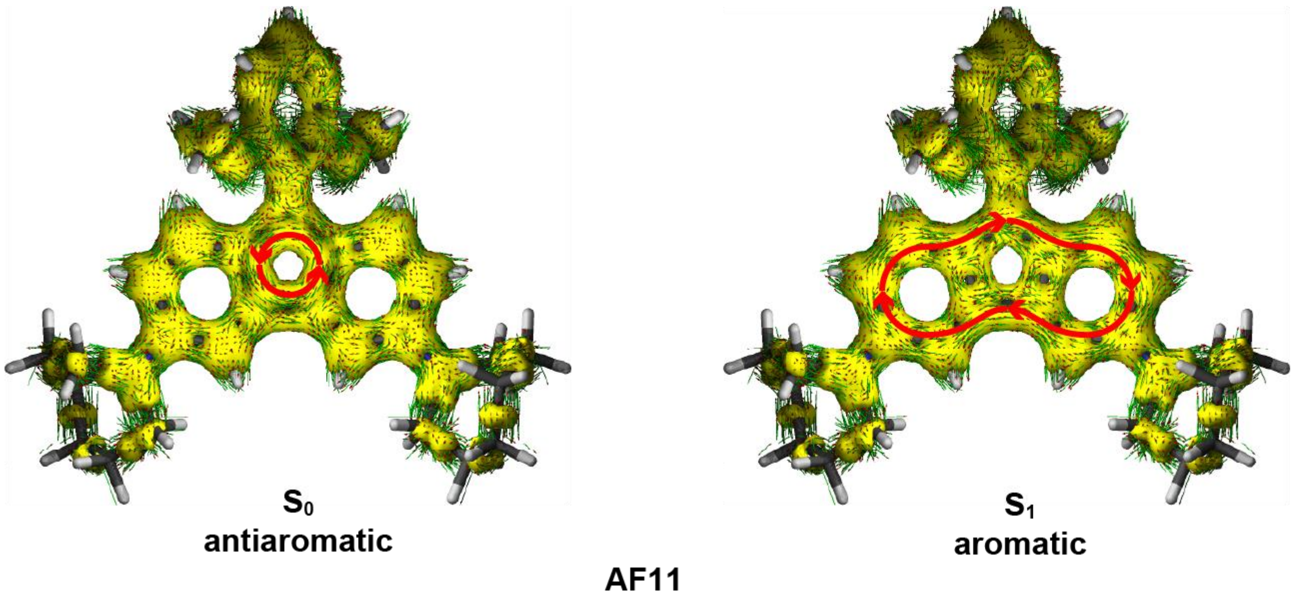

Supplementary Figure 17 | Theoretical calculated AICD diagram of AF11 in S_0 and S_1 state.

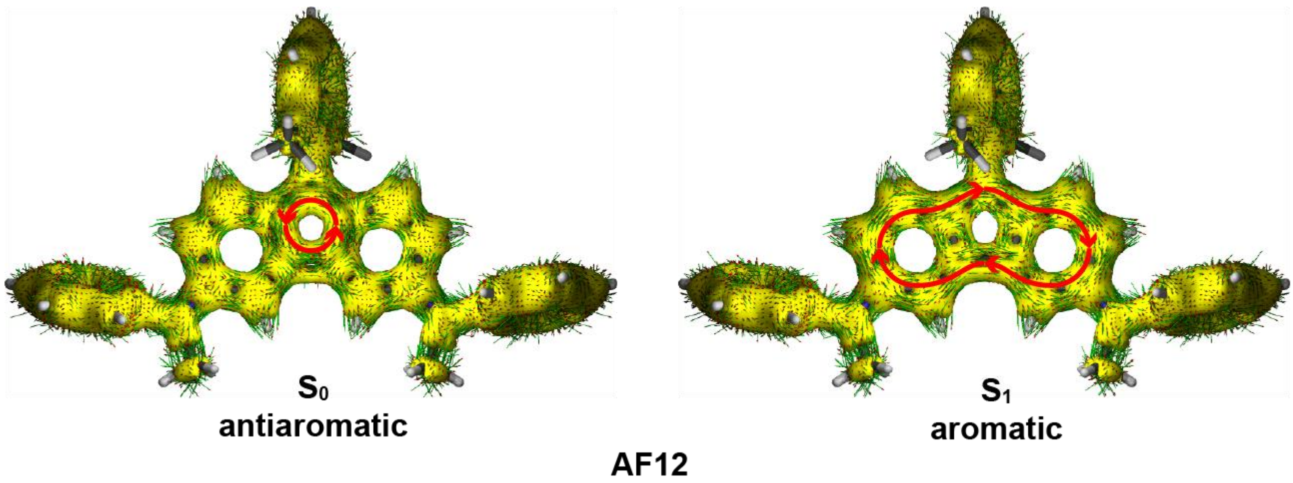

Supplementary Figure 18 | Theoretical calculated AICD diagram of AF12 in S_0 and S_1 state.

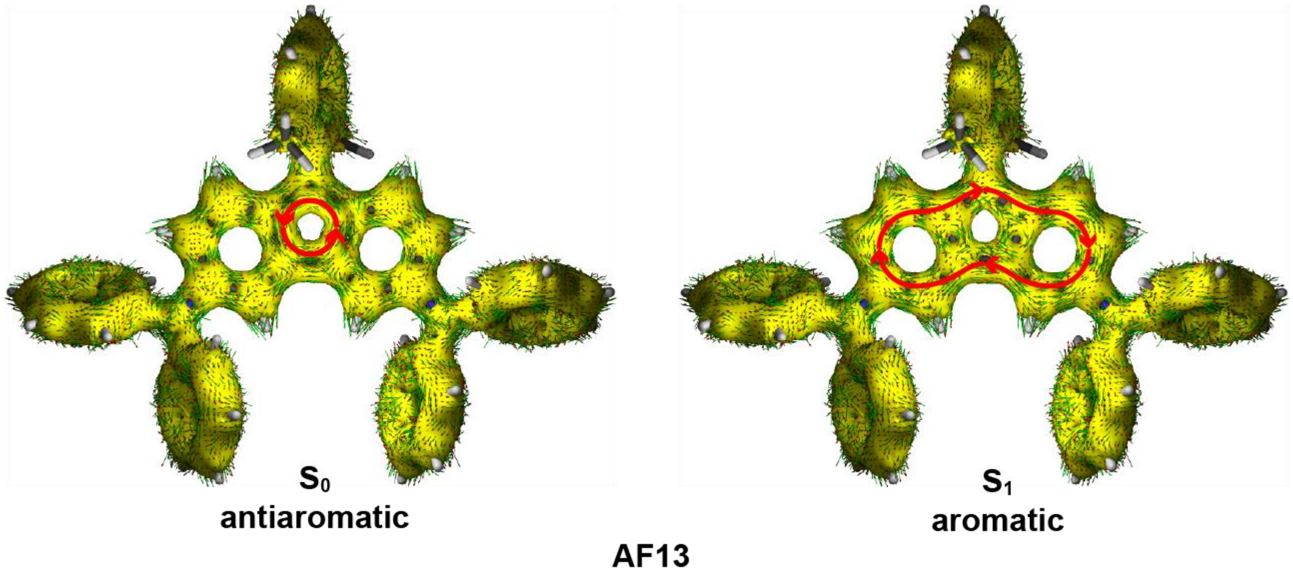

Supplementary Figure 19 | Theoretical calculated AICD diagram of AF13 in S_0 and S_1 state.

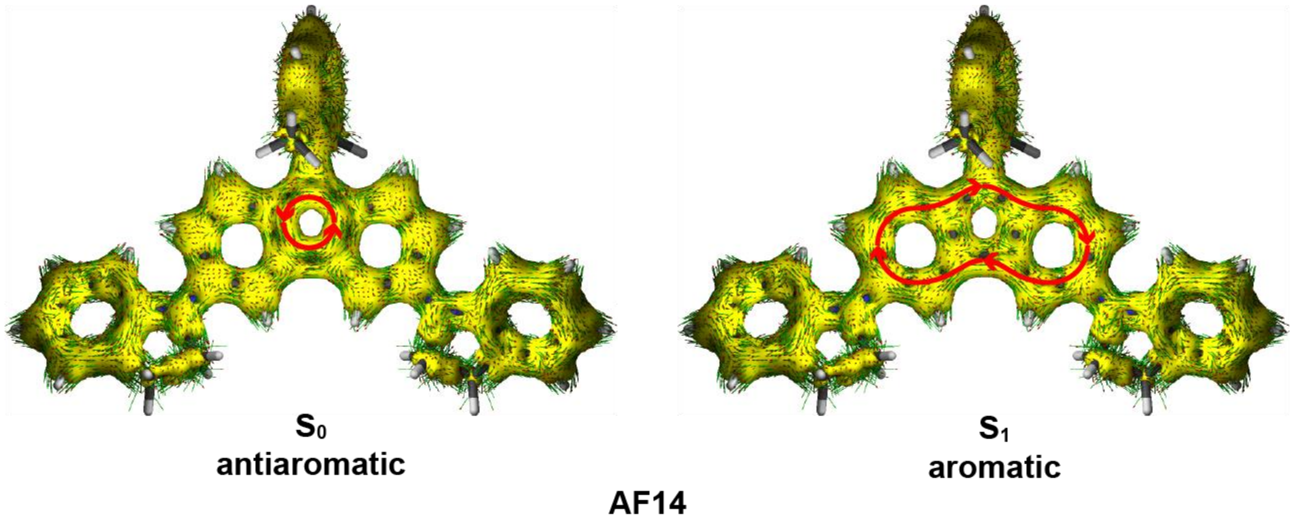

Supplementary Figure 20 | Theoretical calculated AICD diagram of AF14 in S_0 and S_1 state.

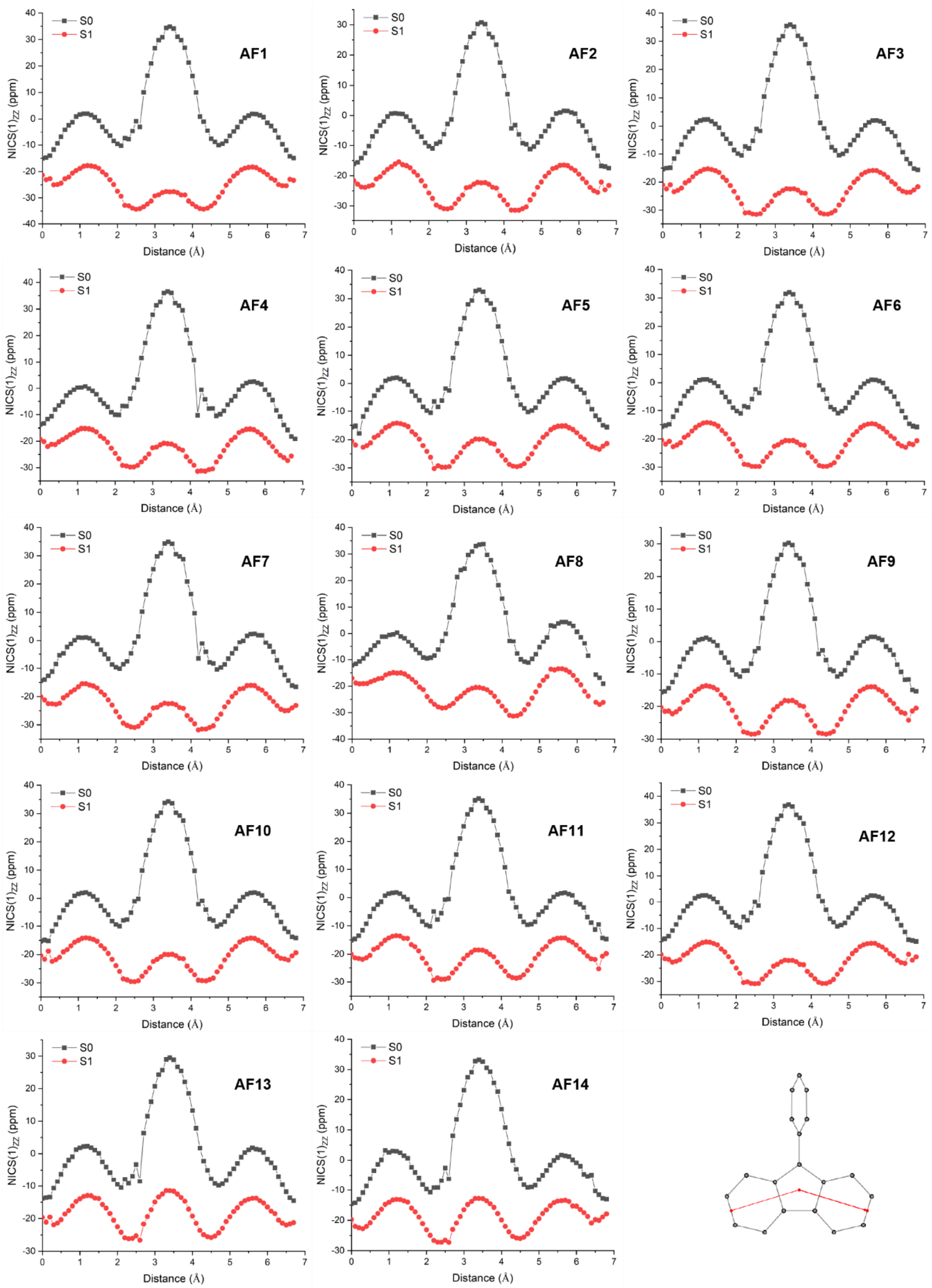

Supplementary Figure 21 | NICS-XY scans of AF dyes in both S_0 (black) and S_1 (red) state. All the scans are performed at a height of 1 Å. The red line in the bottom right image shows the scanning path with a scanning interval of 0.1 Å.

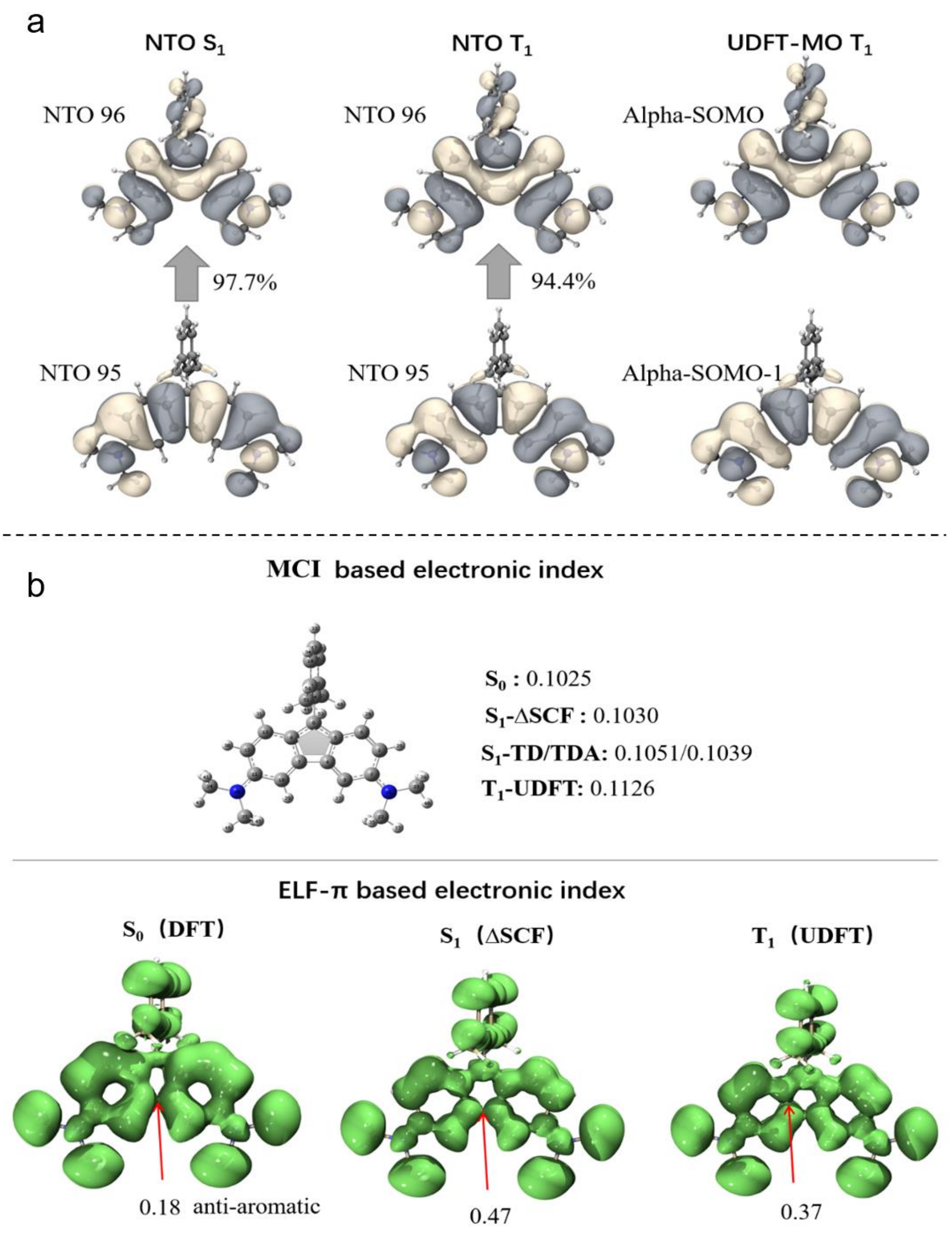

Supplementary Figure 22 | Calculated electronic indexes for (anti-)aromaticity characterization. a, The analysis of the natural transition orbitals (NTOs) using TDA-DFT and the electron configurations based on singly occupied molecular orbitals (SOMOs) using UDFT. **b,** Calculated electronic indexes based on multicenter index (MCI) and electron localization function-π (ELF-π) used for characterizing aromaticity in the five-member ring of AF3 and detailed definition of formula can be found in Lu et al. *J. Comput. Chem.*, 2012, 33, 580 and the manual of Multiwfn code.[43]

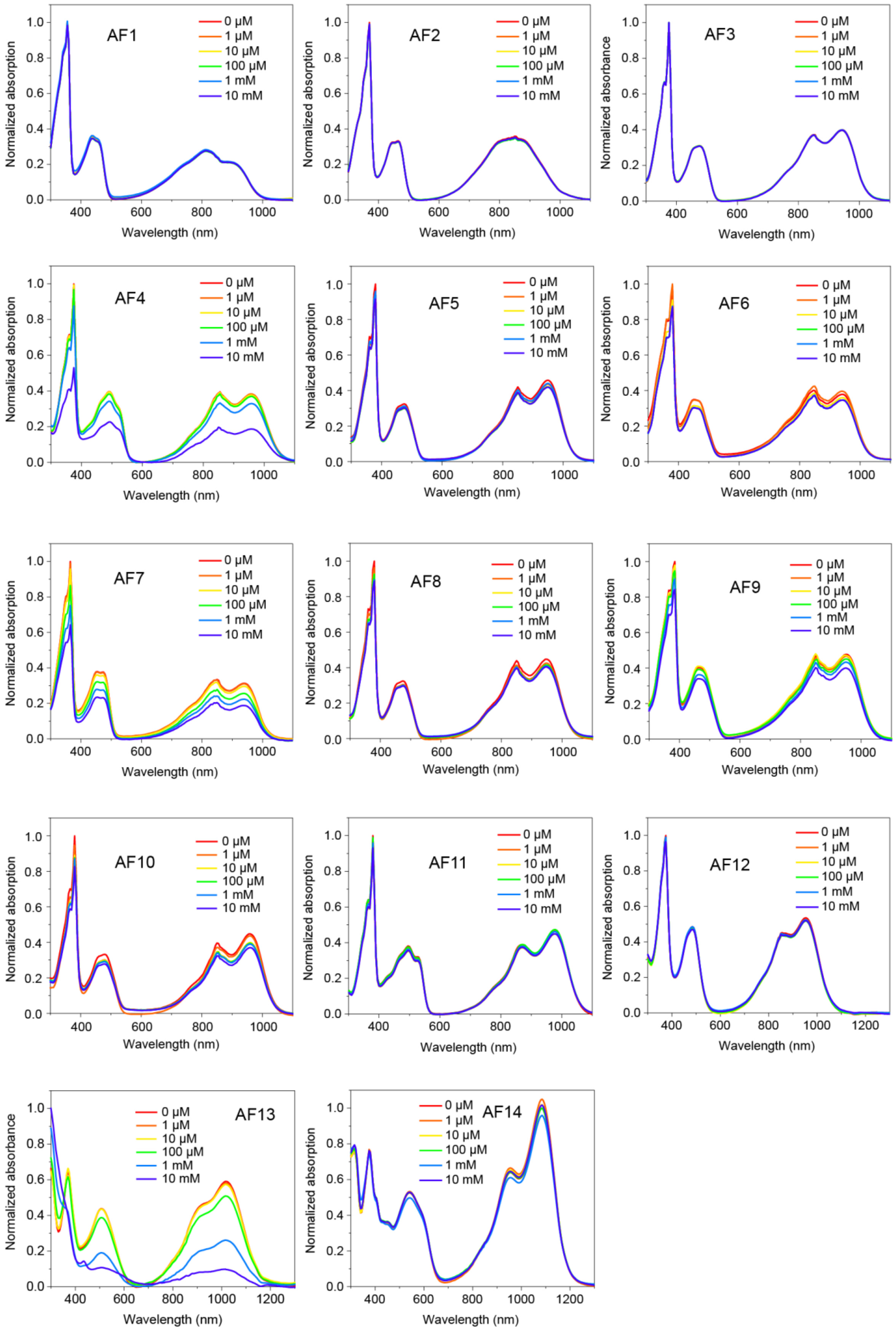

Supplementary Figure 23 | Dose-response curves of all AF dyes as a function of GSH concentration. Note: According to the dose-response curves, we can obtain the dissociation

constant[44] ($K_{d, GSH}$), where the absorbance of the dissociated form is reduced to half of the maximum. Most of AF dyes have $K_{d, GSH}$ values greater than 10 mM. For *o*-dimethyl substituted AF7 and AF13, the decline of $K_{d, GSH}$ could be attributed to the insufficient electron donating effect of N-substituents. Besides, $K_{d, GSH}$ is also slightly reduced at AF4, which could be attributed to the insufficient steric protection effect of *o*-dimethoxyl groups compared to *o*-dimethyl groups. As the cellular concentration of GSH is in the range of 1-10 mM, these AF dyes are stable for use in biological applications.

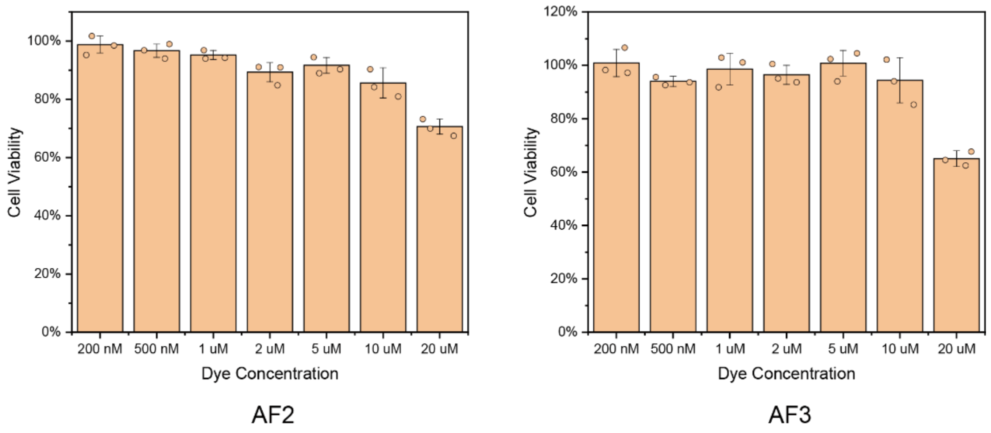

Supplementary Figure 24 | Cell viability results of AF2 and AF3 in 4T1 cells. The bars represent mean ± s.d. derived from n = 3 independent groups.

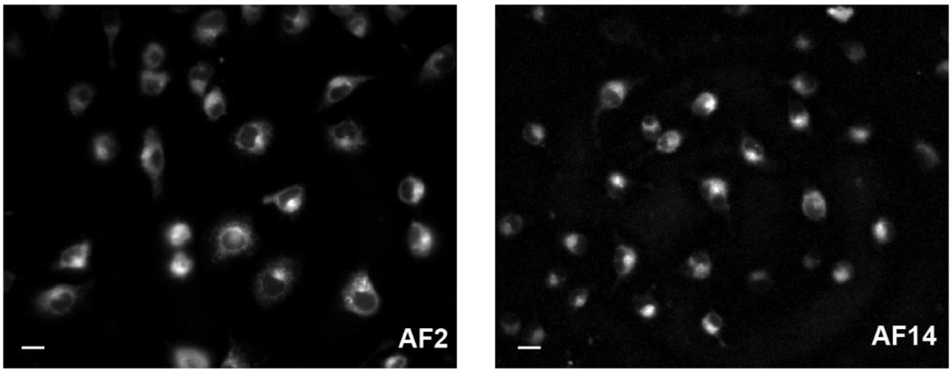

Supplementary Figure 25 | Fluorescence images of AF dye-stained endothelial cells (ECs). Dye concentration: 1 µM in 1×PBS solution. Staining time: 10 min. Fluorescence images were captured under 808 nm excitation and 1000 LP filter. Scale bar, 10 µm.

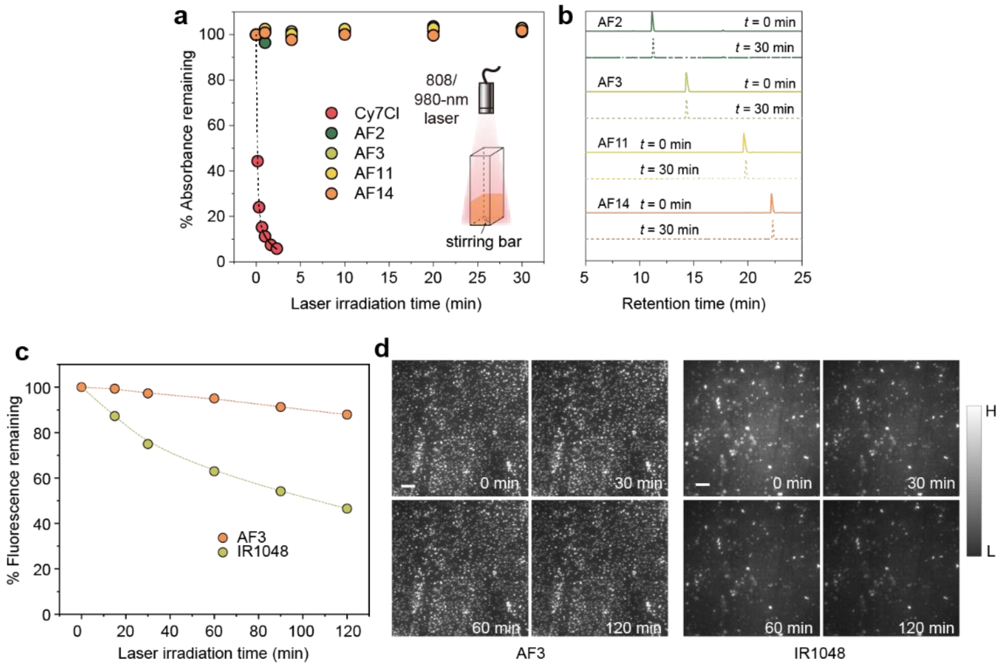

Supplementary Figure 26 | In vitro photostability characterization. a, Photostability comparison of AF2, AF3, AF11, Cy7-Cl (808 nm ex), and AF14 (980 nm ex) in aqueous solution. The absorbance of AF2, AF3, AF11, Cy7-Cl at 808 nm and AF14 at 980 nm were set to 0.5 and laser power density was 1.6 W/cm^2. **b,** HPLC chromatogram of AF dyes before and after irradiation. **c-d,** NIR-II fluorescence microscopic images of dyes exposed to air after certain time irradiation under 808 nm (AF3) and 980 nm (IR1048) laser excitation (**d**), and fluorescence intensity statistic results (**c**). 40×/0.95 objective was used. Laser power density: 200 kW cm^{-2}. Samples were prepared using microscope slides by dropping DCM solution of each dye and slowly evaporating. Scale bar, 10 µm.

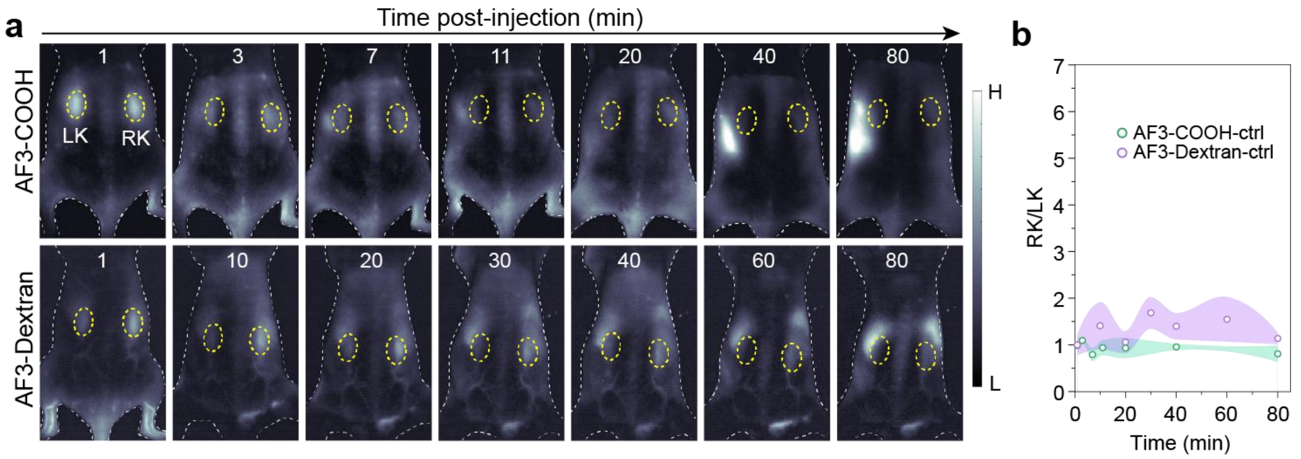

Supplementary Figure 27 | In vivo NIR-II fluorescent imaging of control group mice without renal ischemia-reperfusion treatment. a, In vivo NIR-II fluorescent images of control group mice without renal ischemia-reperfusion treatment. The yellow circles indicate the kidney position. **b,**

Time-dependent evolution of the fluorescence intensity ratio of right-to-left kidney (RK/LK) for images in **a**.

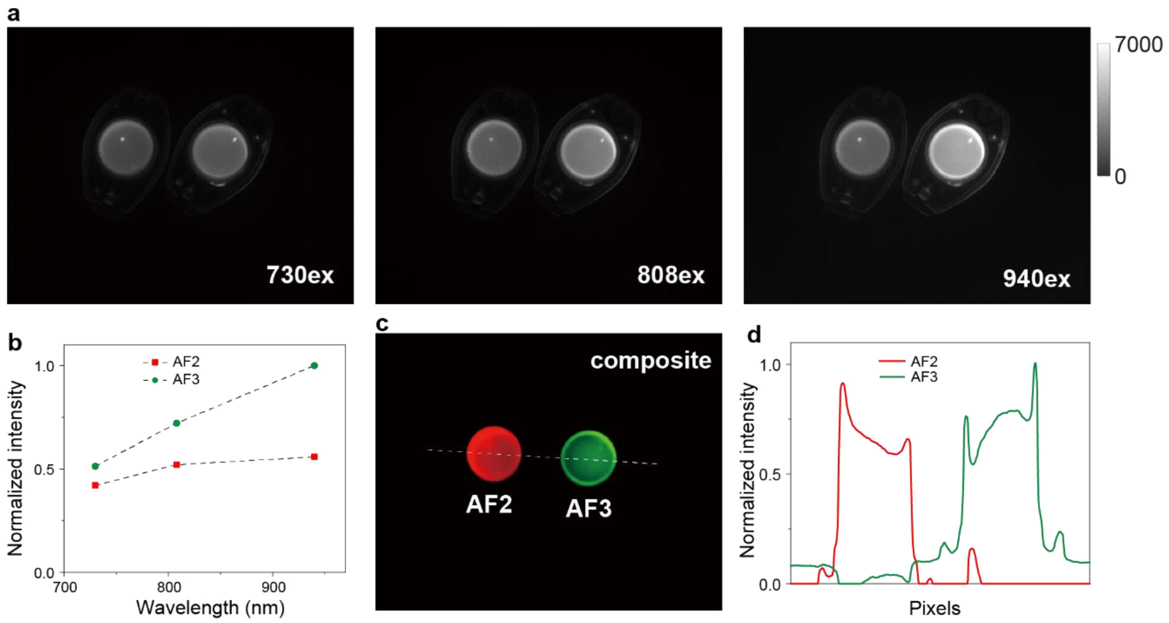

Supplementary Figure 28 | In-vitro fluorescence multispectral unmixing result of AF2 and AF3. **a,** Fluorescence image of AF2 and AF3 samples under 730 nm, 808 nm and 940 nm excitation; **b,** Acquired excitation spectra of AF2 and AF3; **c,** Multispectral unmixing result of AF2 and AF3; **d,** normalized signal intensity of AF2/AF3 channel on the dotted line region in **c**.

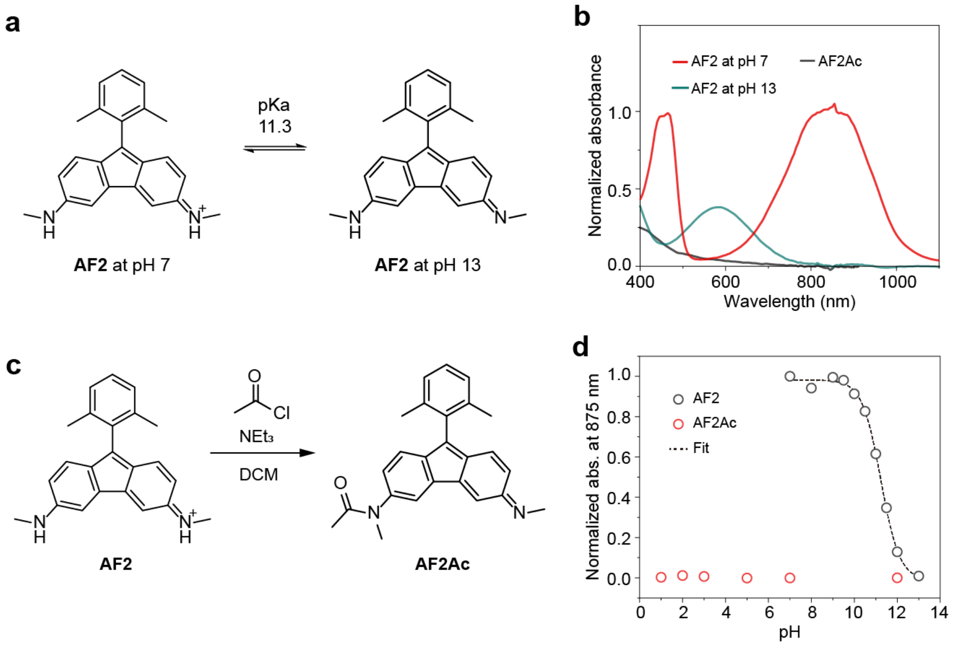

Supplementary Figure 29 | pH-response characteristics of AF2 and AF2Ac. **a,** Acid-base

equilibrium of AF2. **b,** Normalized absorption spectra of AF2 at pH 7 and 13, as well as AF2Ac at pH 7 (7:3 v/v PBS:MeCN). The 590 nm absorption peak of AF2 at pH 13 proved that the reaction product between AF2 and base was the deprotonated form shown in **a**, rather than an OH nucleophilic addition form at the 9- position, because this will result in the lose of conjugation and no visible absorbance will be observed. **c,** Chemical structure and synthetic route of AF2Ac. **d,** Absorbance at 875 nm of AF2 and AF2Ac versus pH values. Boltzmann curve fitting (dotted line) gave the pKa value of ~11.3.

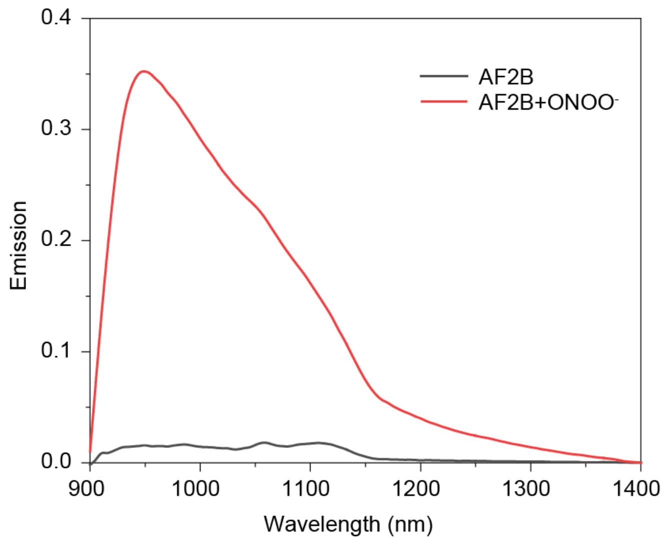

Supplementary Figure 30 | Absorption spectra of AF2B (50 μM) after incubation with $ONOO^-$ (25 μM).

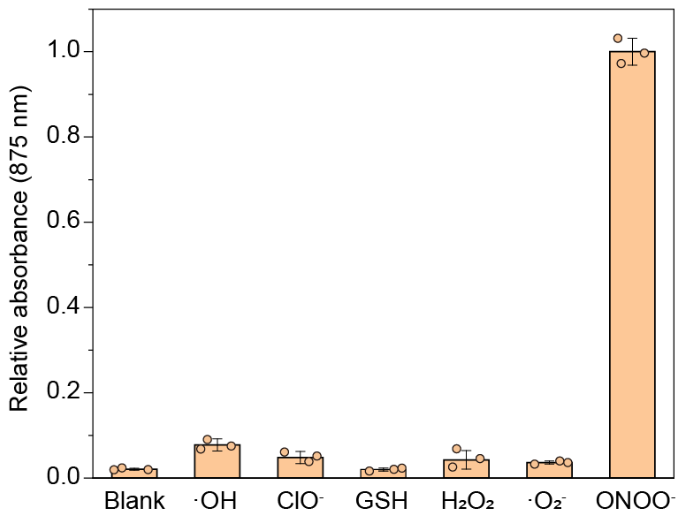

Supplementary Figure 31 | Selectivity of AF2B over ClO^- (50 eq.), H_2O_2 (50 eq.), •OH (20 eq.),

•O_2^- (50 eq.), GSH (50 eq.) and ONOO$^-$ (0.5 eq.). The bars represent mean ± s.d. derived from n = 3 independent groups.

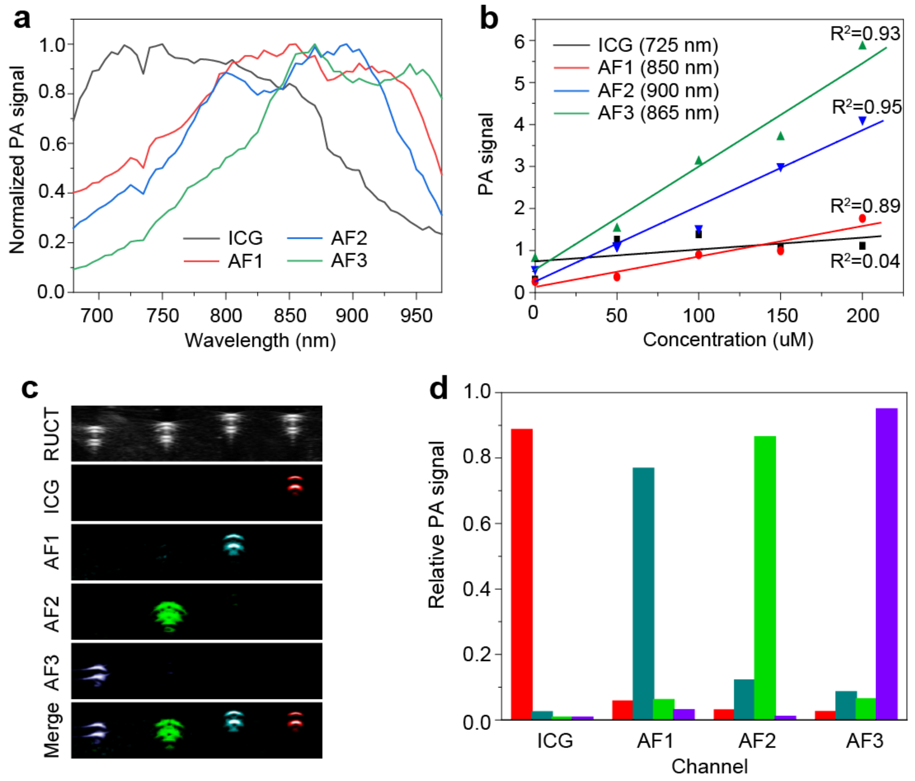

Supplementary Figure 32 | in-vitro photoacoustic (PA) spectra and multispectral unmixing of ICG, AF1, AF2, and AF3. a, Normalized PA spectra of ICG, AF1, AF2, and AF3 (200 μM, 1×PBS solution). **b,** Linear fit results of PA signal of ICG, AF1, AF2, and AF3 at certain wavelength. **c-d,** Multispectral PA unmixing results of ICG, AF1, AF2, and AF3.

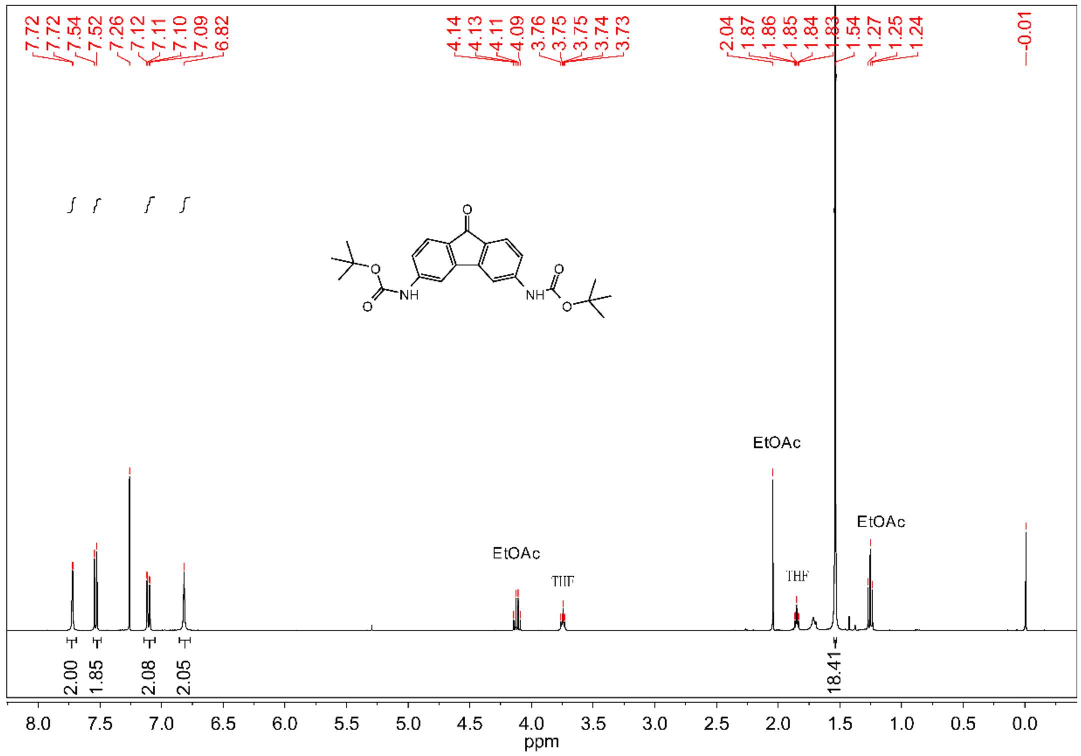

Supplementary Figure 33 | ^{1}H-NMR of 1a.

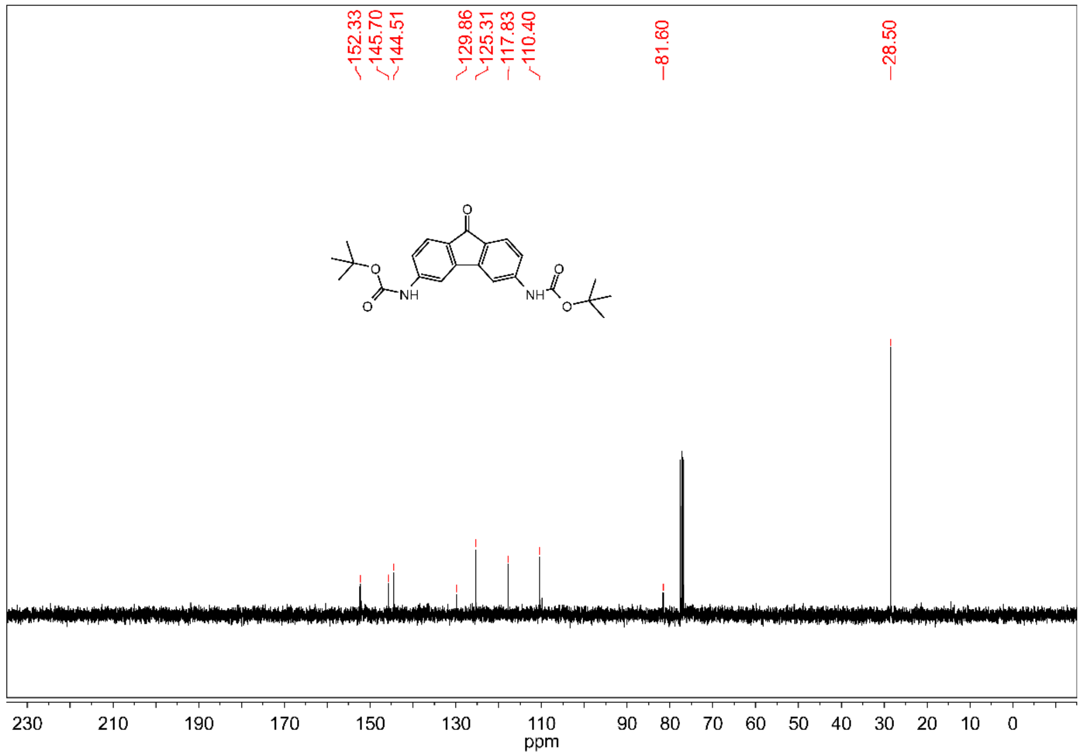

Supplementary Figure 34 | ^{13}C-NMR of 1a.

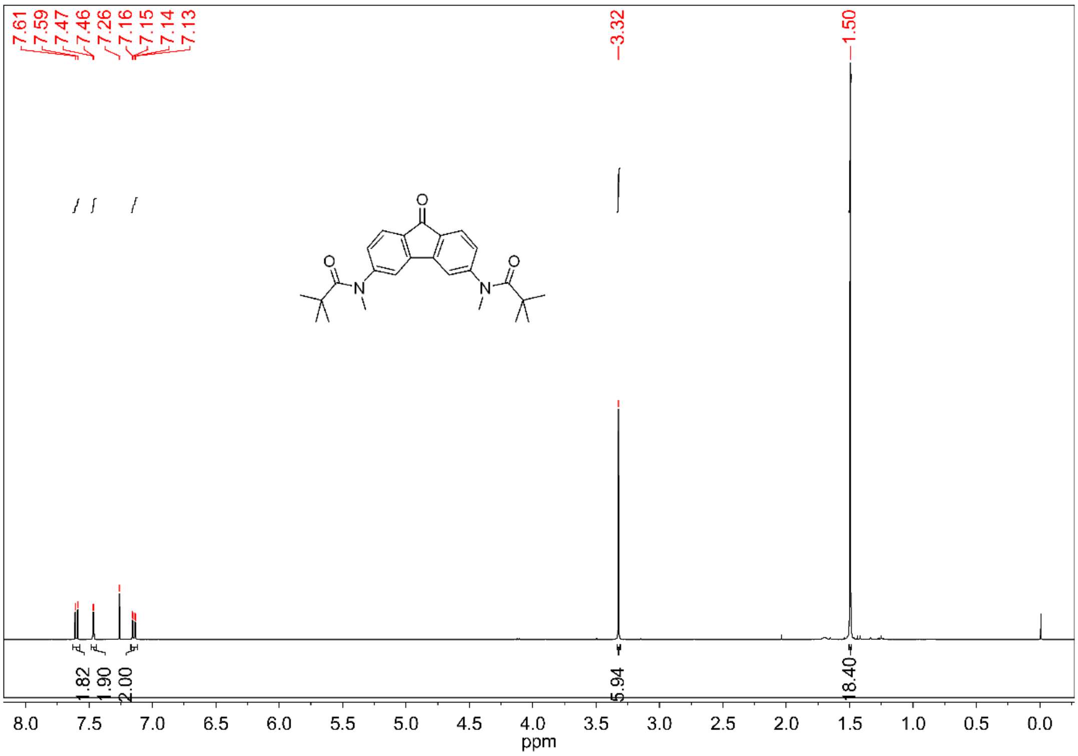

Supplementary Figure 35 | ^{1}H-NMR of 1b.

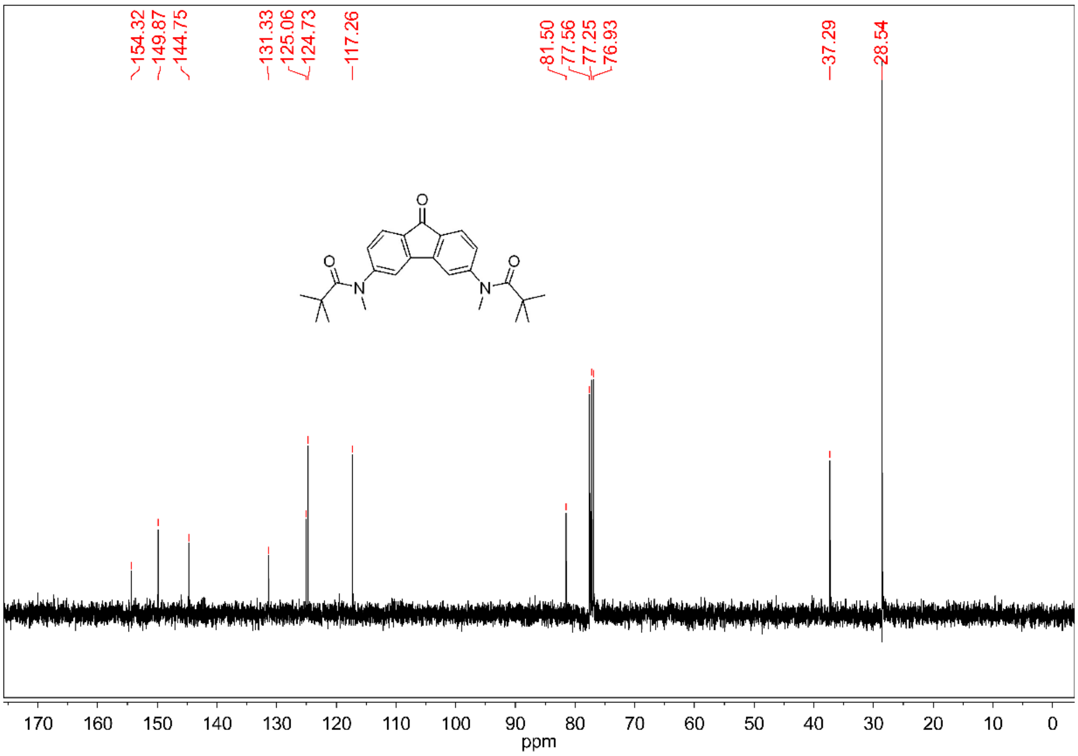

Supplementary Figure 36 | ^{13}C-NMR of 1b.

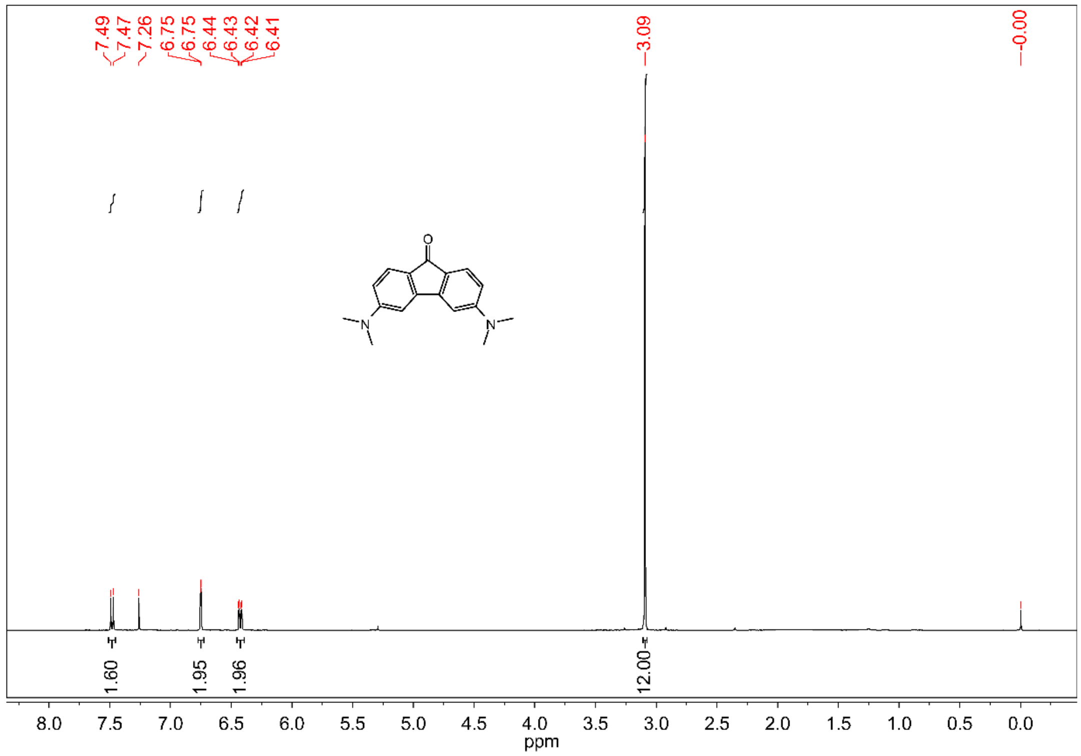

Supplementary Figure 37 | ^{1}H-NMR of 1c.

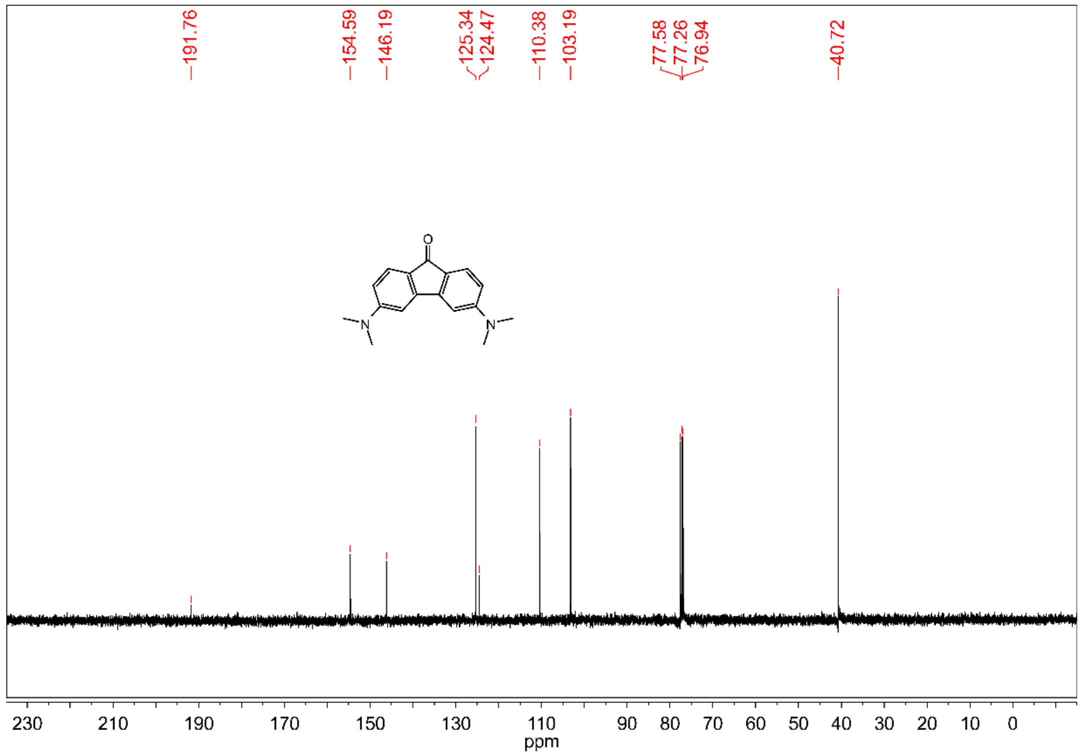

Supplementary Figure 38 | ^{13}C-NMR of 1c.

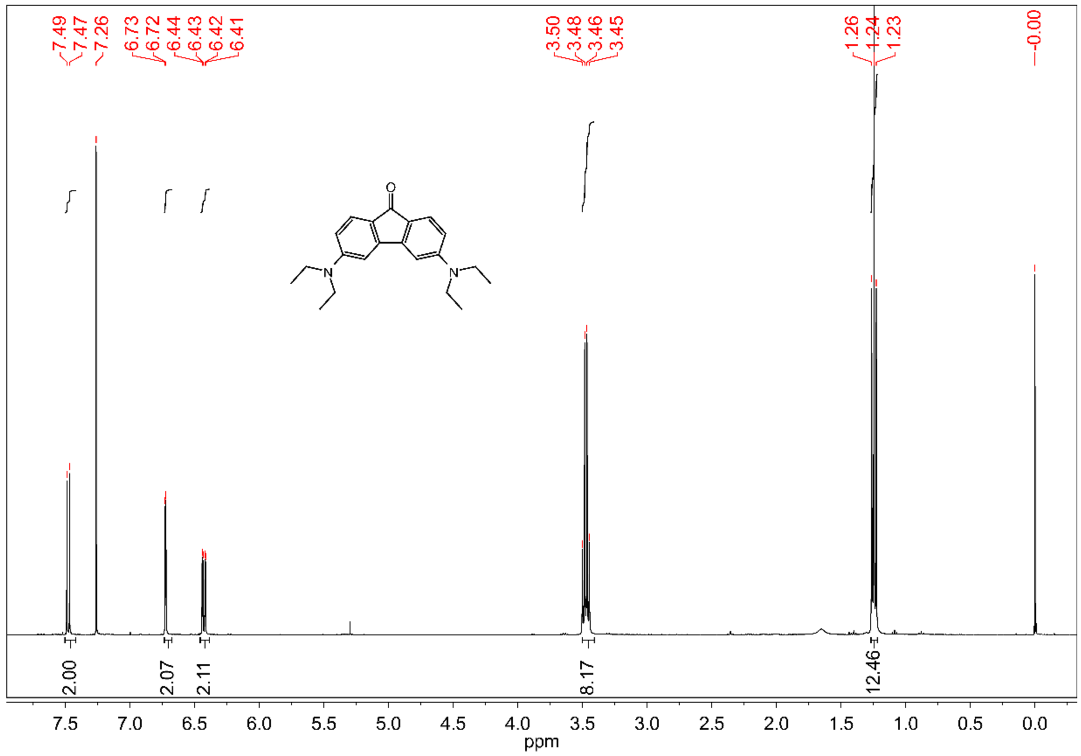

Supplementary Figure 39 | ^{1}H-NMR of 1d.

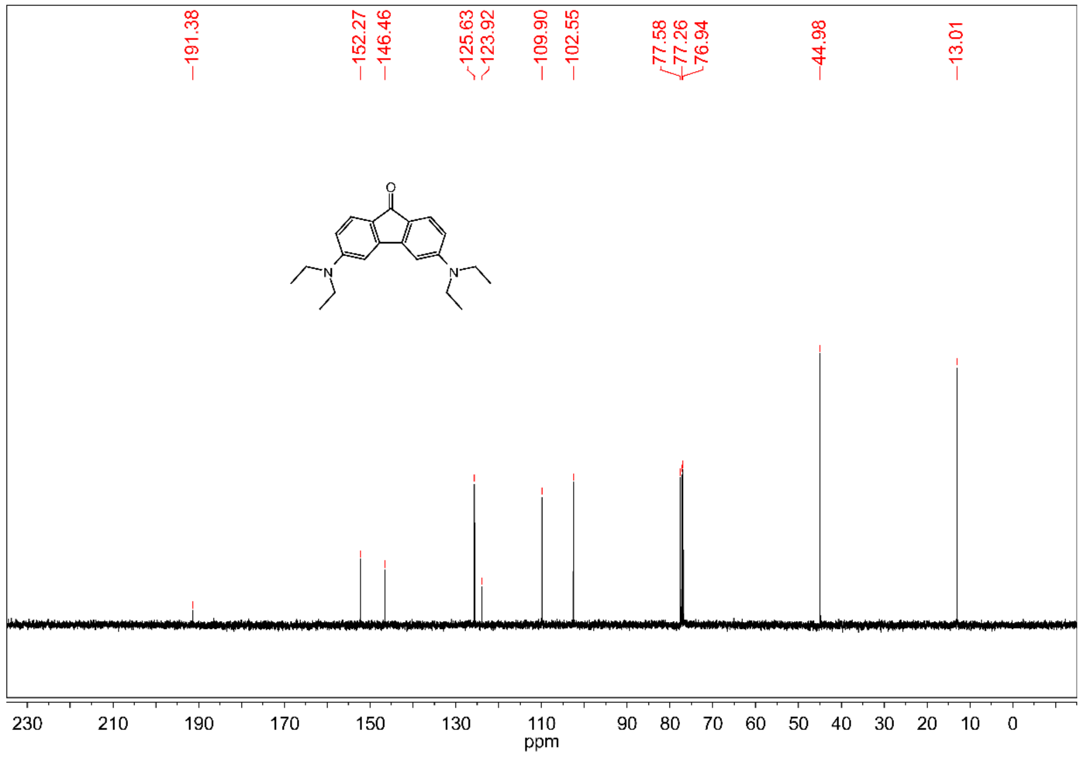

Supplementary Figure 40 | ^{13}C-NMR of 1d.

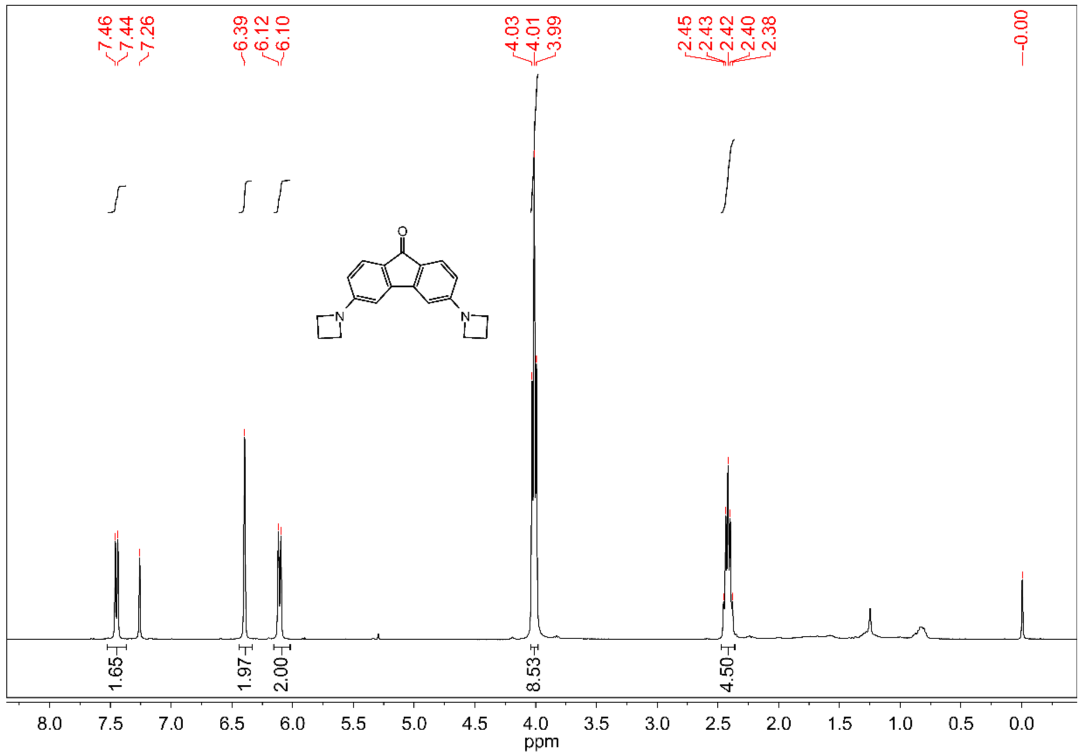

Supplementary Figure 41 | ^{1}H-NMR of 1e.

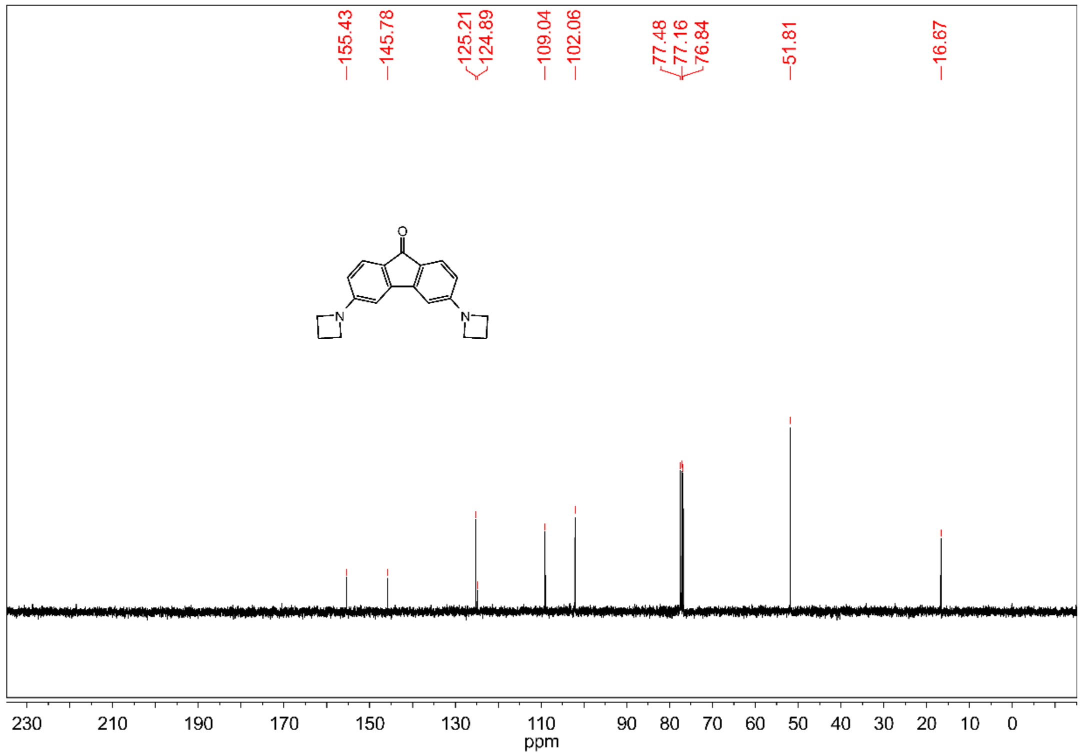

Supplementary Figure 42 | ^{13}C-NMR of 1e.

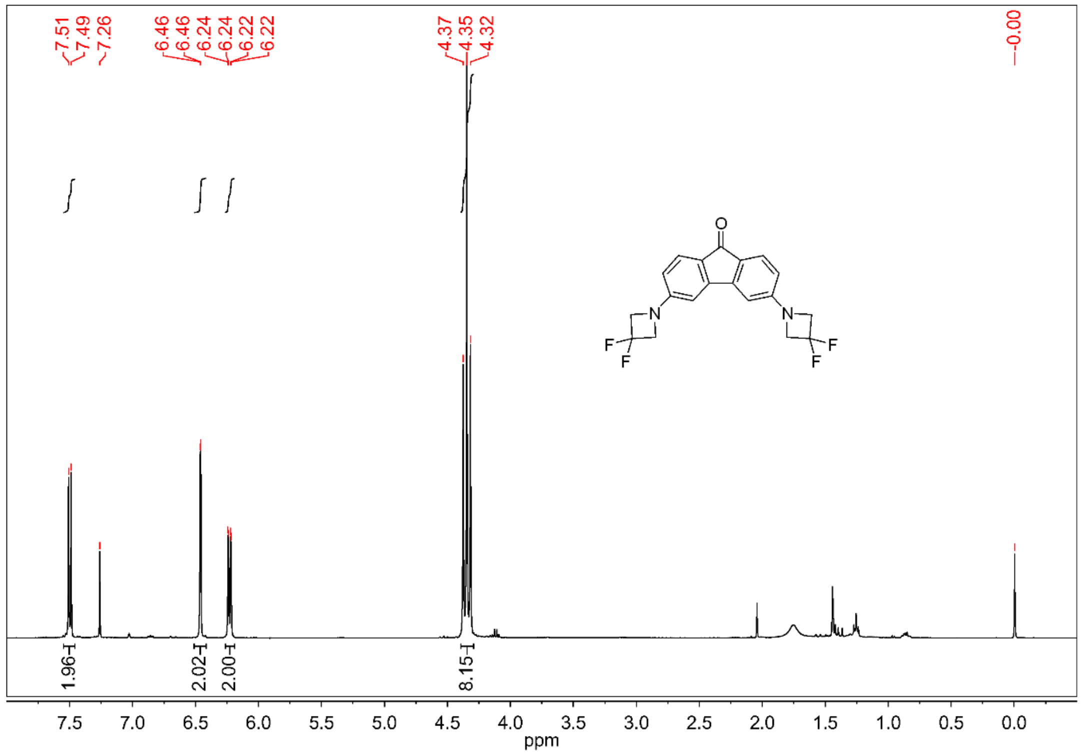

Supplementary Figure 43 | ^{1}H-NMR of 1f.

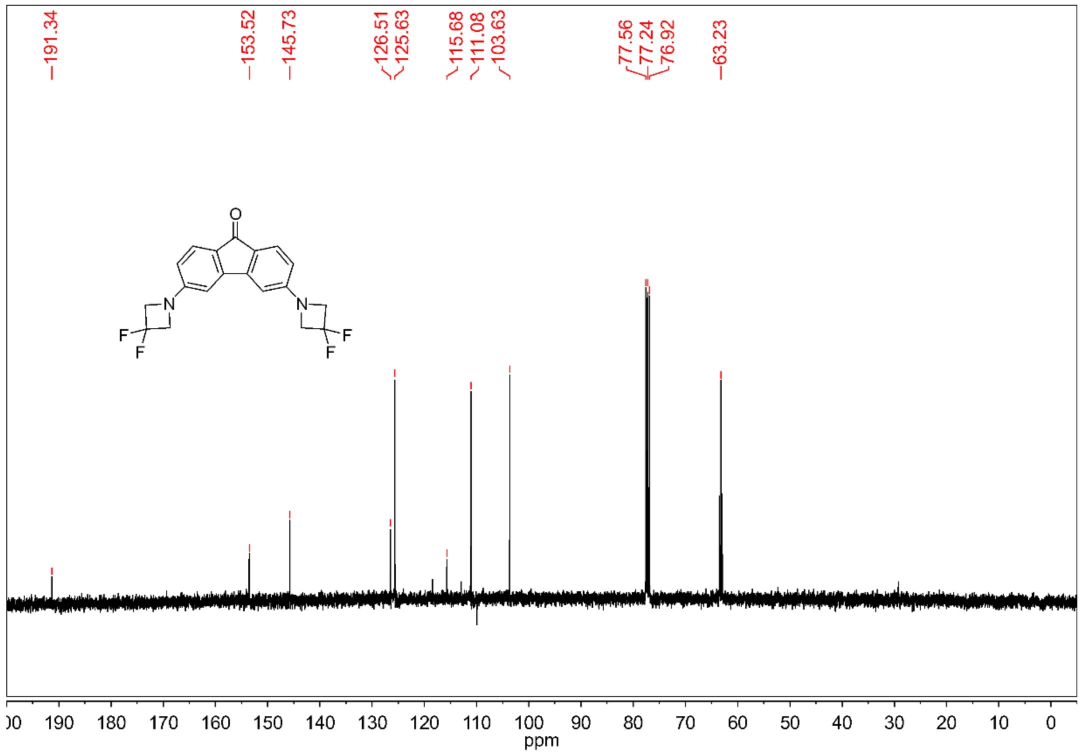

Supplementary Figure 44 | ^{13}C-NMR of 1f.

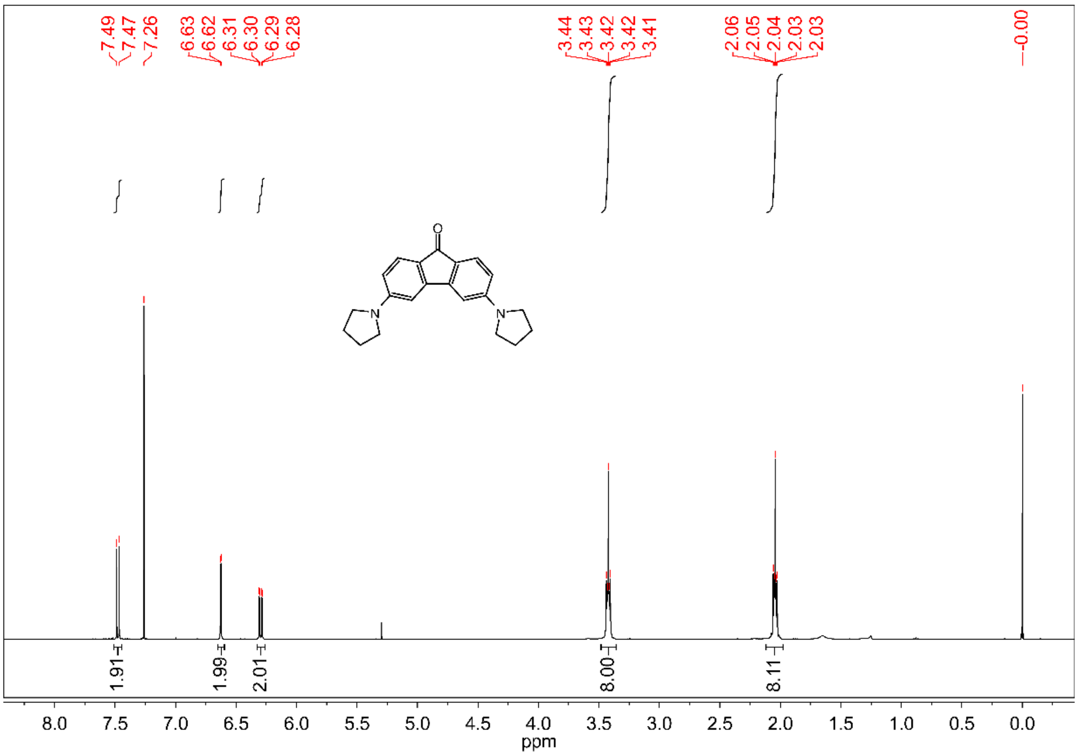

Supplementary Figure 45 | ^{1}H-NMR of 1g.

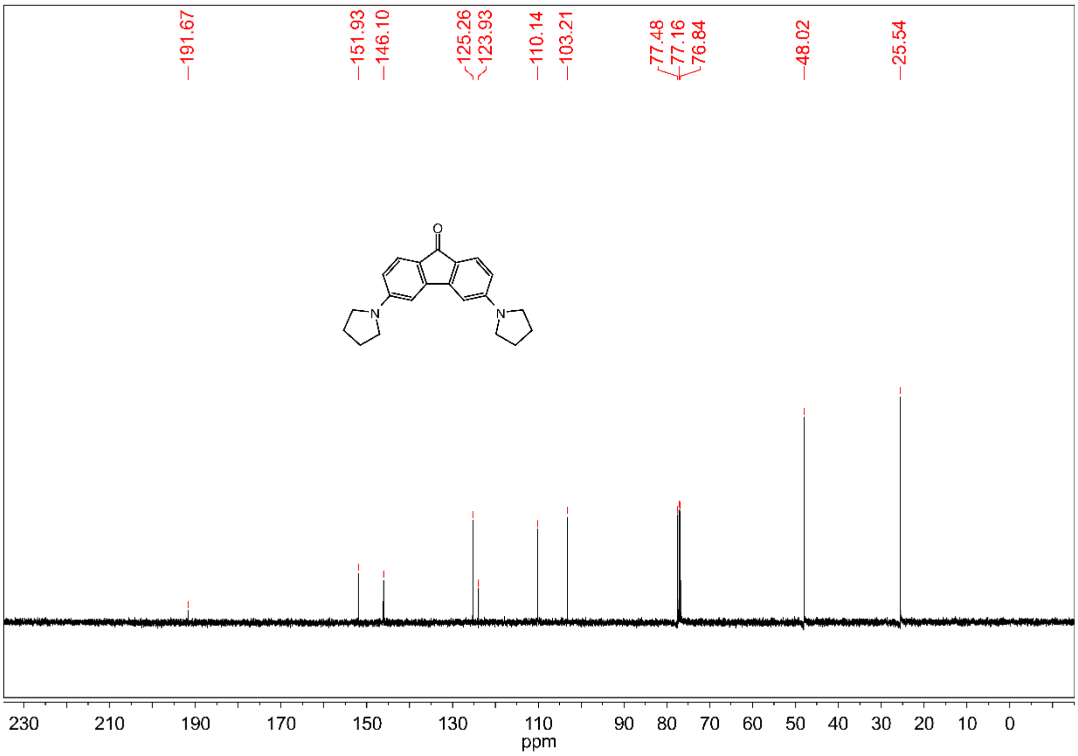

Supplementary Figure 46 | ^{13}C-NMR of 1g.

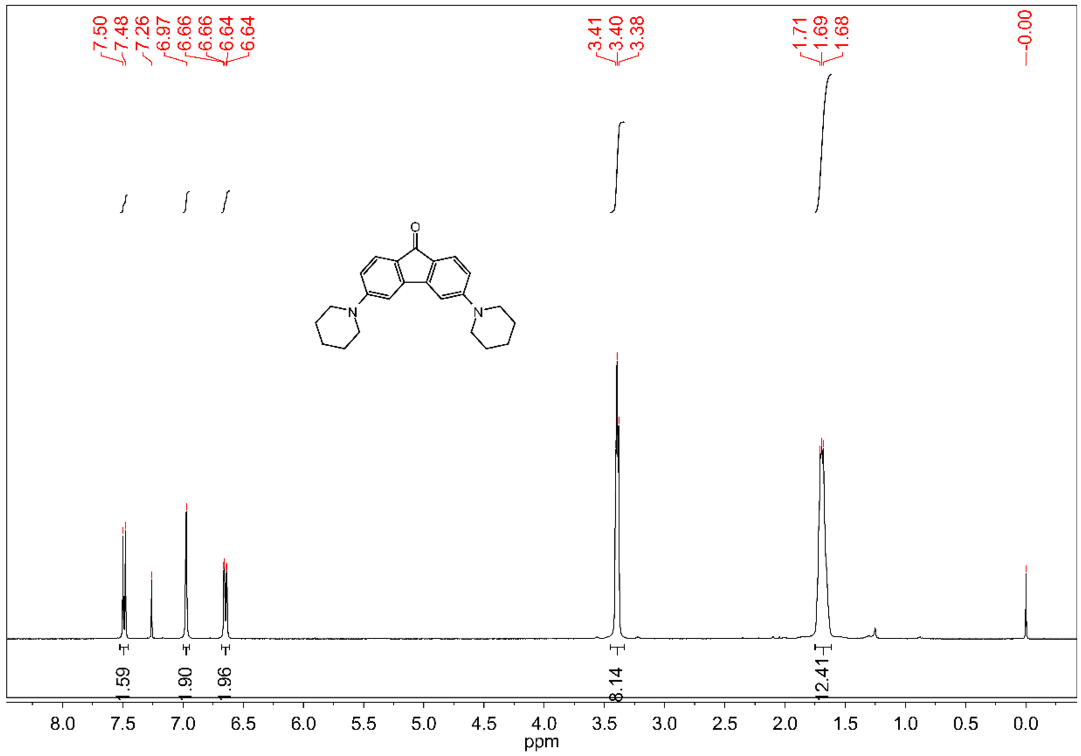

Supplementary Figure 47 | ^{1}H-NMR of 1h.

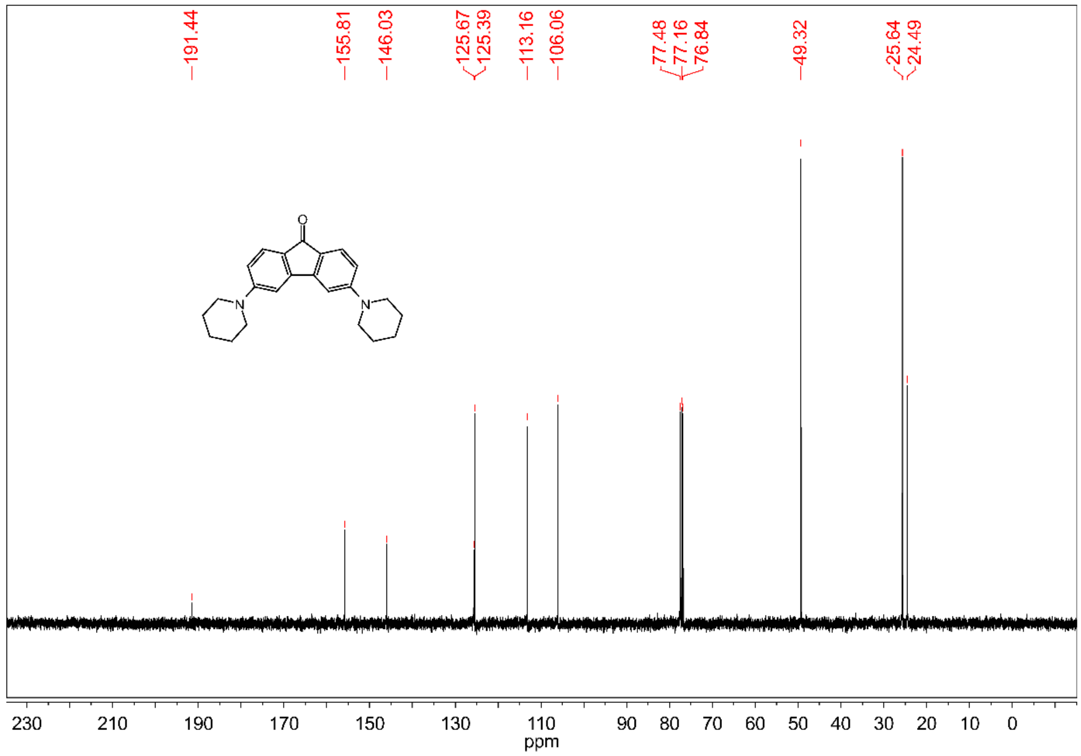

Supplementary Figure 48 | ^{13}C-NMR of 1h.

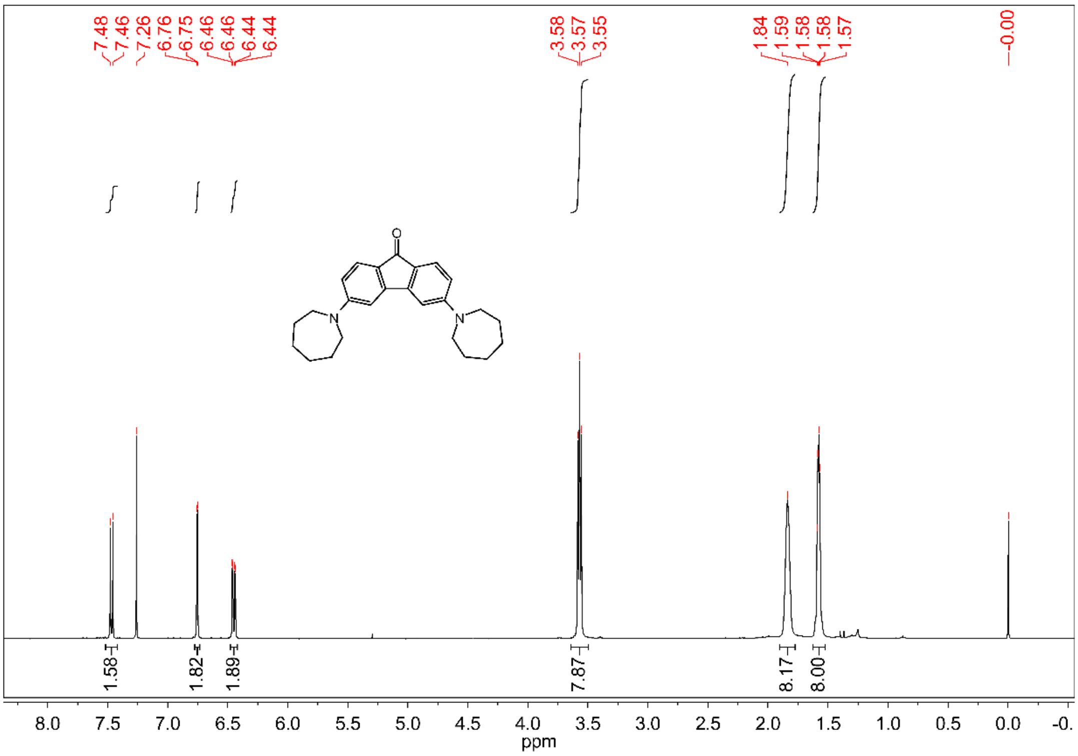

Supplementary Figure 49 | ^{1}H-NMR of 1i.

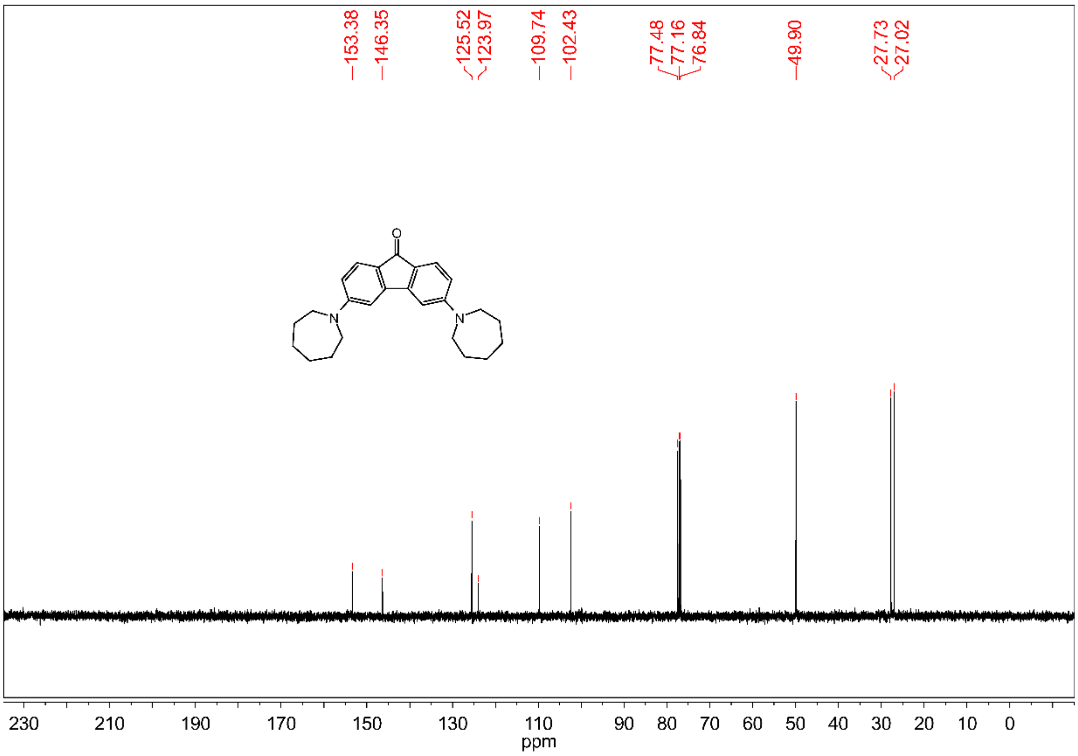

Supplementary Figure 50 | ^{13}C-NMR of 1i.

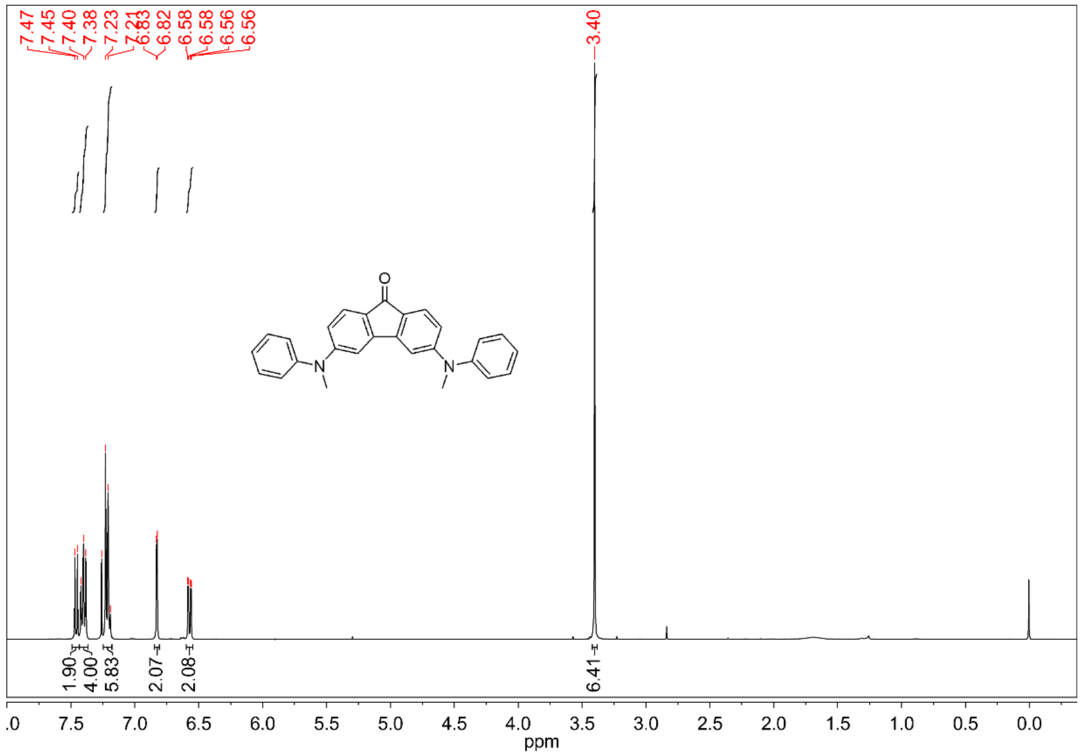

Supplementary Figure 51 | ^{1}H-NMR of 1j.

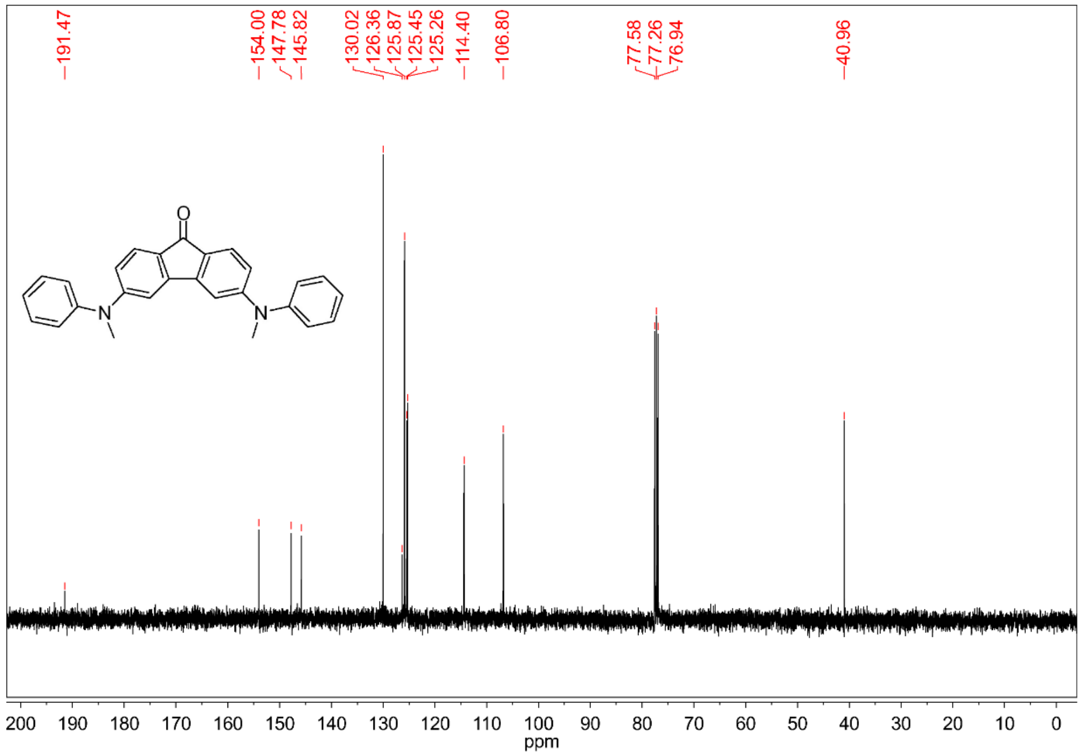

Supplementary Figure 52 | ^{13}C-NMR of 1j.

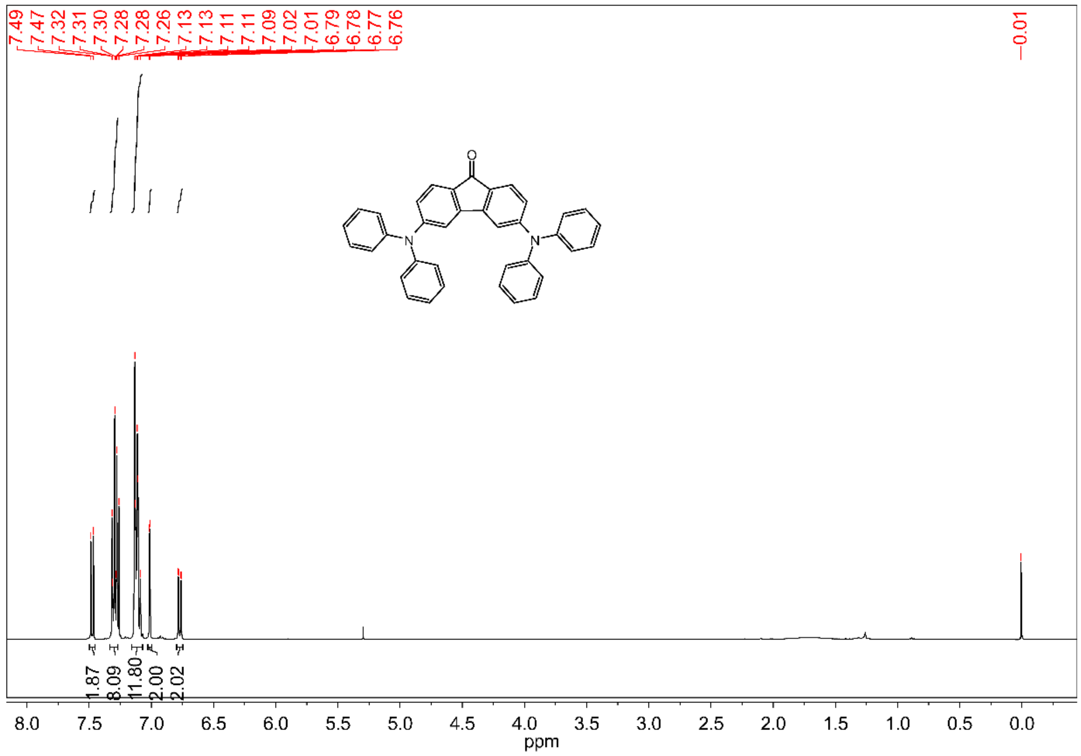

Supplementary Figure 53 | ^{1}H-NMR of 1k.

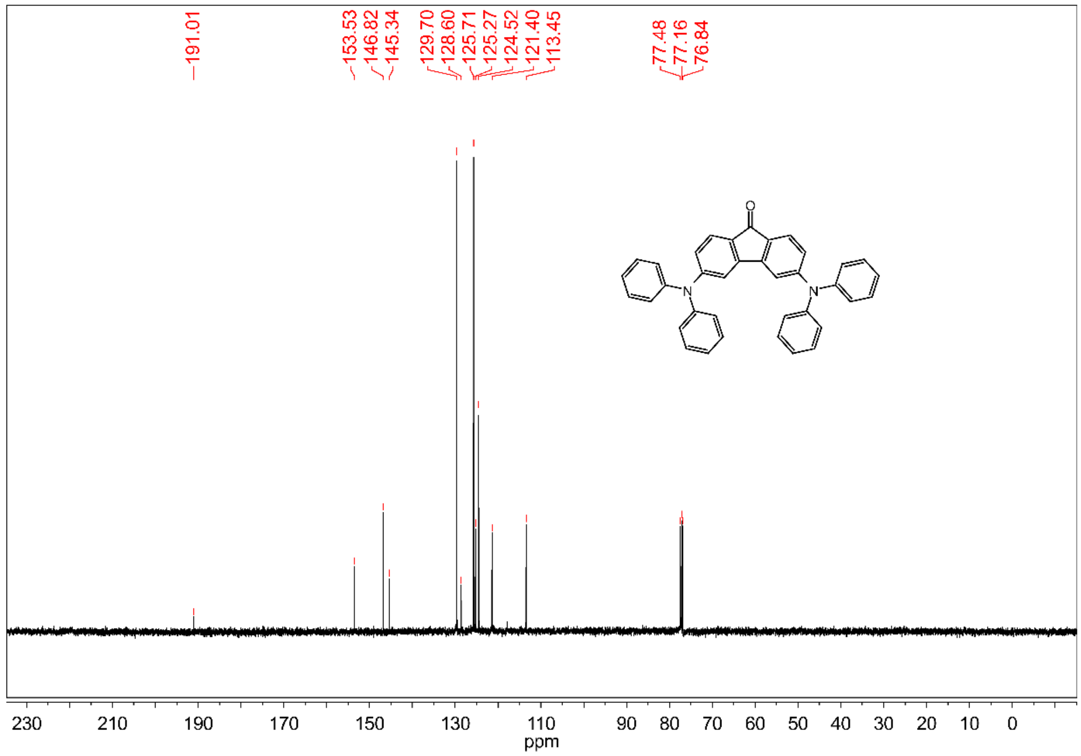

Supplementary Figure 54 | ^{13}C-NMR of 1k.

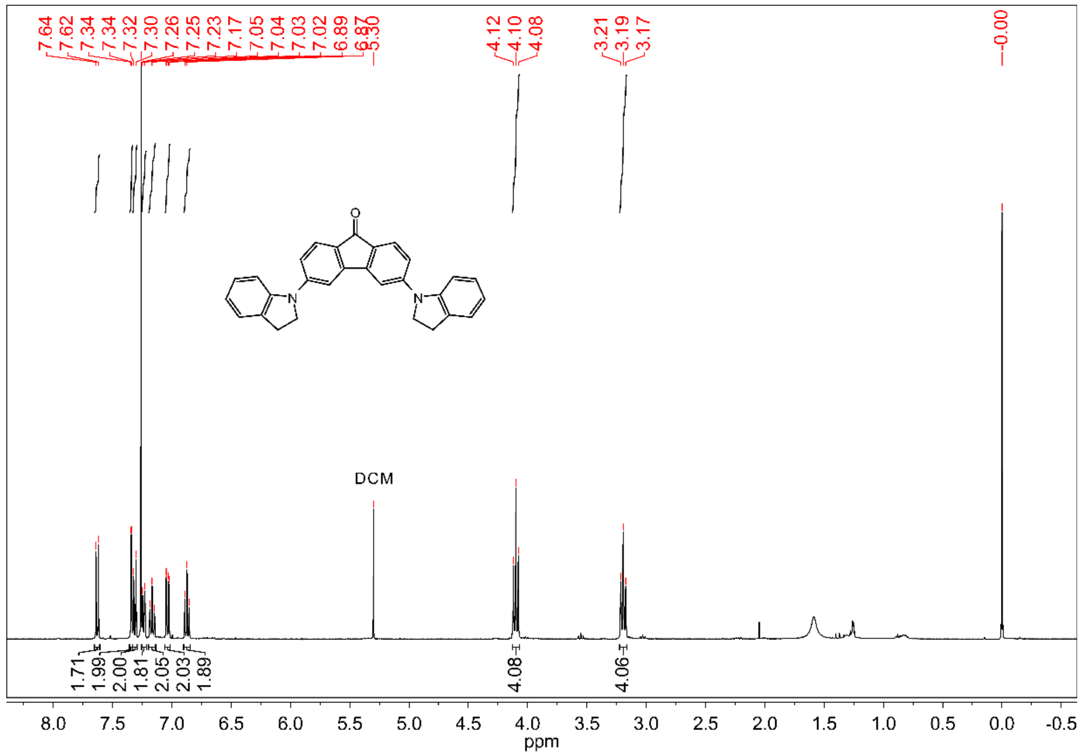

Supplementary Figure 55 | ^{1}H-NMR of 1m.

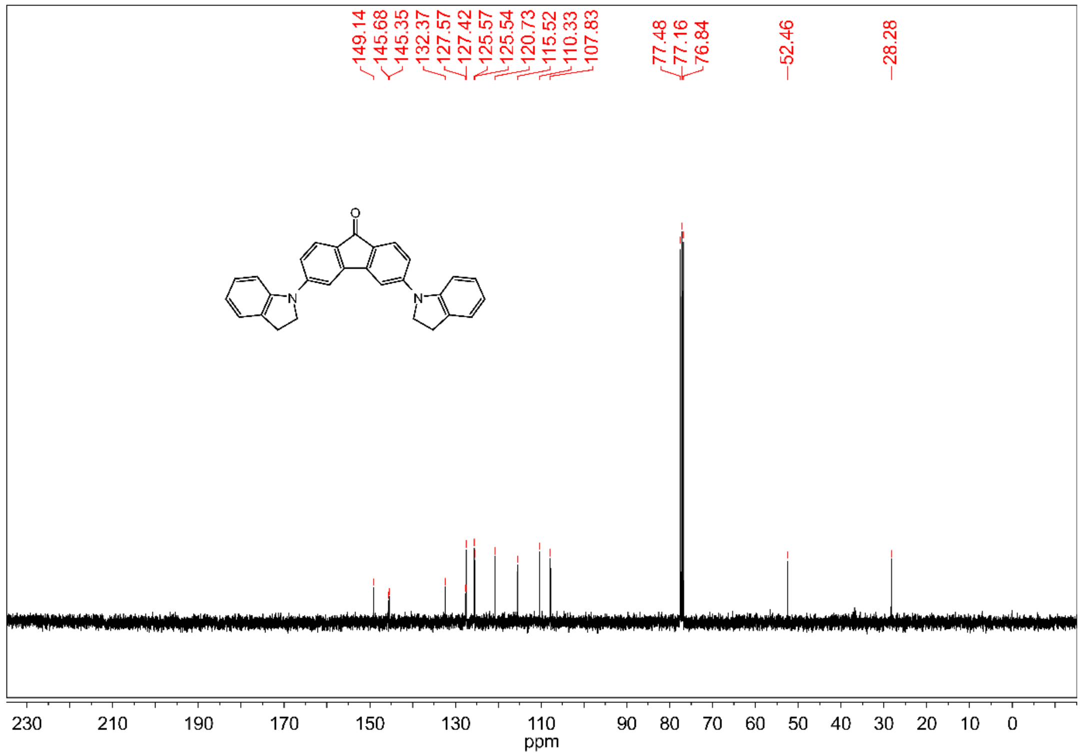

Supplementary Figure 56 | ^{13}C-NMR of 1m.

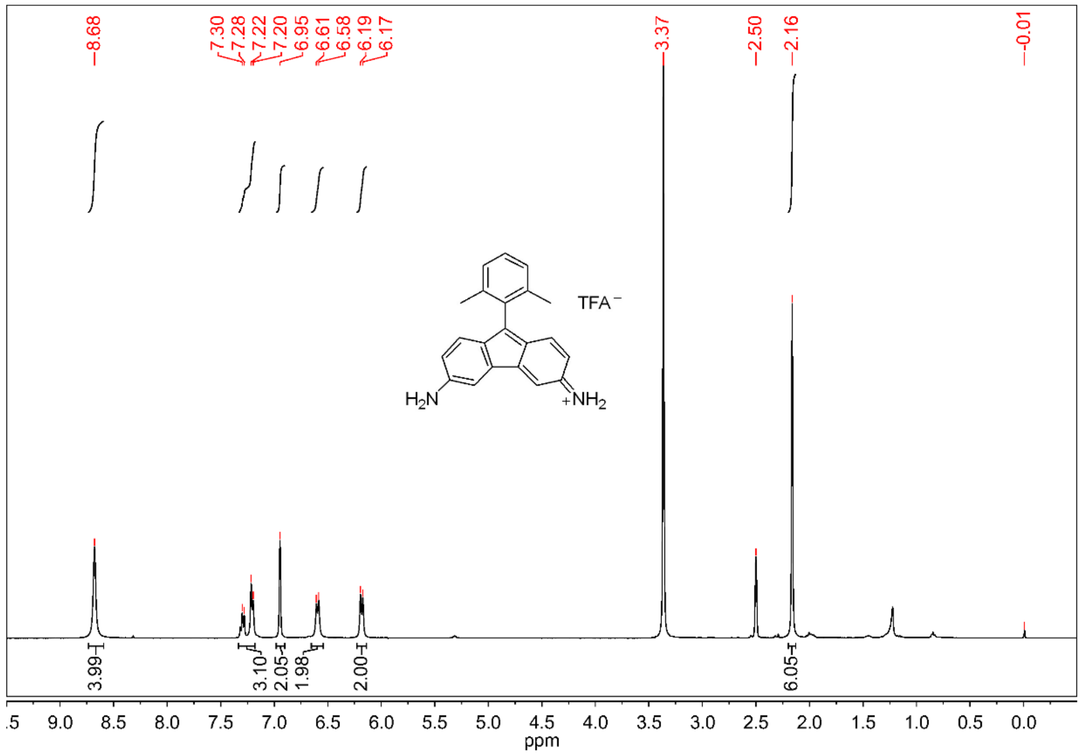

Supplementary Figure 57 | ^{1}H-NMR of AF2.

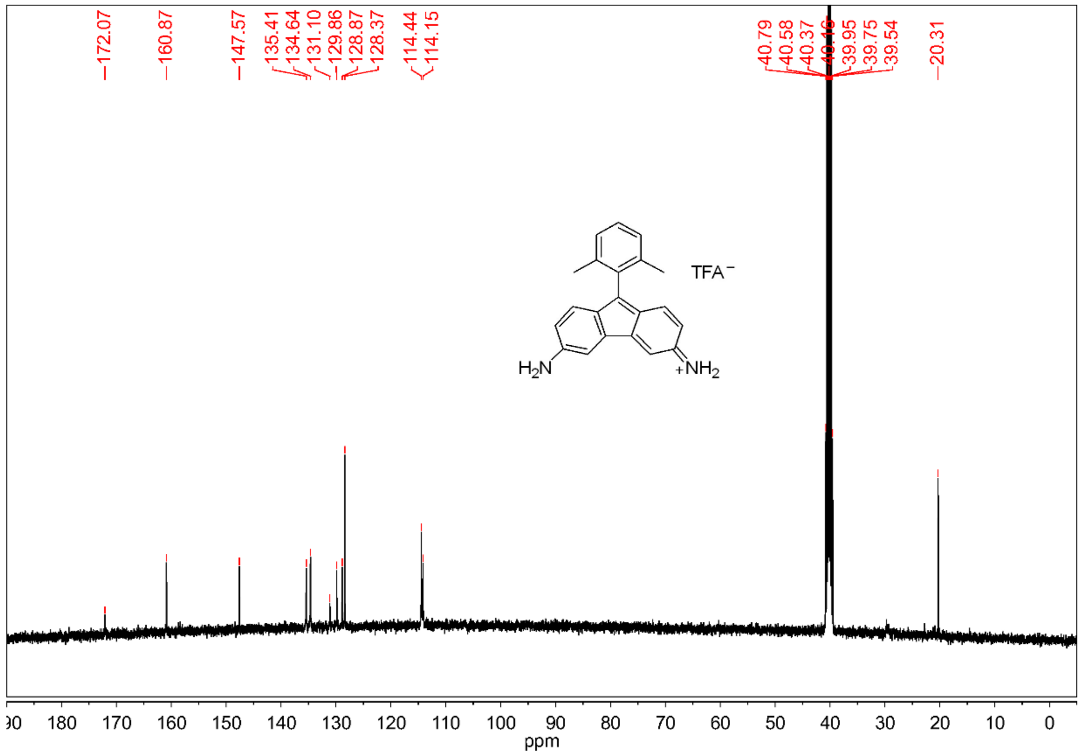

Supplementary Figure 58 | ^{13}C-NMR of AF2.

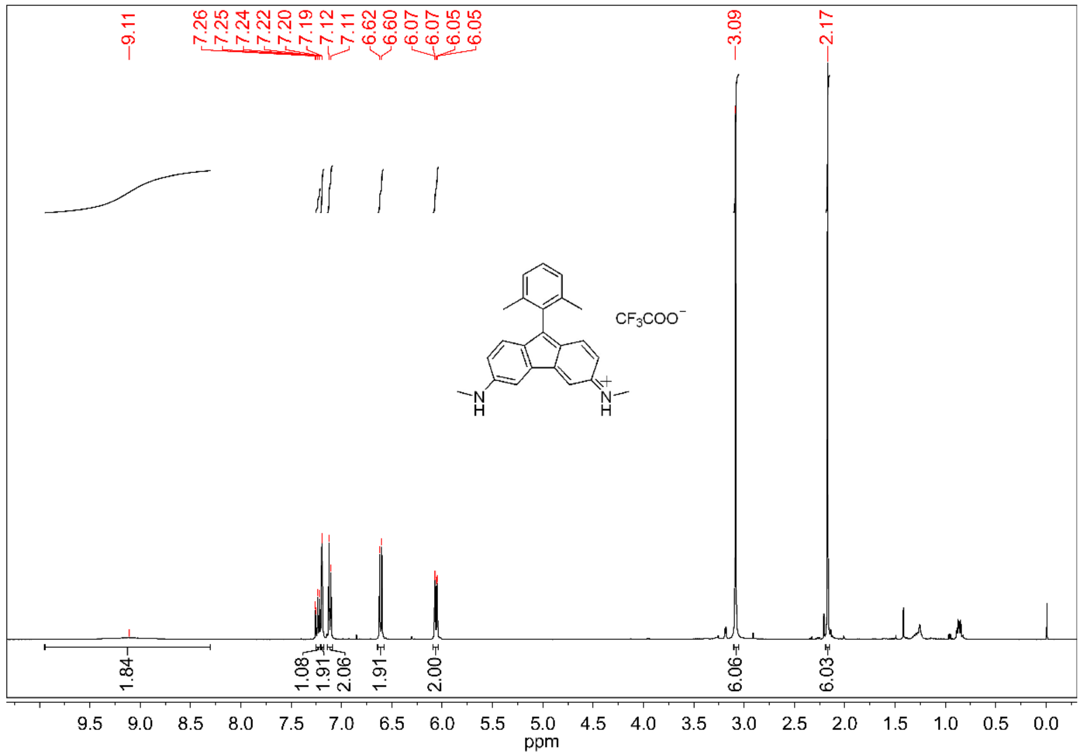

Supplementary Figure 59 | ^{1}H-NMR of AF2.

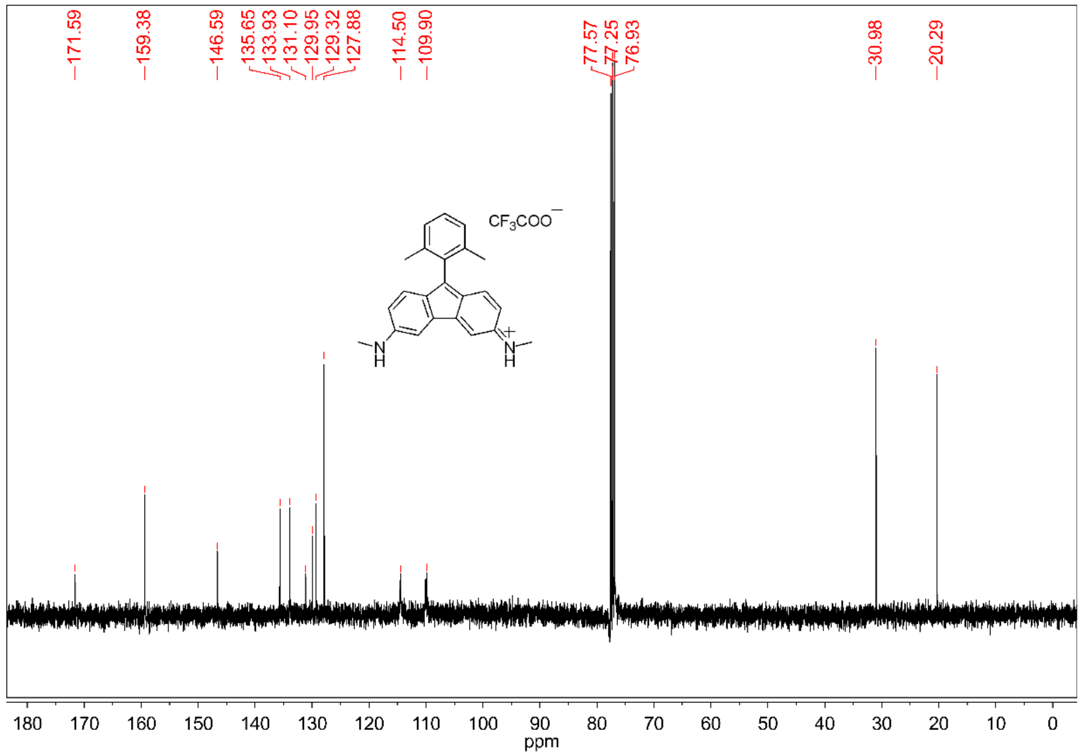

Supplementary Figure 60 | ^{13}C-NMR of AF2.

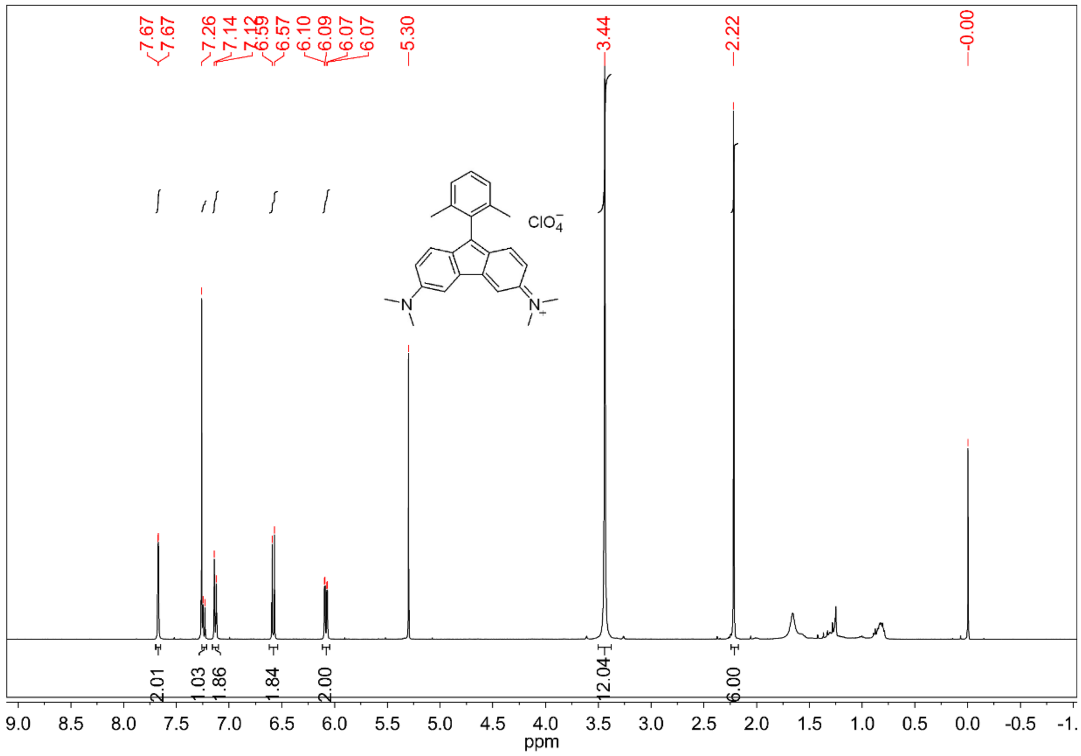

Supplementary Figure 61 | ^{1}H-NMR of AF3.

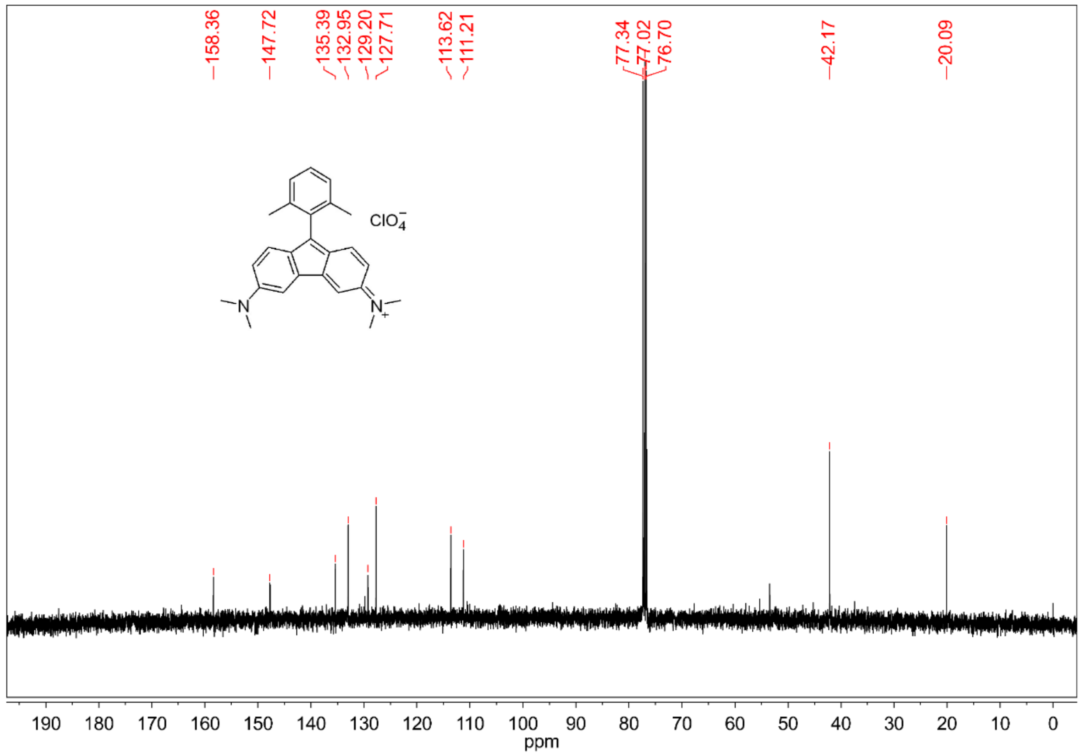

Supplementary Figure 62 | ^{13}C-NMR of AF3.

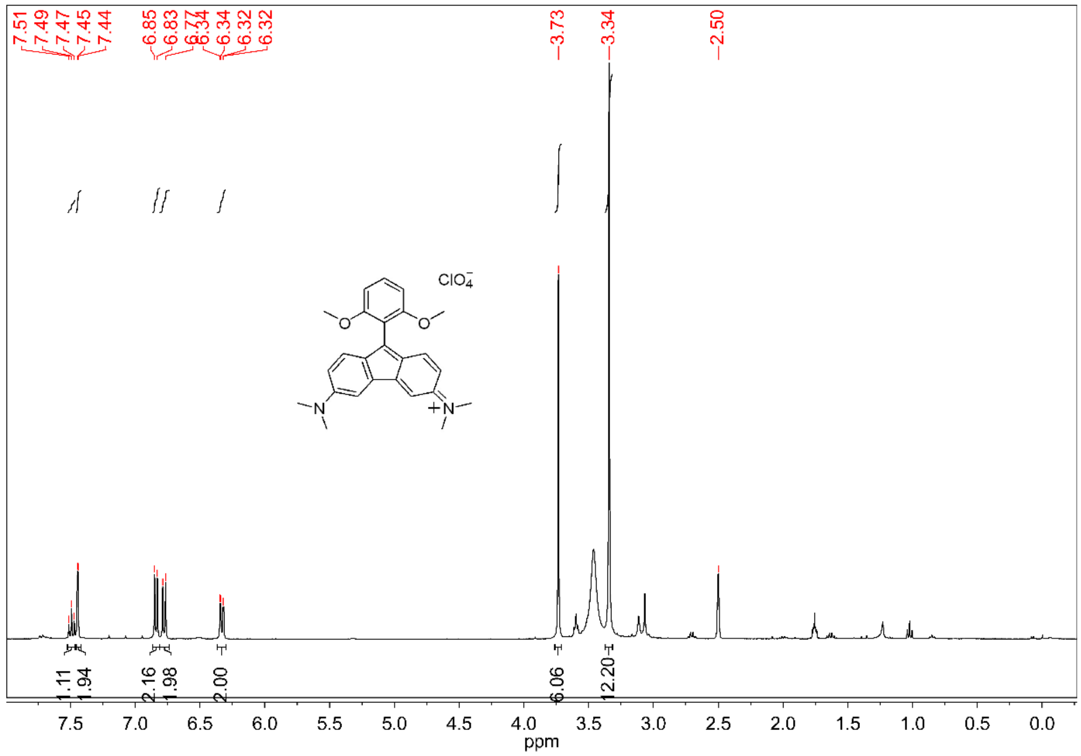

Supplementary Figure 63 | ^{1}H-NMR of AF4.

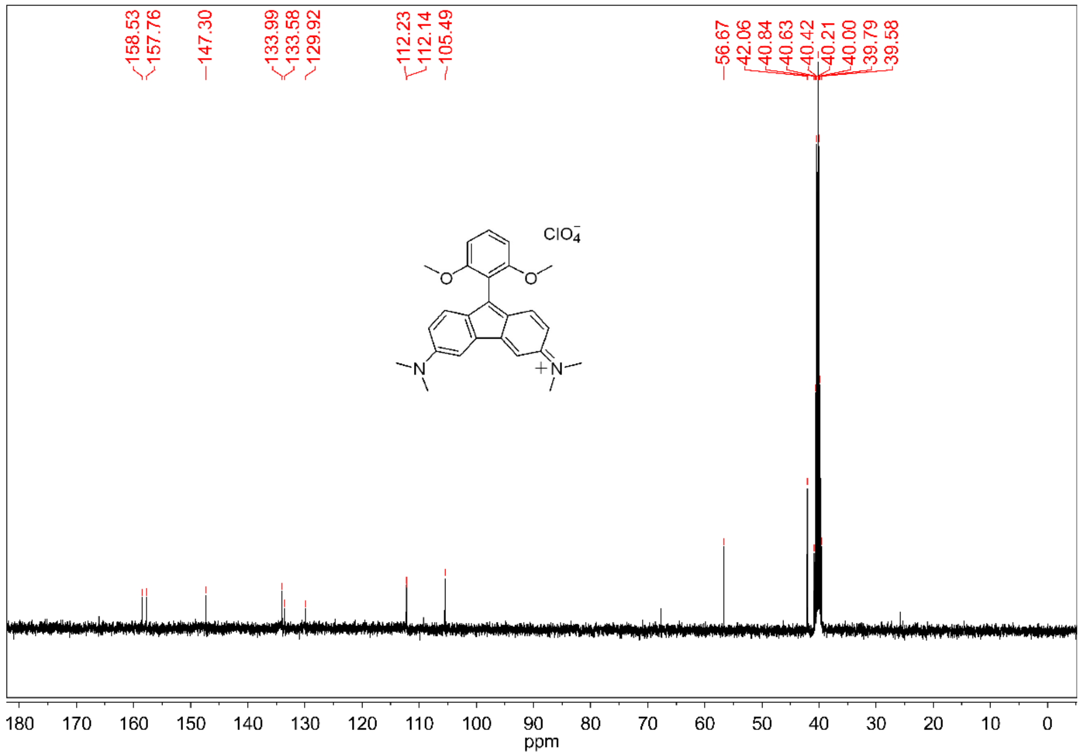

Supplementary Figure 64 | ^{13}C-NMR of AF4.

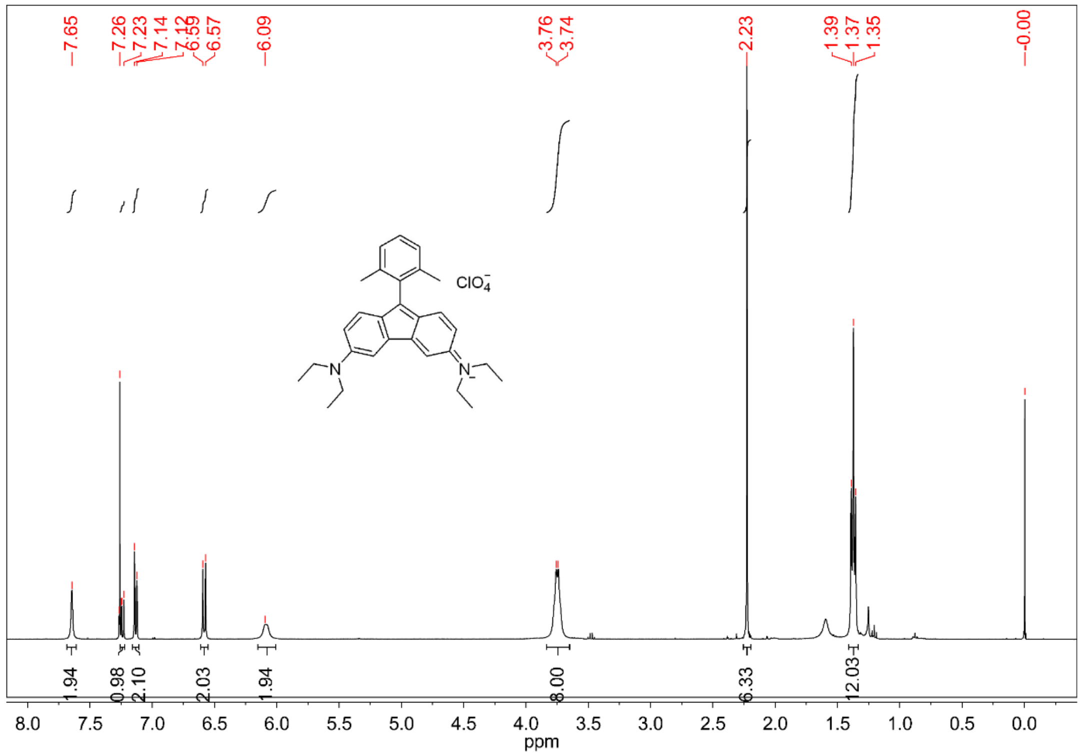

Supplementary Figure 65 | ^{1}H-NMR of AF5.

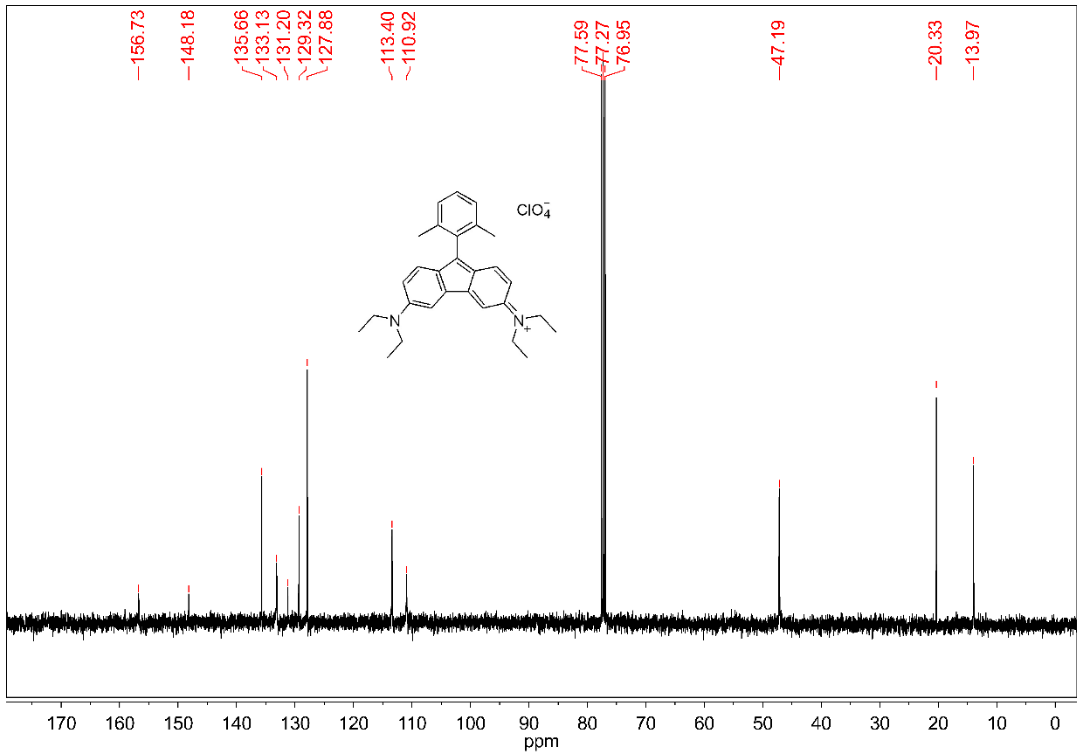

Supplementary Figure 66 | ^{13}C-NMR of AF5.

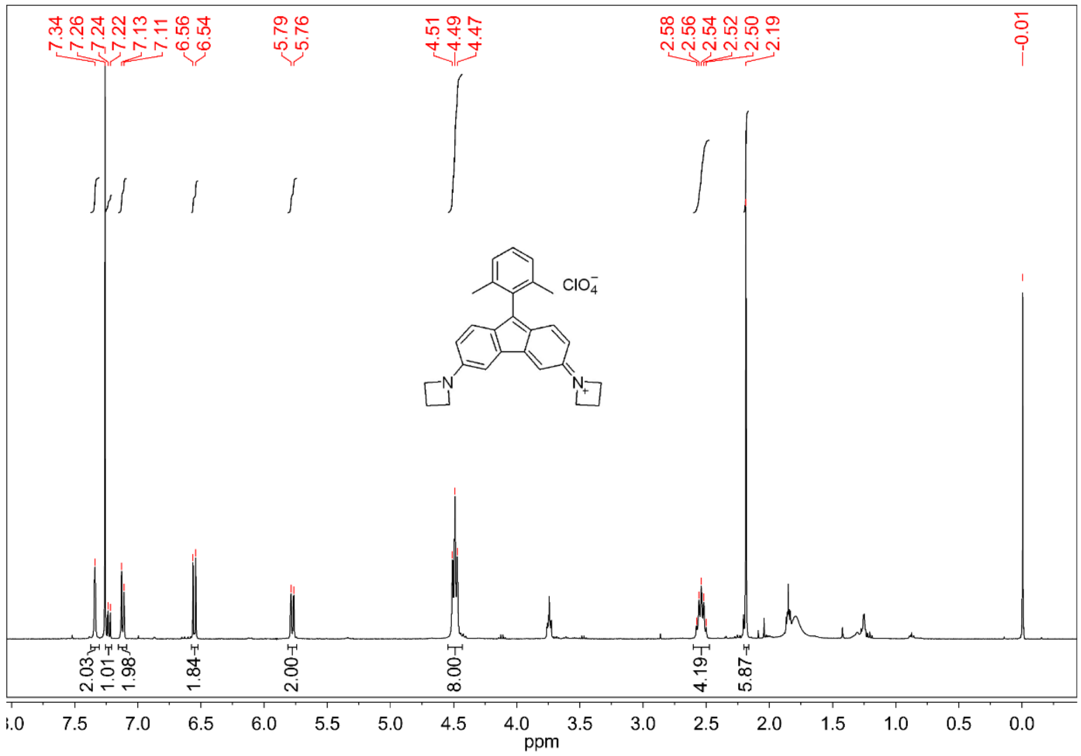

Supplementary Figure 67 | ^{1}H-NMR of AF6.

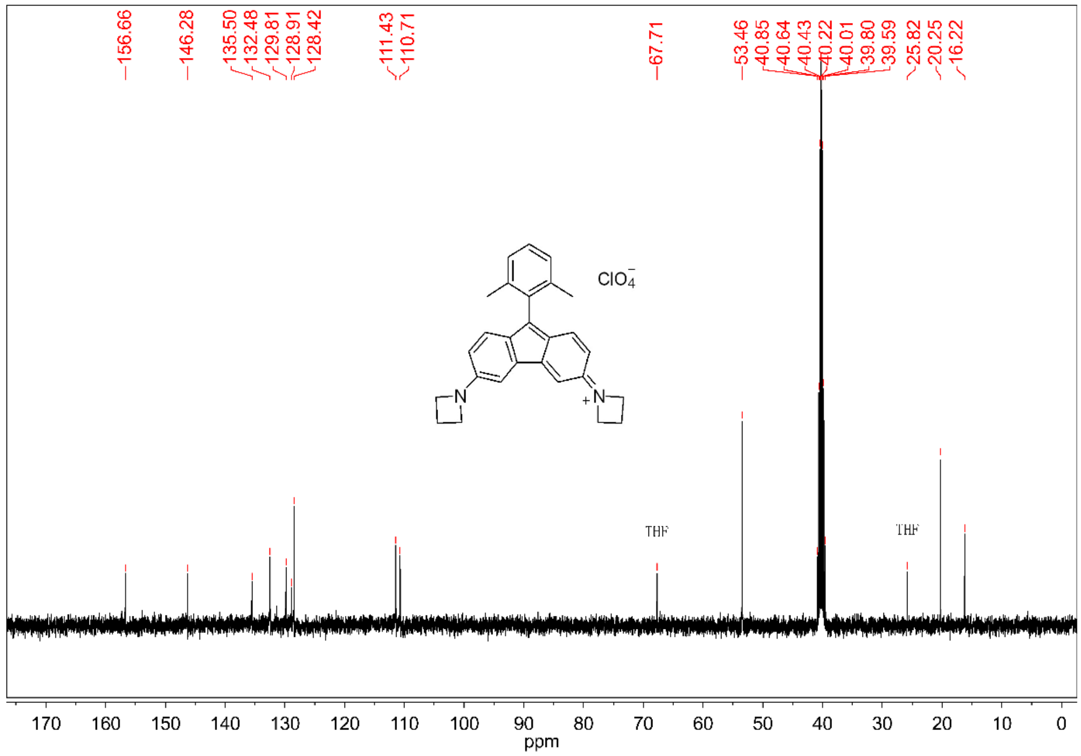

Supplementary Figure 68 | ^{13}C-NMR of AF6.

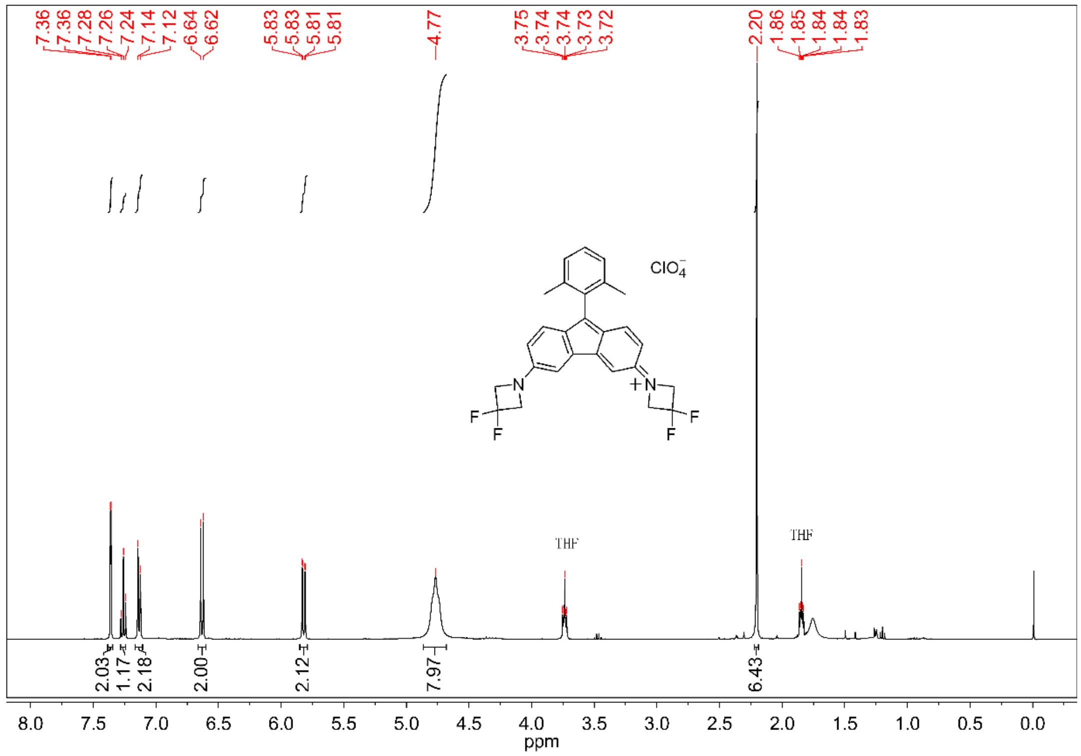

Supplementary Figure 69 | ^{1}H-NMR of AF7.

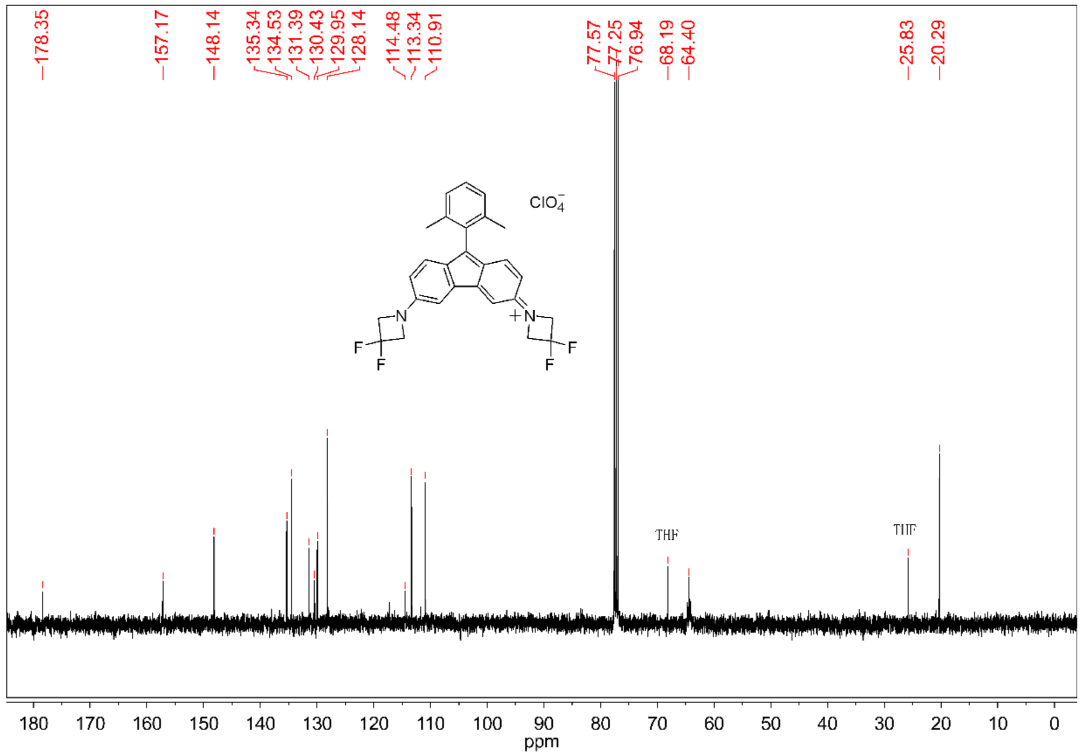

Supplementary Figure 70 | ^{13}C-NMR of AF7.

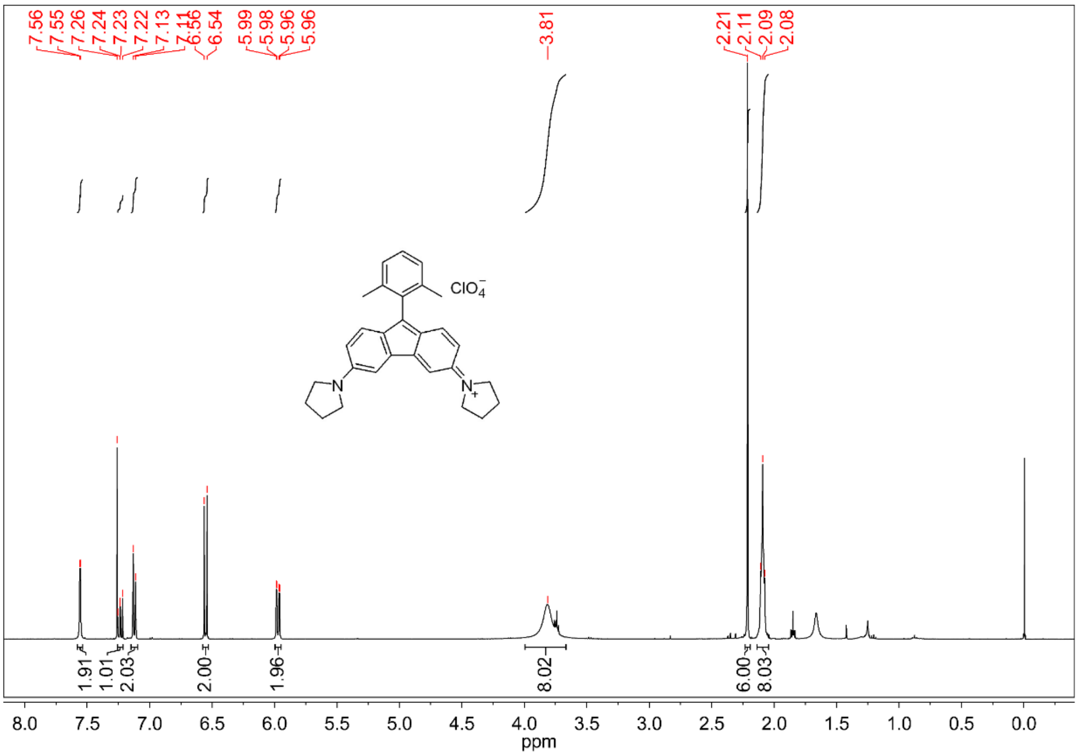

Supplementary Figure 71 | ^{1}H-NMR of AF8.

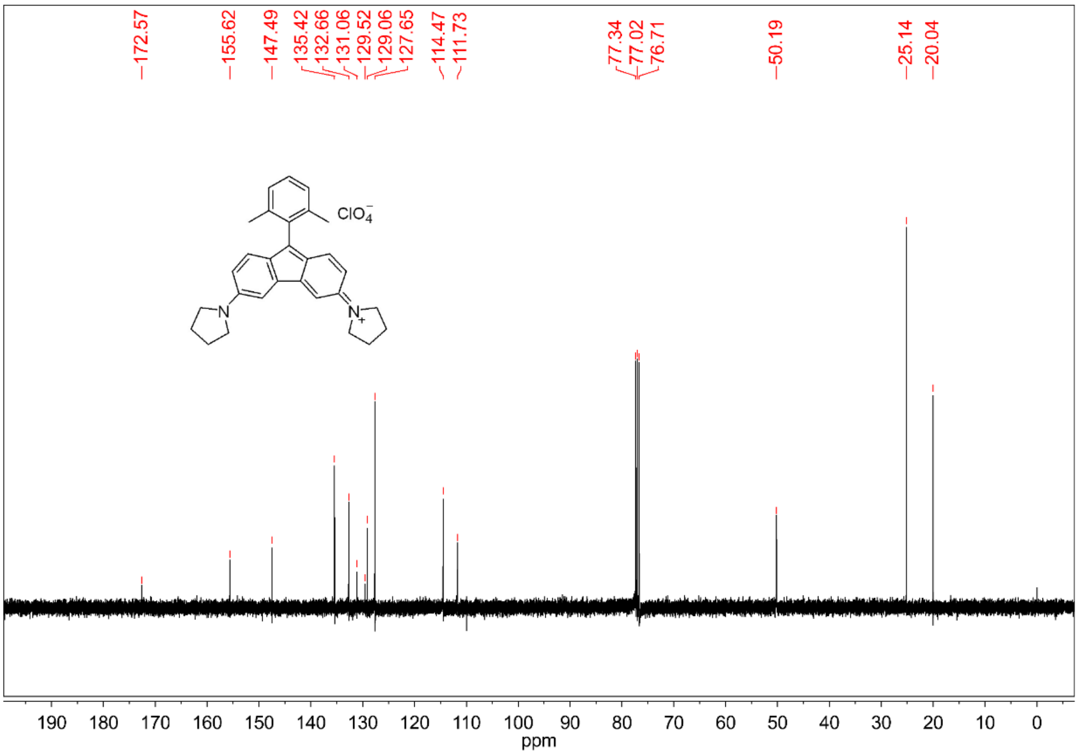

Supplementary Figure 72 | ^{13}C-NMR of AF8.

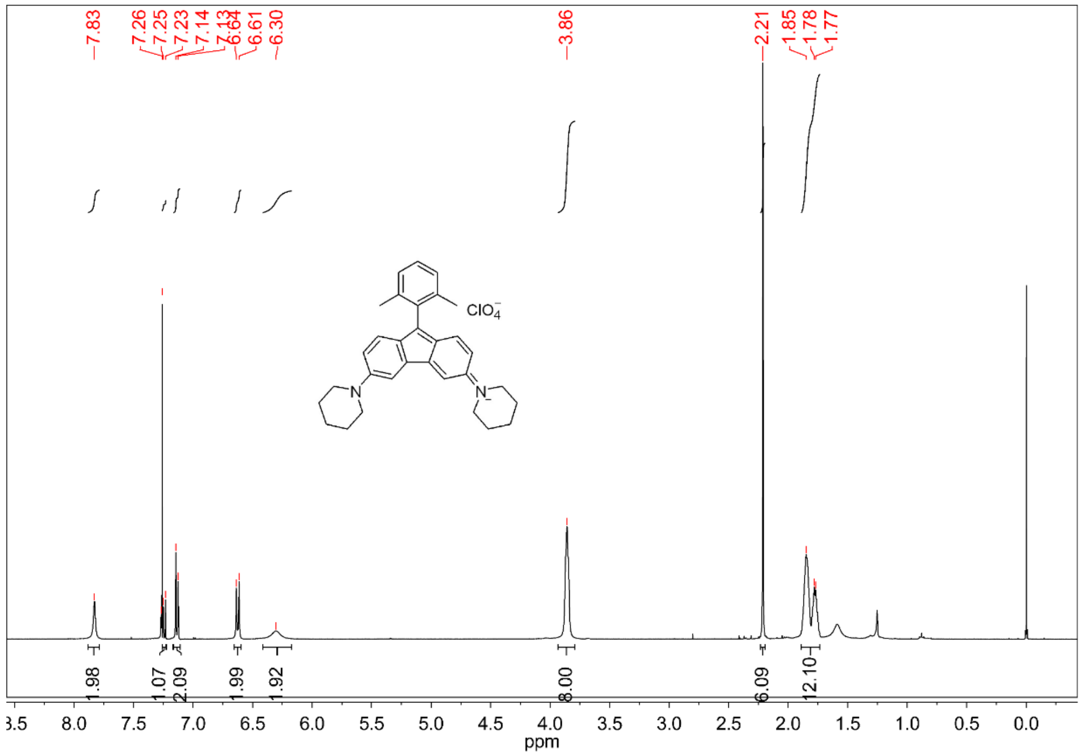

Supplementary Figure 73 | ^{1}H-NMR of AF9.

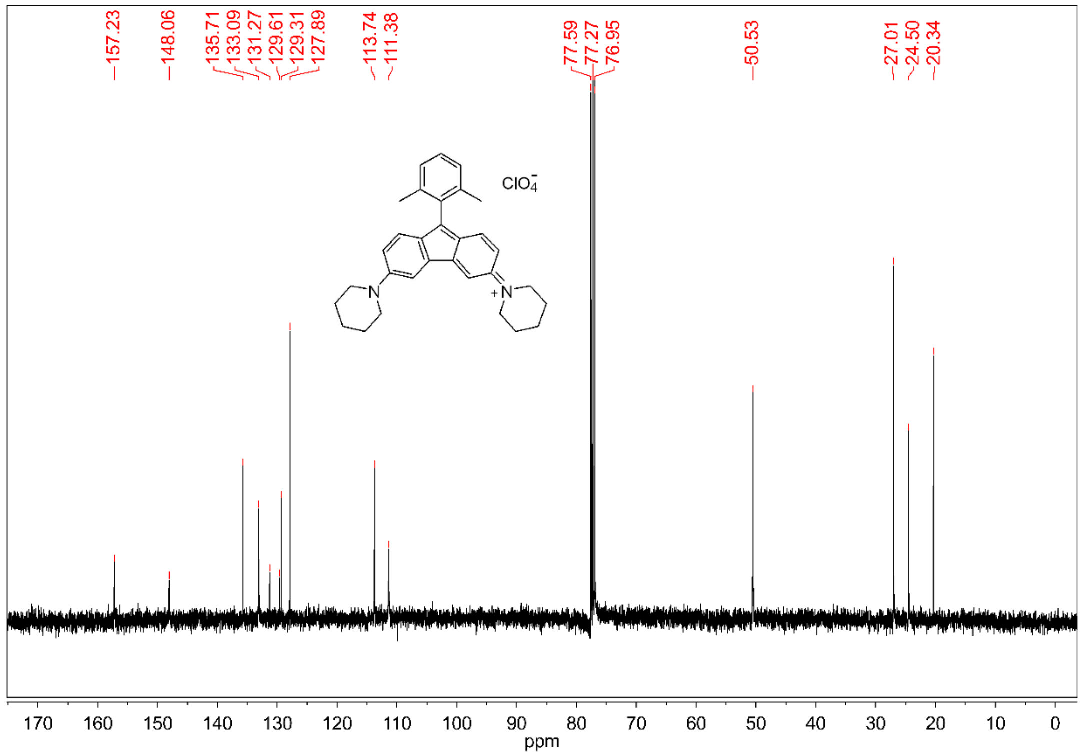

Supplementary Figure 74 | ^{13}C-NMR of AF9.

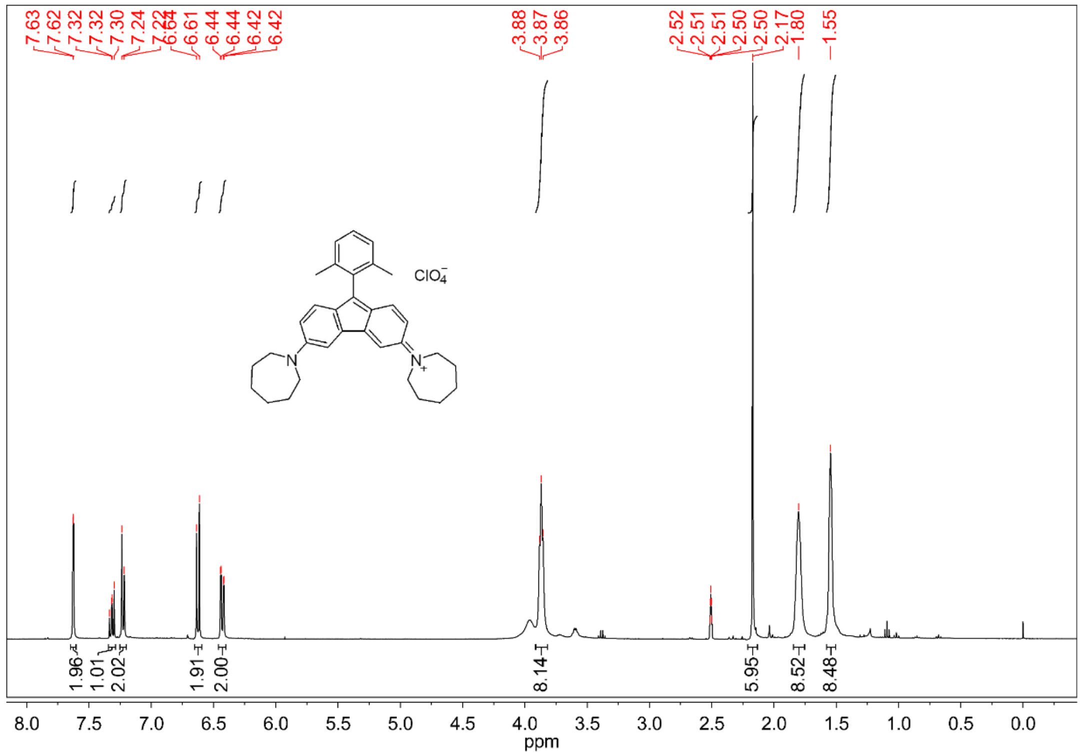

Supplementary Figure 75 | ^{1}H-NMR of AF10.

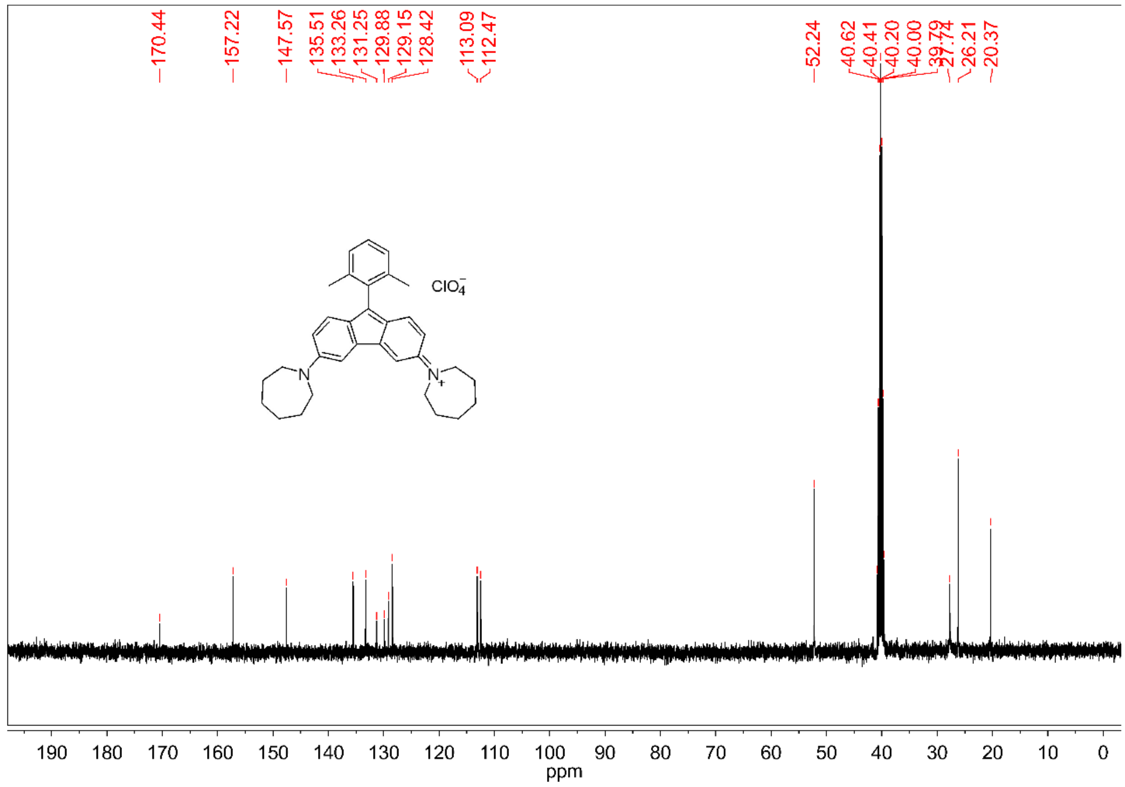

Supplementary Figure 76 | ^{13}C-NMR of AF10.

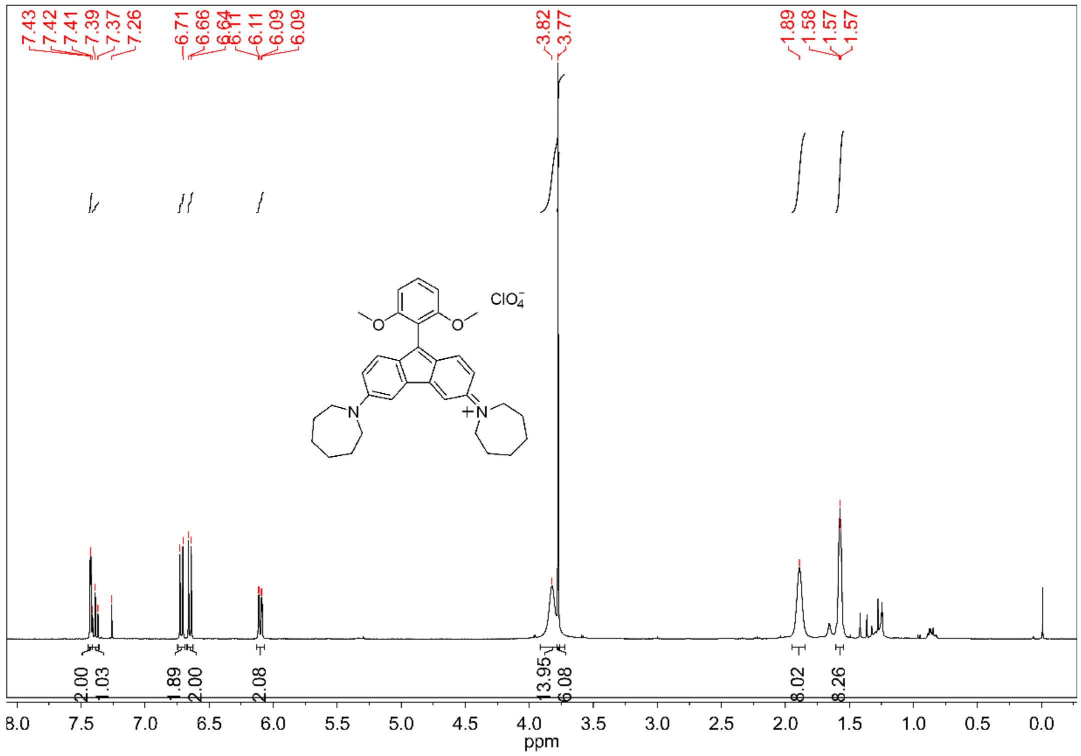

Supplementary Figure 77 | ^{1}H-NMR of AF11.

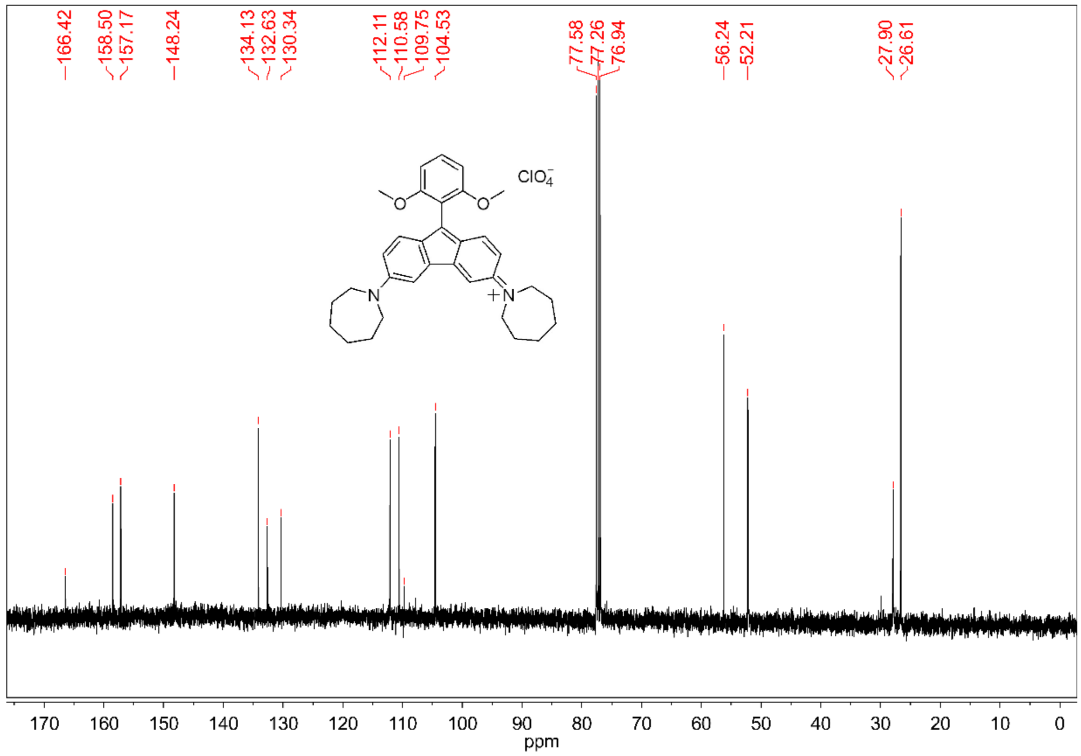

Supplementary Figure 78 | ^{13}C-NMR of AF11.

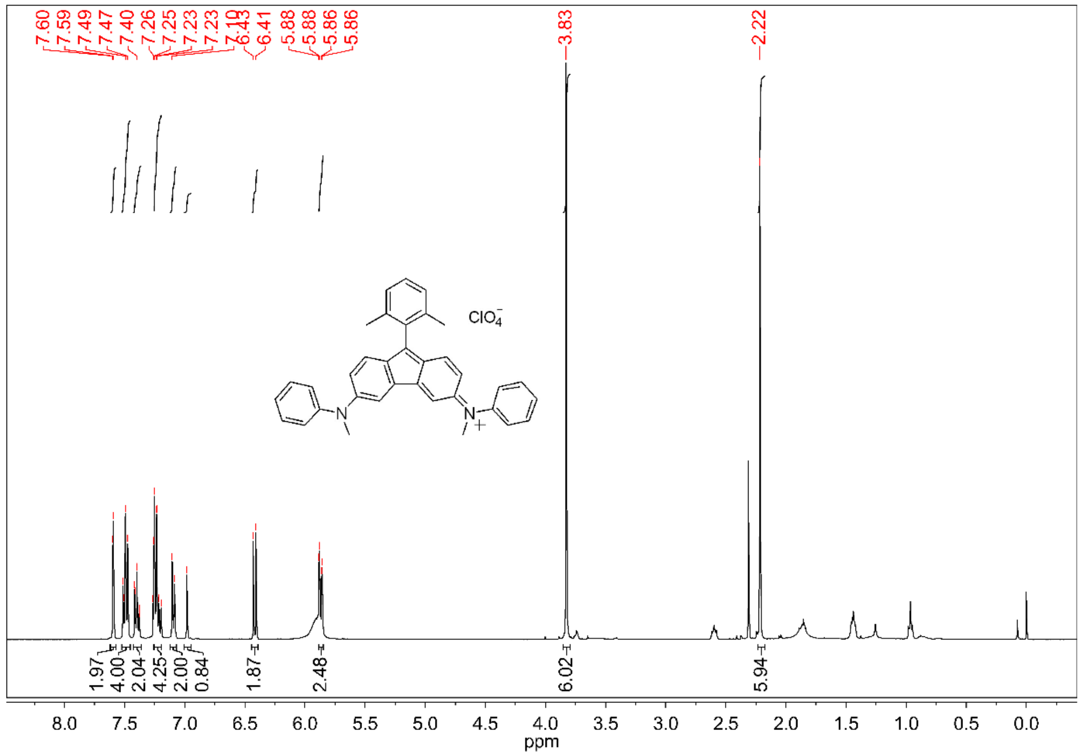

Supplementary Figure 79 | ^{1}H-NMR of AF12.

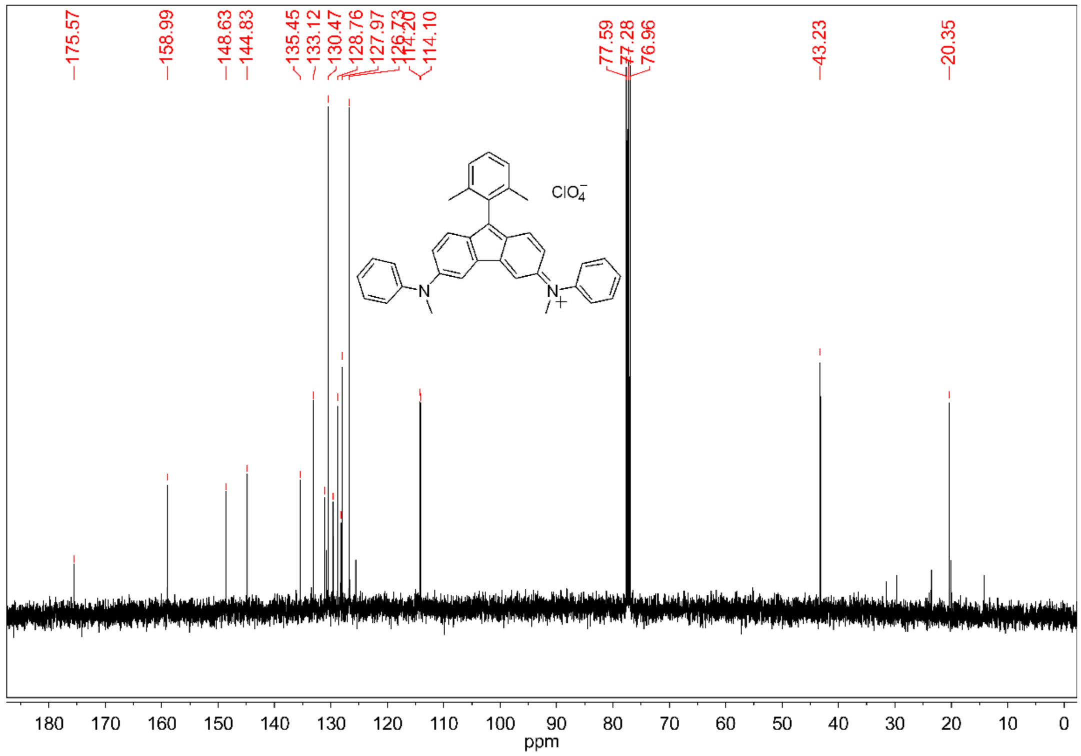

Supplementary Figure 80 | ^{13}C-NMR of AF12.

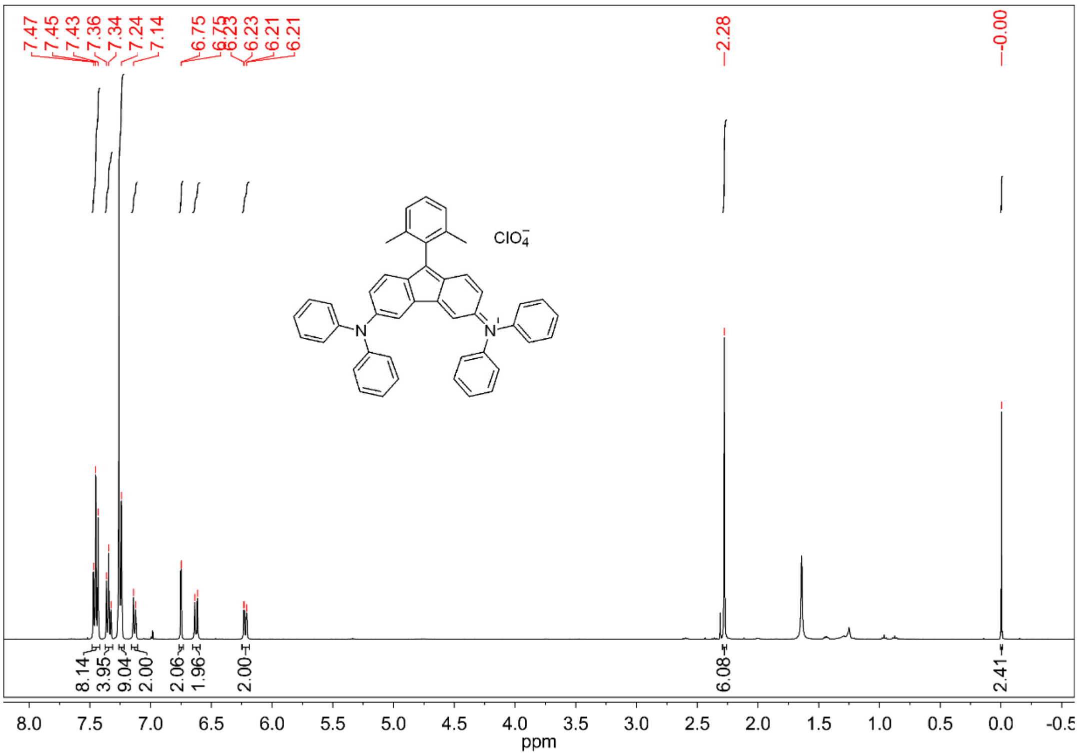

Supplementary Figure 81 | ^{1}H-NMR of AF13.

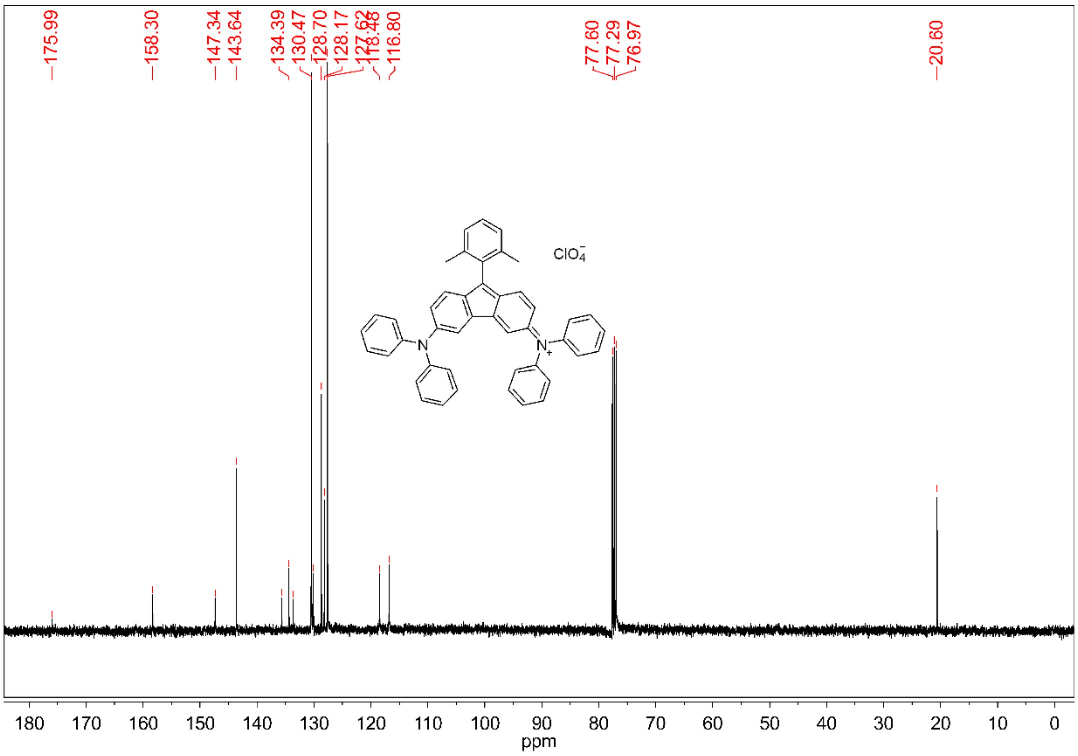

Supplementary Figure 82 | ^{13}C-NMR of AF13.

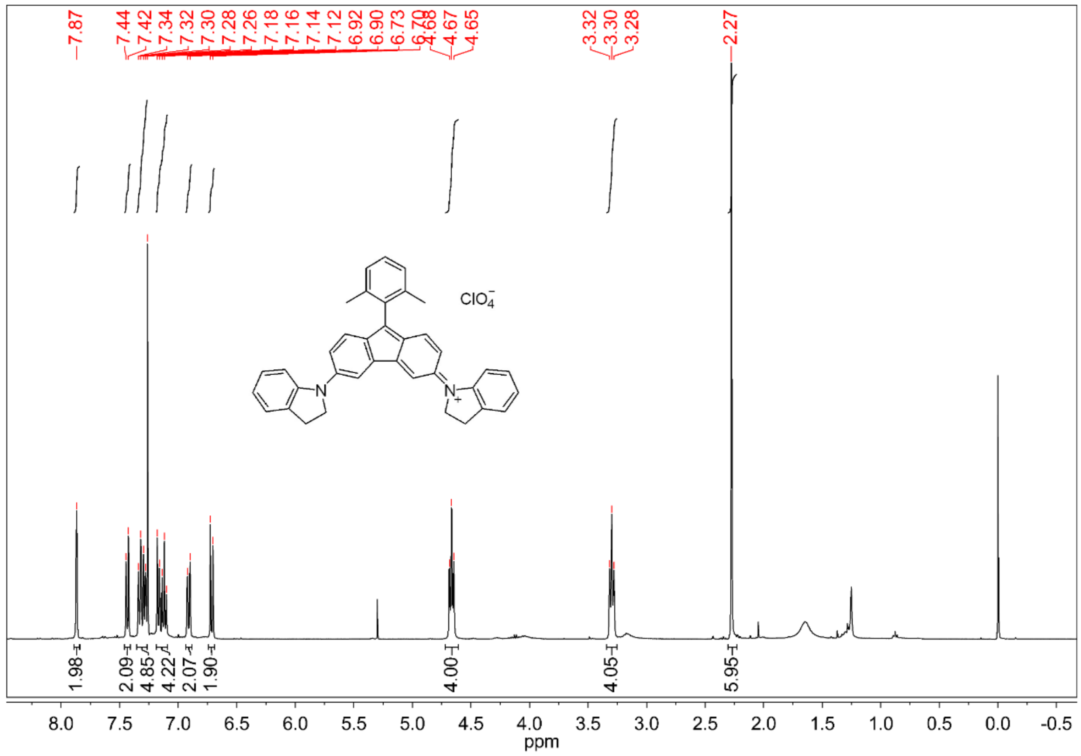

Supplementary Figure 83 | ^{1}H-NMR of AF14.

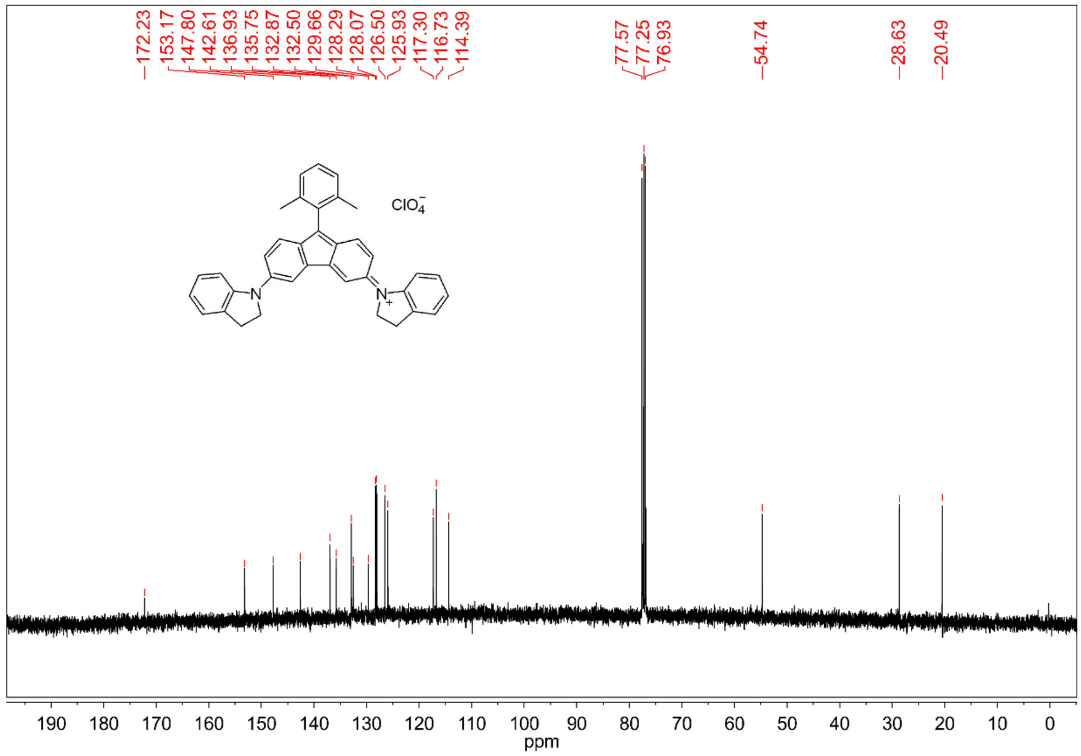

Supplementary Figure 84 | ^{13}C-NMR of AF14.

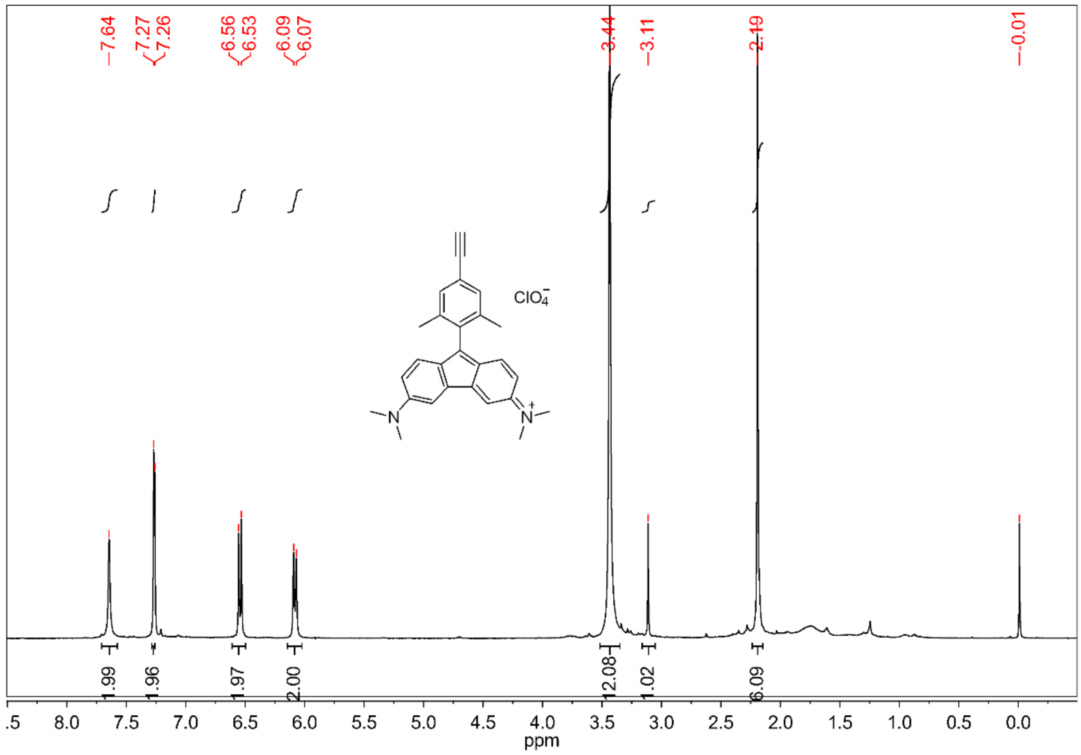

Supplementary Figure 85 | ^{1}H-NMR of AF3-Alkyne.

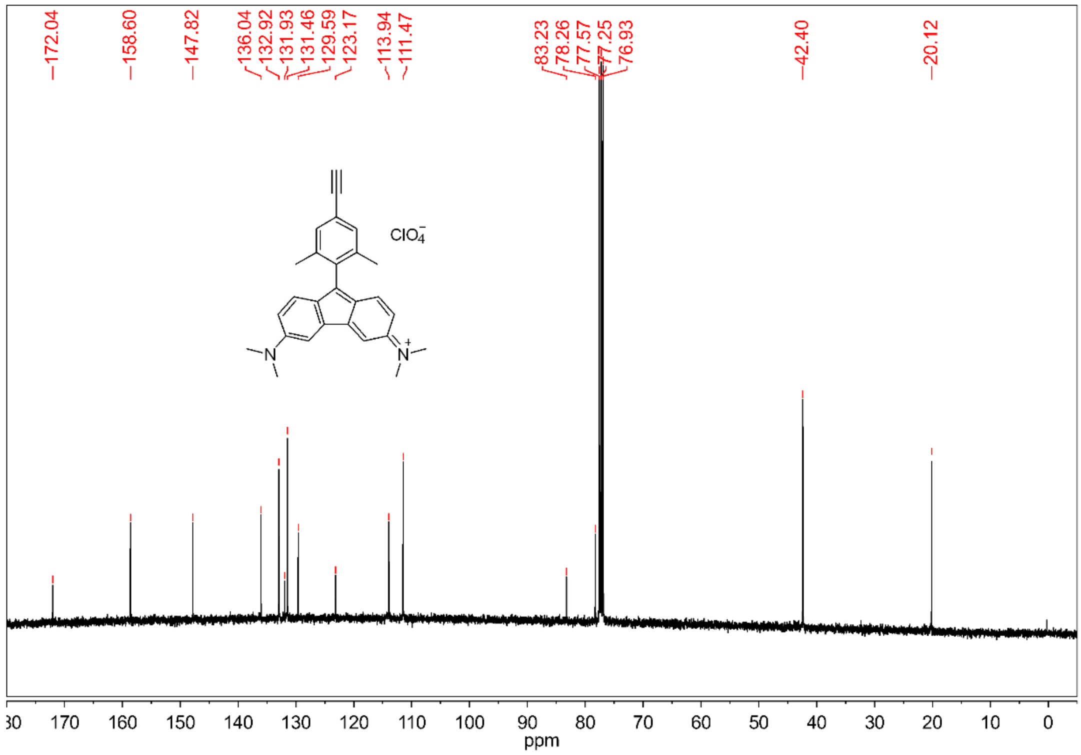

Supplementary Figure 86 | ^{13}C-NMR of AF3-Alkyne.

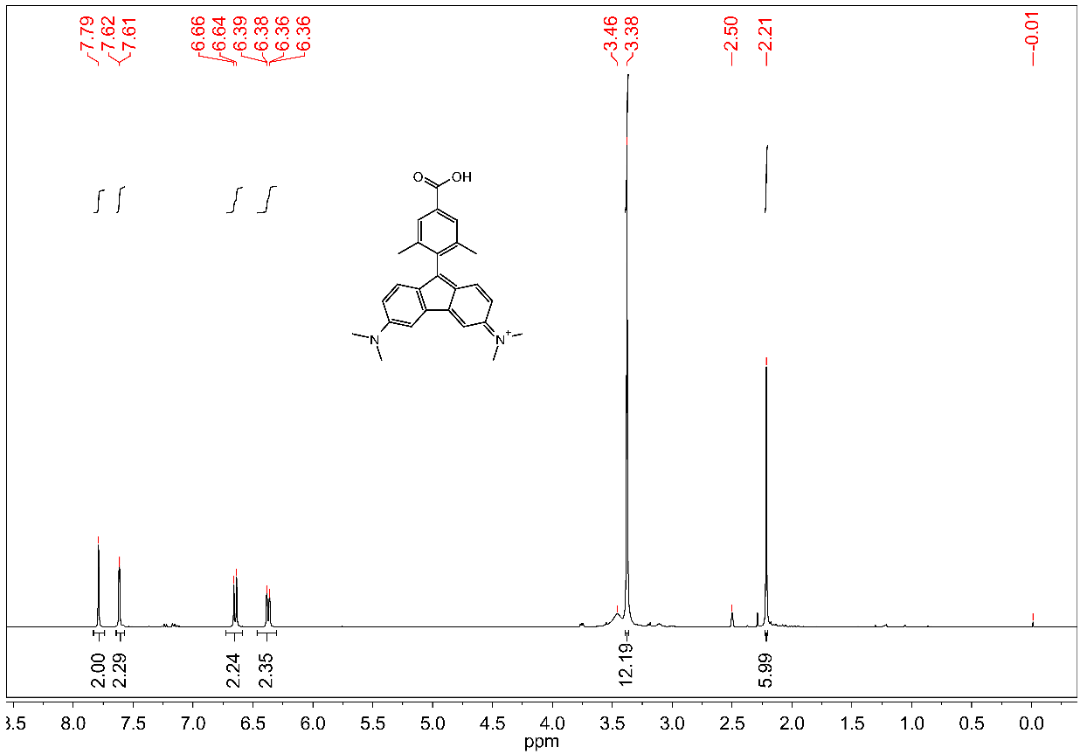

Supplementary Figure 87 | ^{1}H-NMR of AF3-COOH.

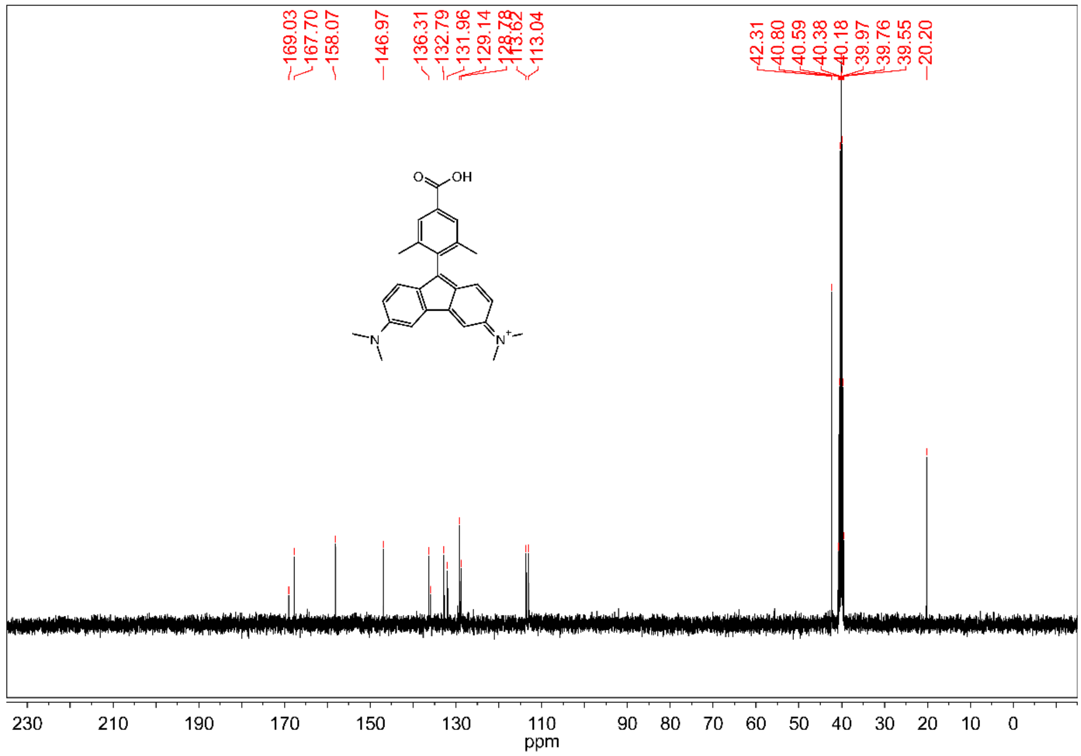

Supplementary Figure 88 | ^{13}C-NMR of AF3-COOH.

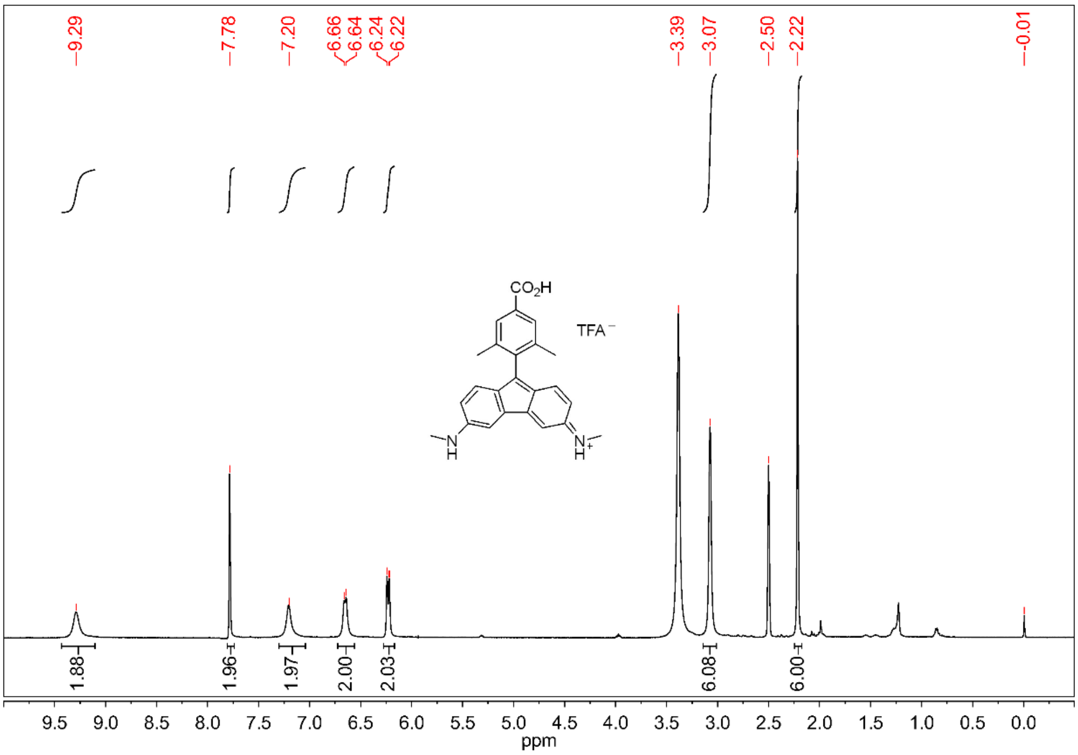

Supplementary Figure 89 | ^{1}H-NMR of AF2-COOH.

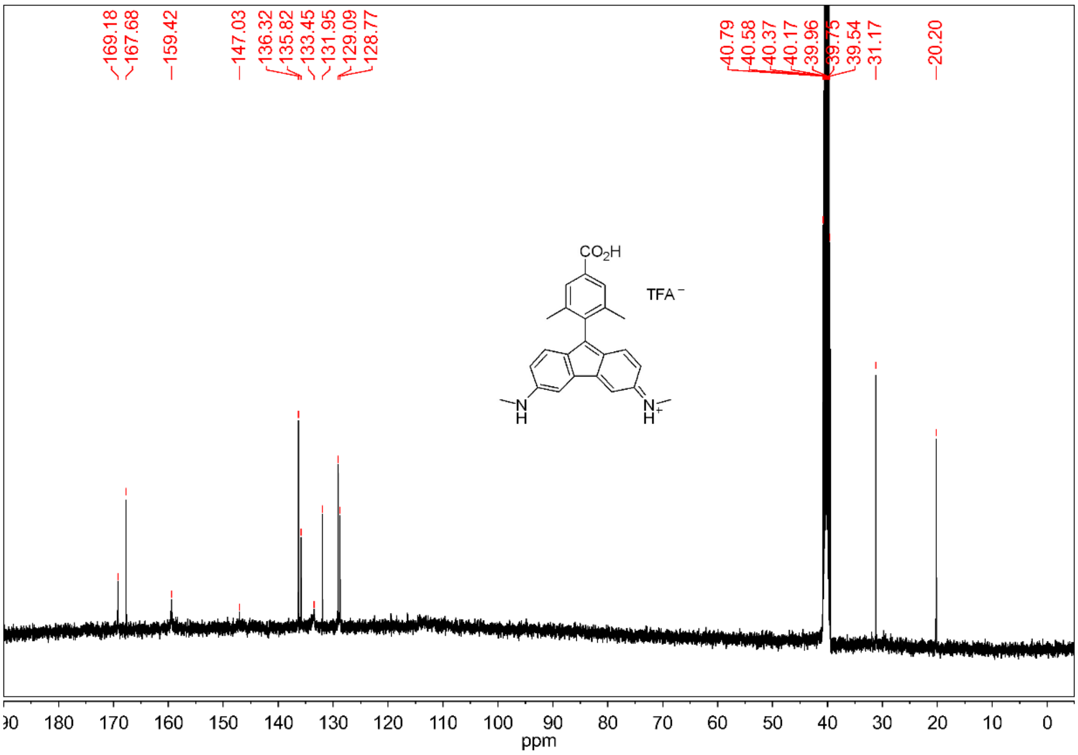

Supplementary Figure 90 | ^{13}C-NMR of AF2-COOH.

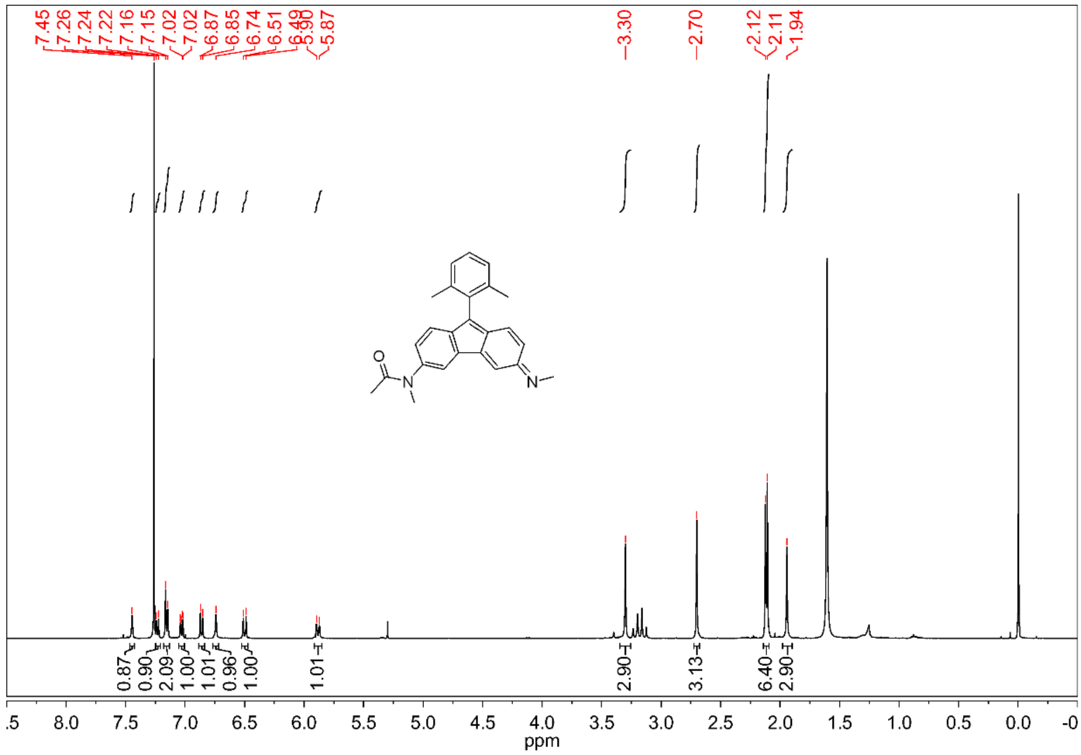

Supplementary Figure 91 | ^{1}H-NMR of AF2Ac.

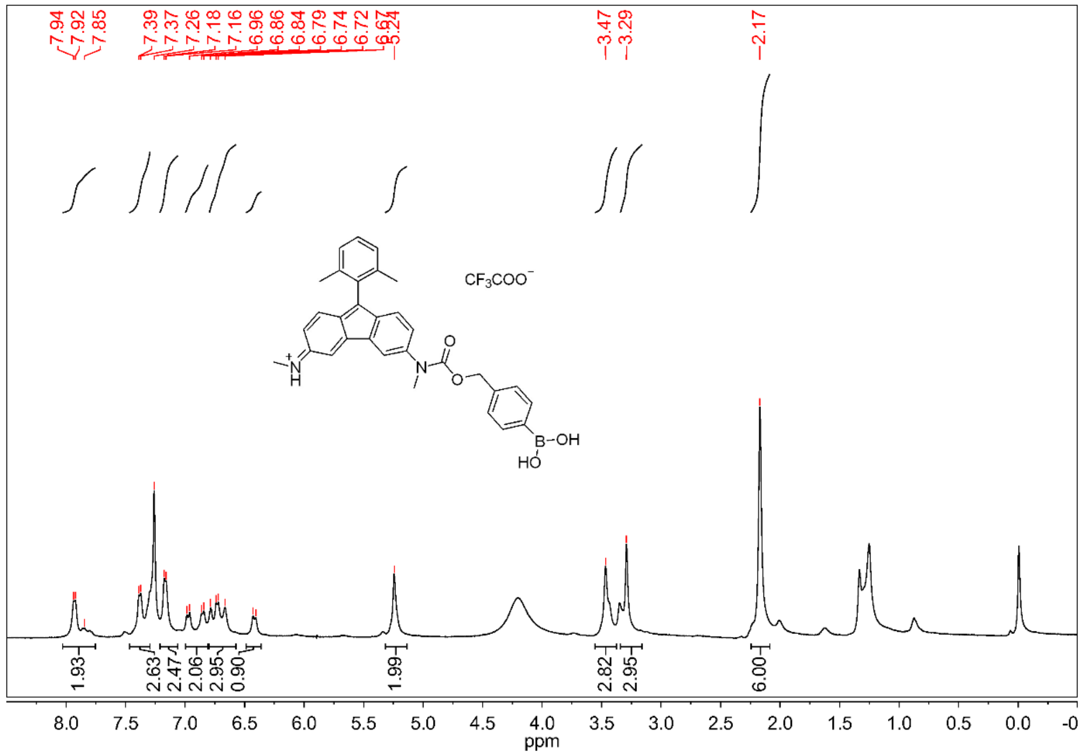

Supplementary Figure 92 | ^{1}H-NMR of AF2B.

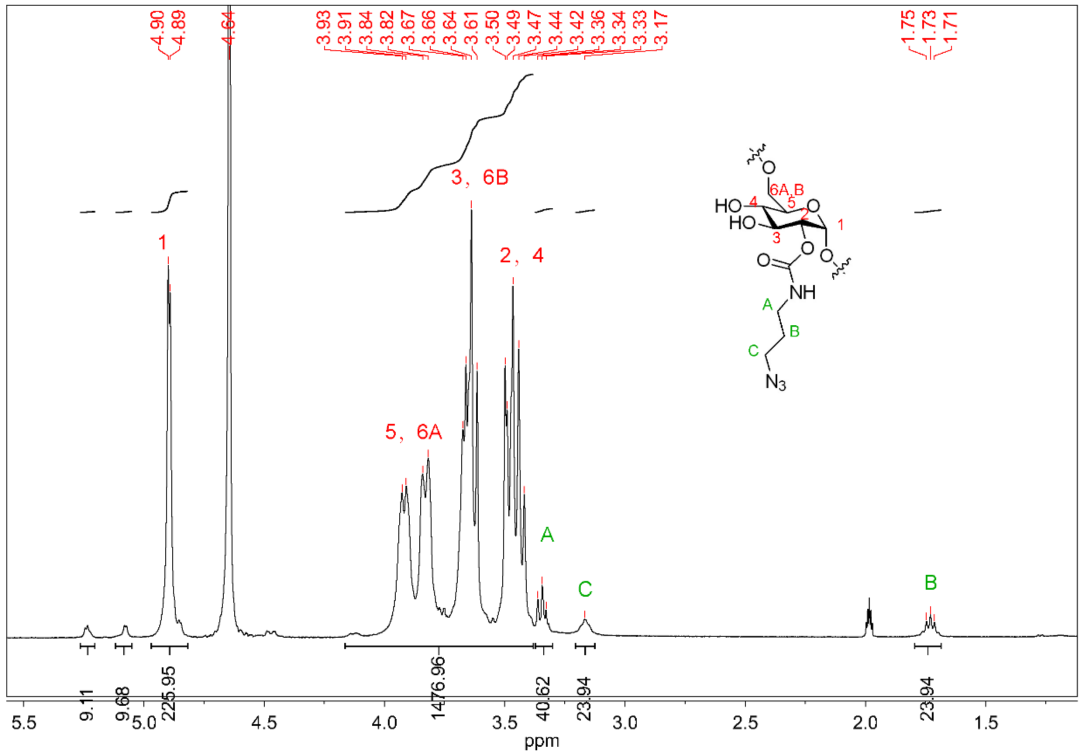

Supplementary Figure 93 | ^{1}H-NMR of Dextran-N_3. The total integration of all dextran C-H signals was set to 1726.13 (7 protons × 246.59 glucose units per 40000 Da dextran). The integral of signal (green B) was used to determine the average number of azidopropyl linker groups per dextran-40000 (23.94/2=11.97)

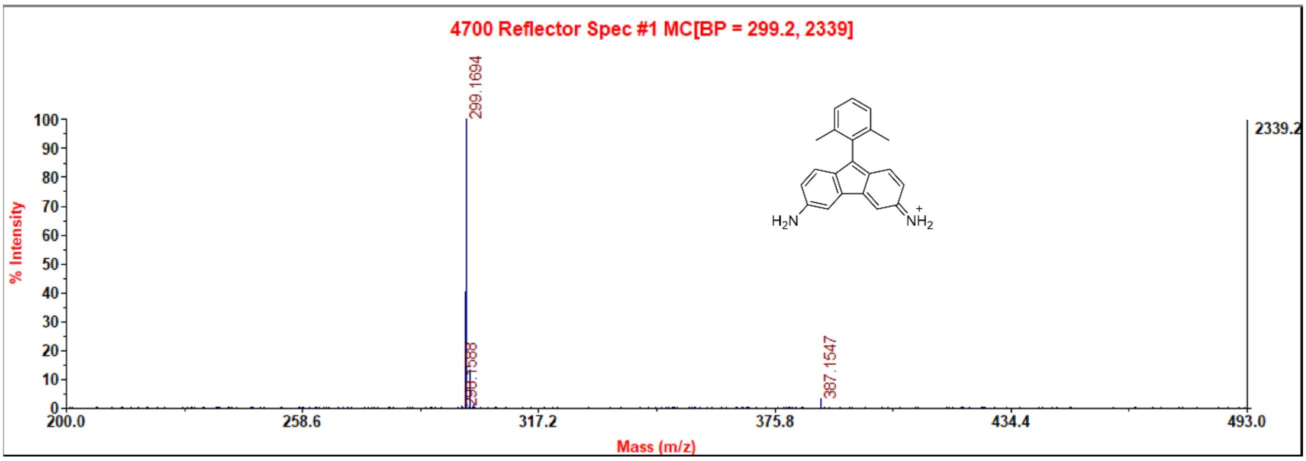

Supplementary Figure 94 | MALDI-TOF MS spectra of AF1.

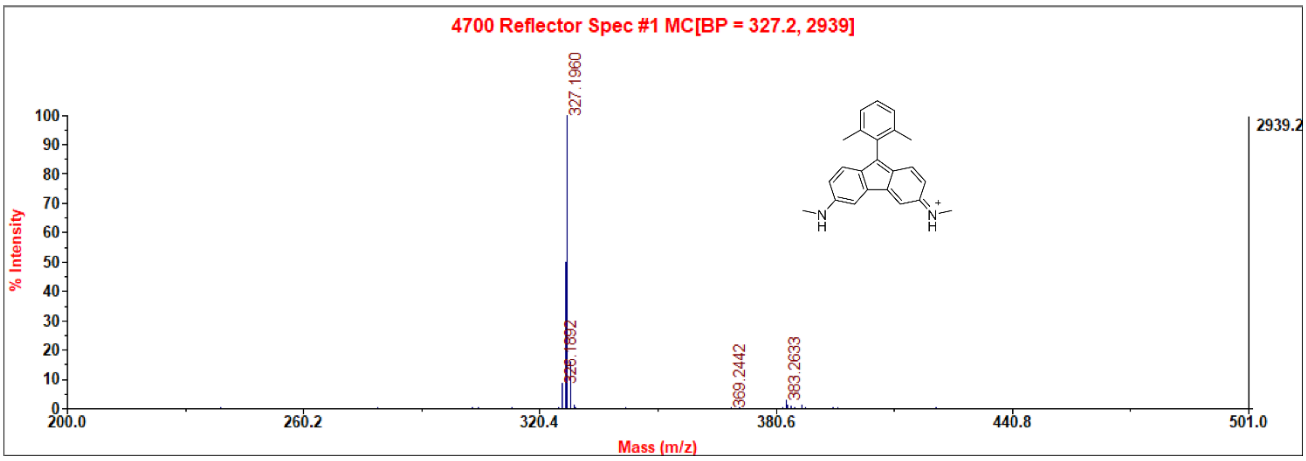

Supplementary Figure 95 | MALDI-TOF MS spectra of AF2.

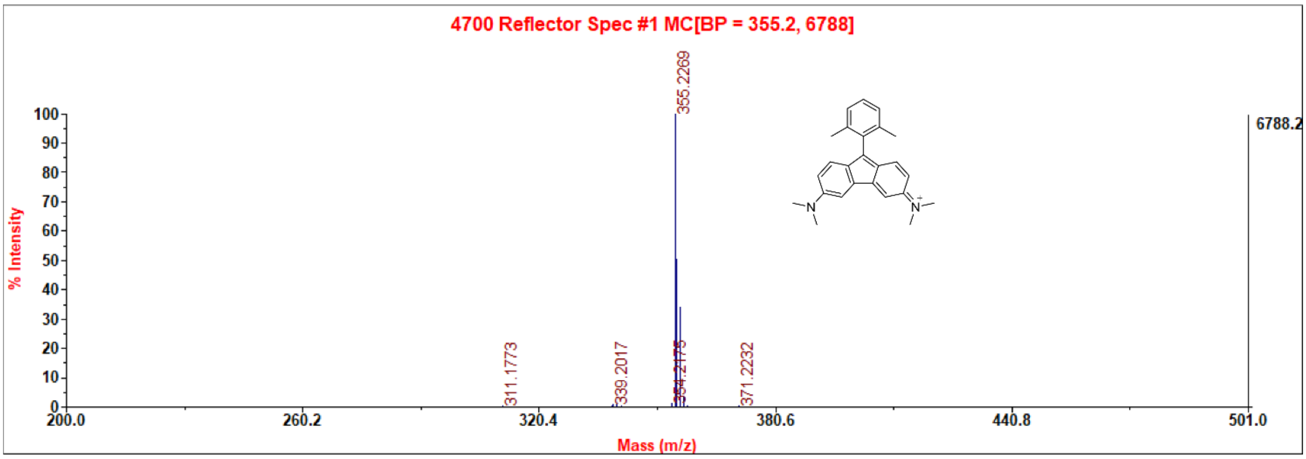

Supplementary Figure 96 | MALDI-TOF MS spectra of AF3.

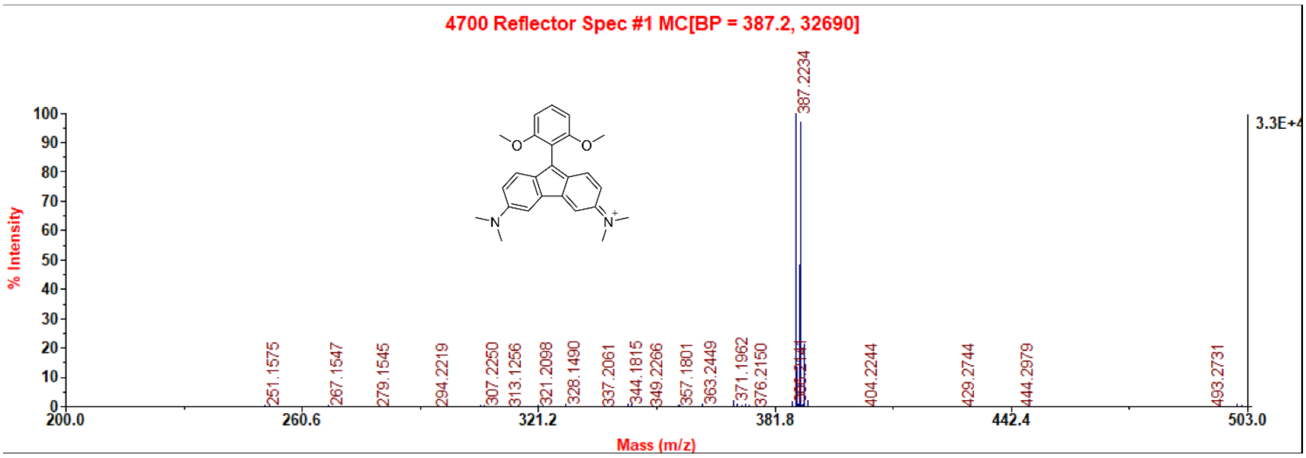

Supplementary Figure 97 | MALDI-TOF MS spectra of AF4.

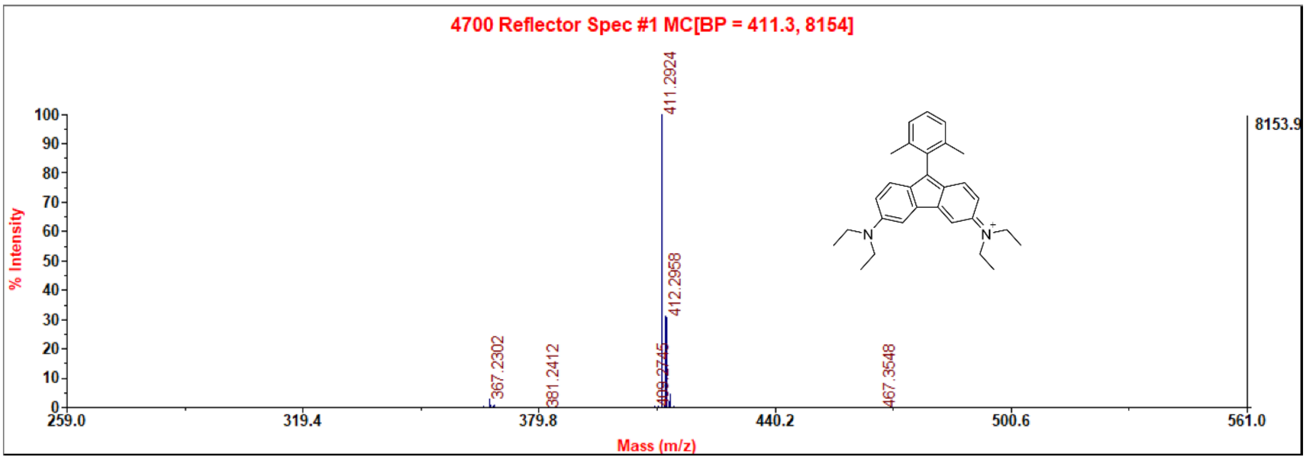

Supplementary Figure 98 | MALDI-TOF MS spectra of AF5.

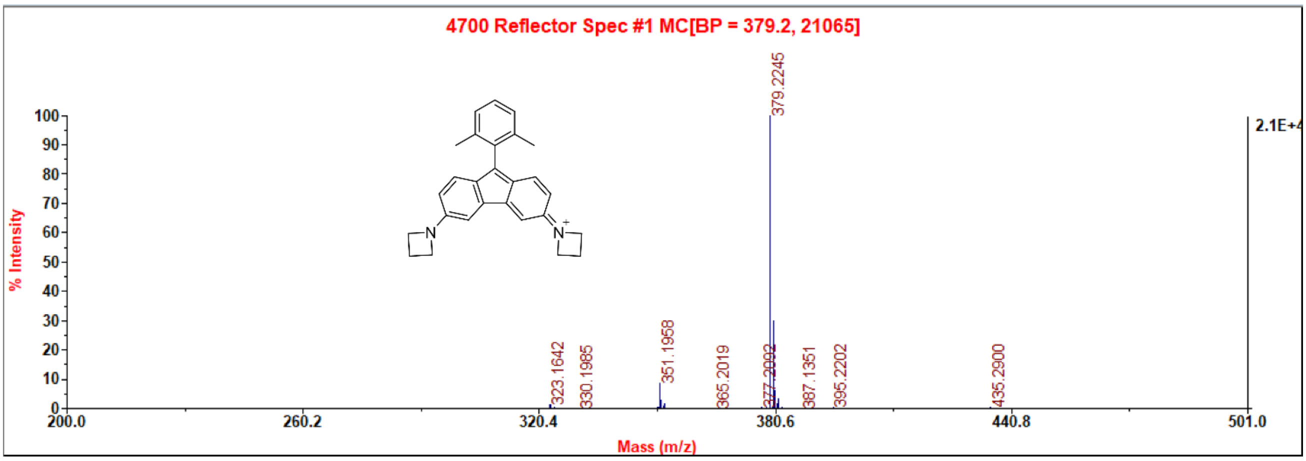

Supplementary Figure 99 | MALDI-TOF MS spectra of AF6.

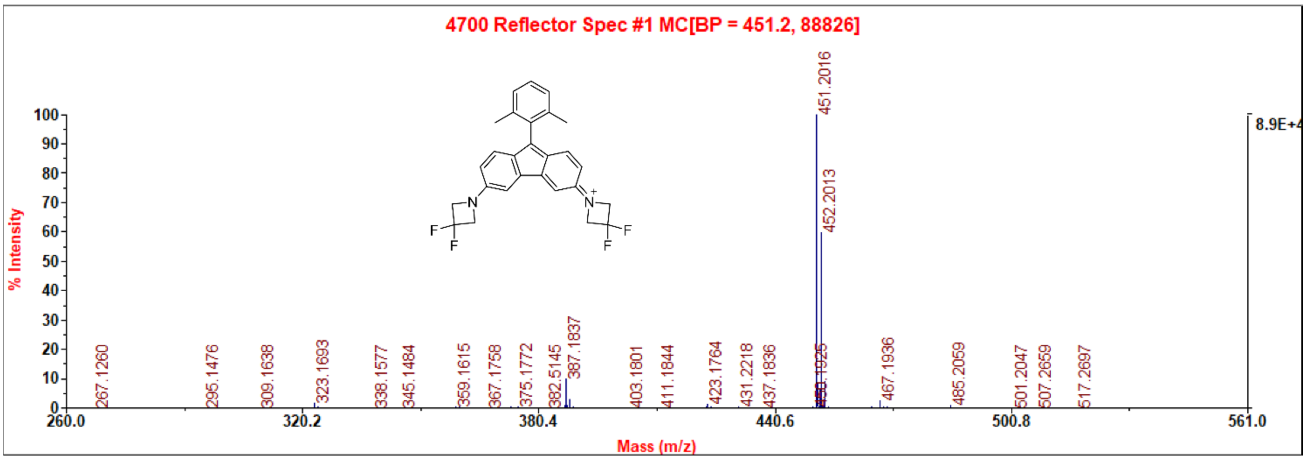

Supplementary Figure 100 | MALDI-TOF MS spectra of AF7.

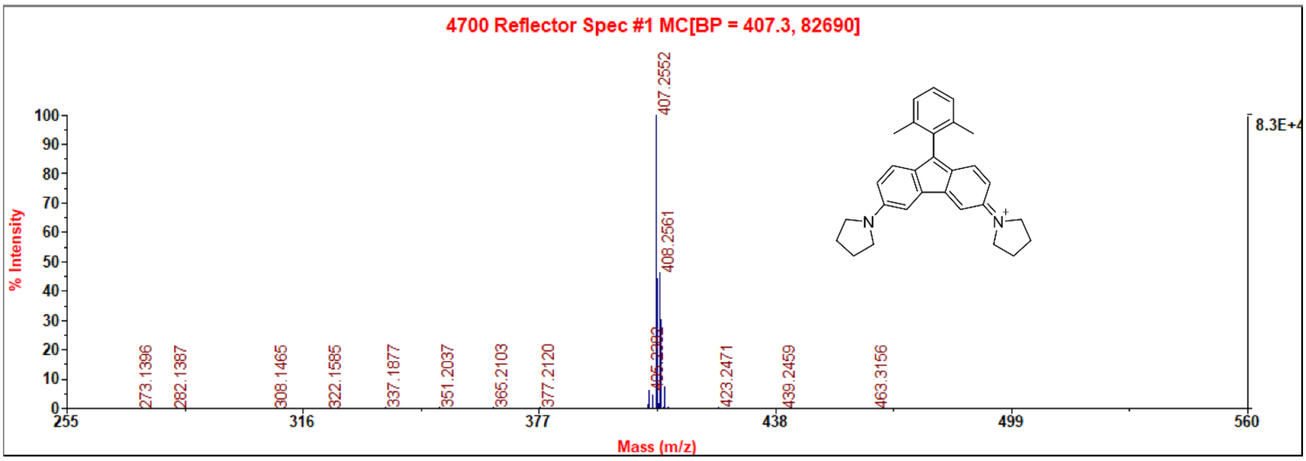

Supplementary Figure 101 | MALDI-TOF MS spectra of AF8.

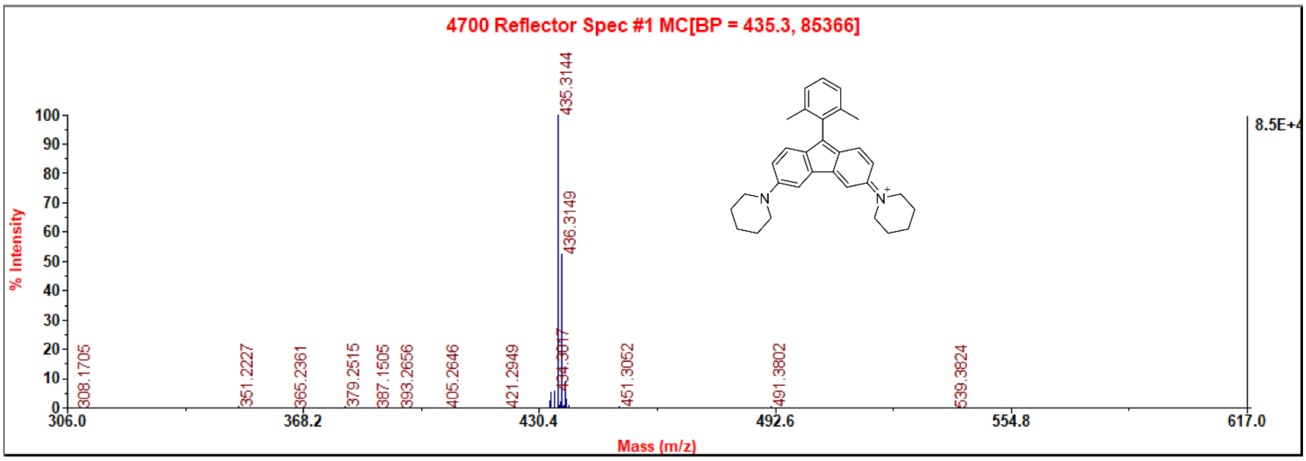

Supplementary Figure 102 | MALDI-TOF MS spectra of AF9.

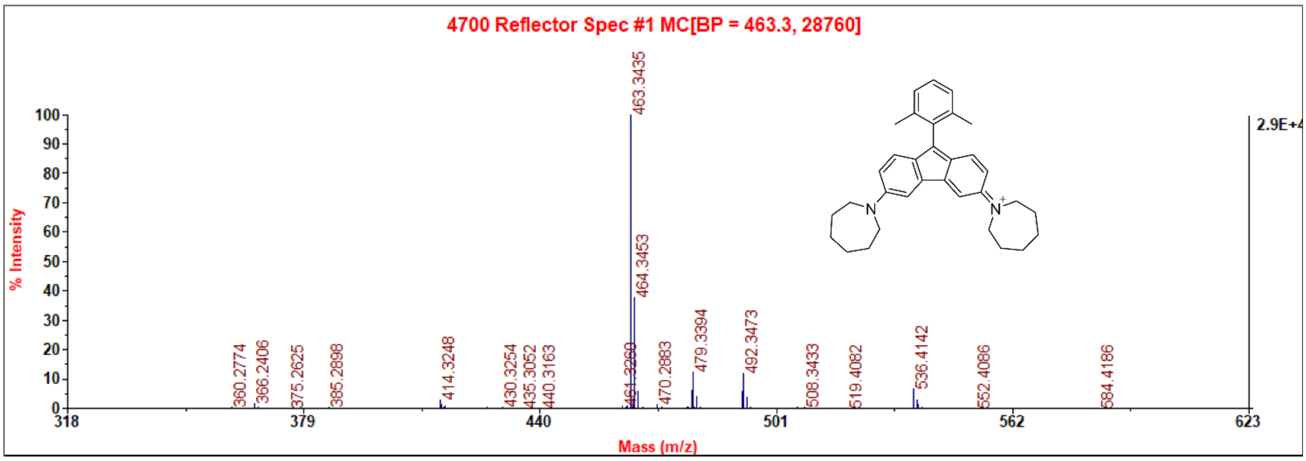

Supplementary Figure 103 | MALDI-TOF MS spectra of AF10.

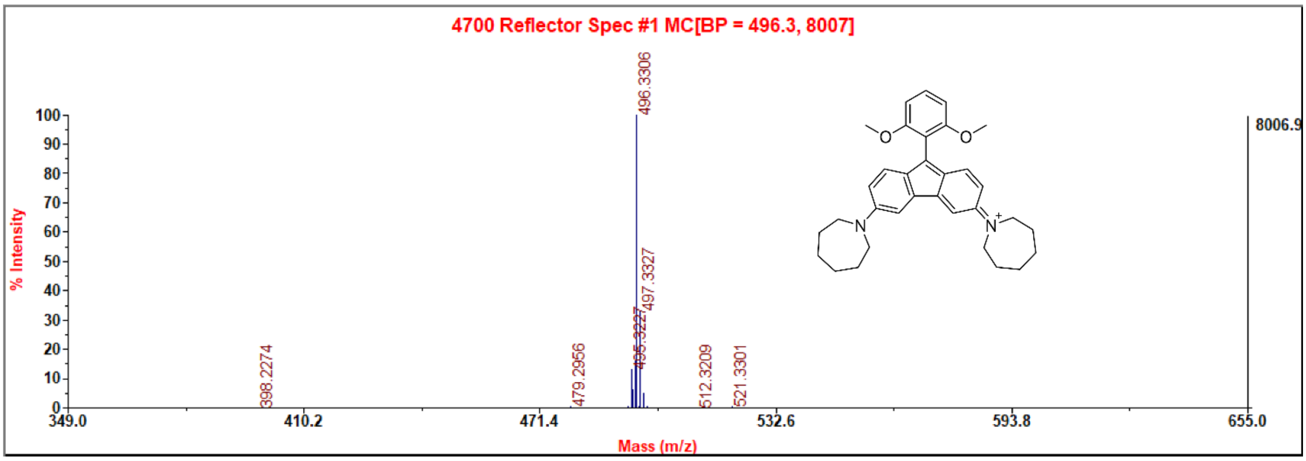

Supplementary Figure 104 | MALDI-TOF MS spectra of AF11.

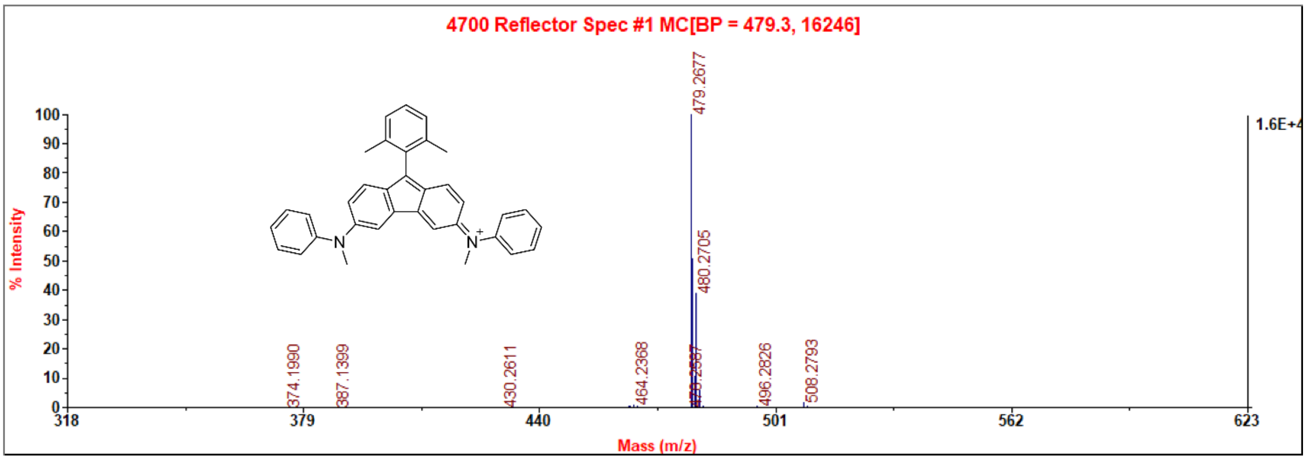

Supplementary Figure 105 | MALDI-TOF MS spectra of AF12.

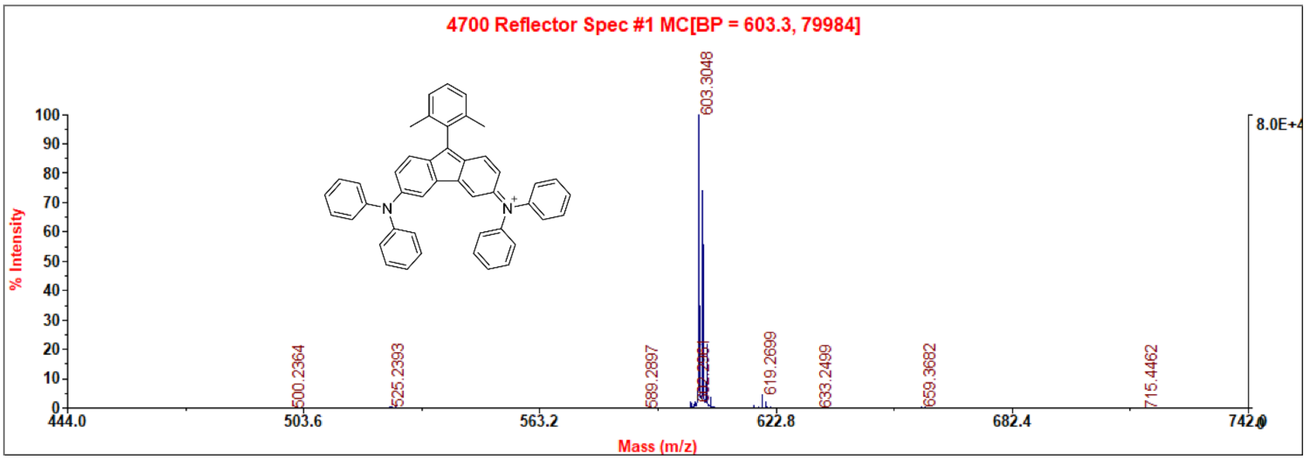

Supplementary Figure 106 | MALDI-TOF MS spectra of AF13.

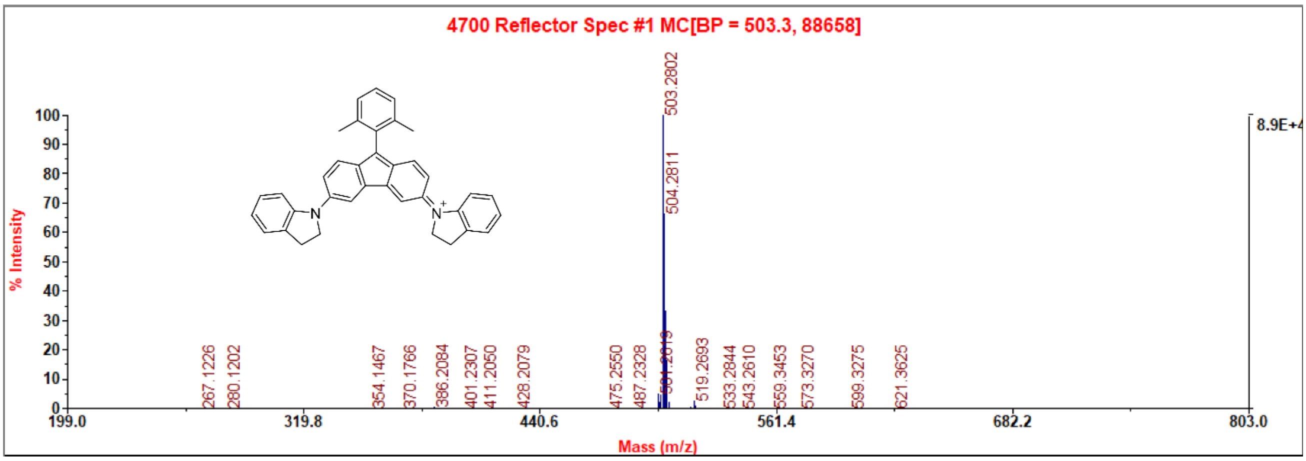

Supplementary Figure 107 | MALDI-TOF MS spectra of AF14.

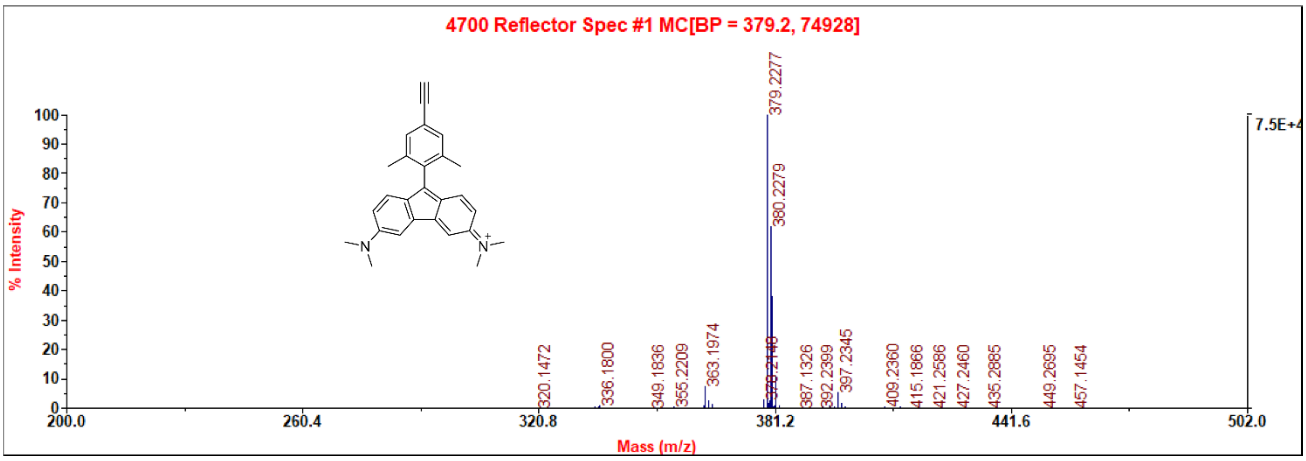

Supplementary Figure 108 | MALDI-TOF MS spectra of AF3-Alkyne.

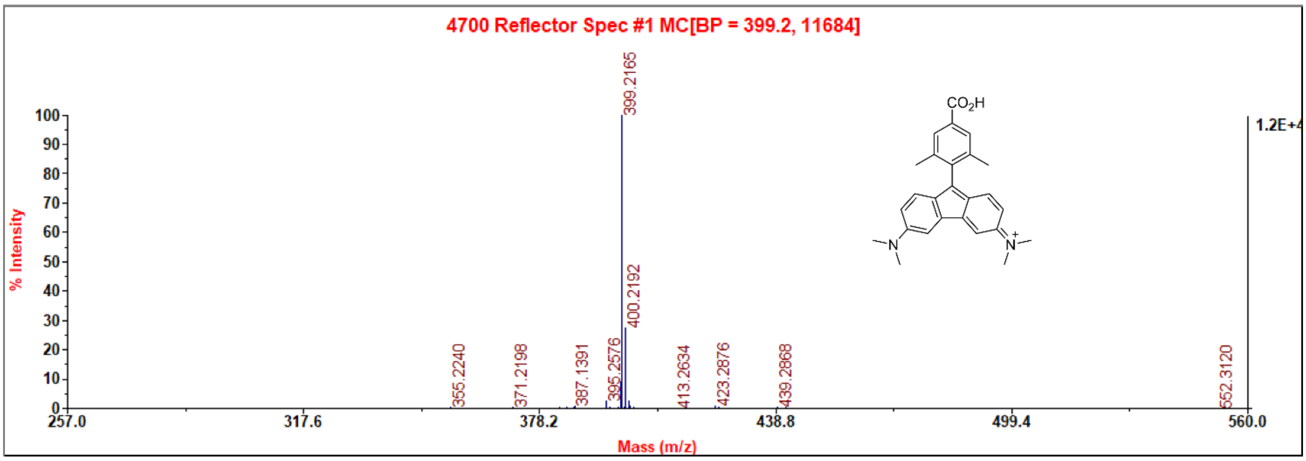

Supplementary Figure 109 | MALDI-TOF MS spectra of AF3-COOH.

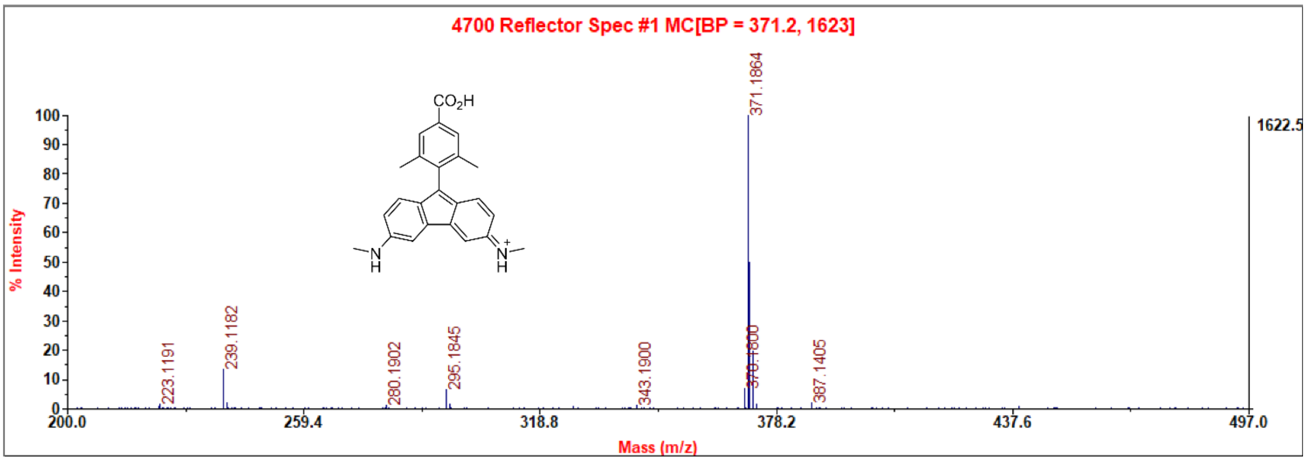

Supplementary Figure 110 | MALDI-TOF MS spectra of AF2-COOH.

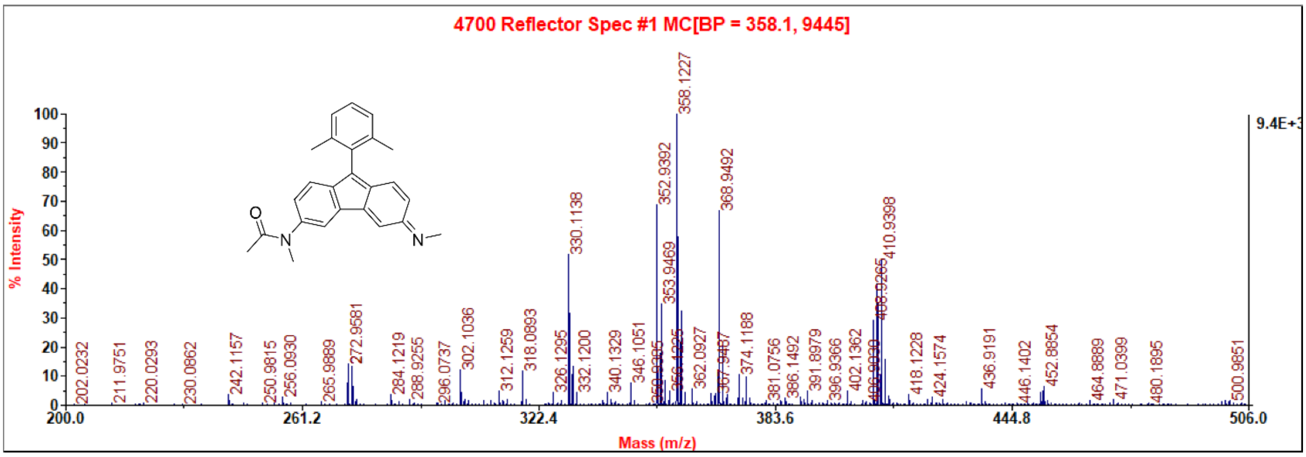

Supplementary Figure 111 | MALDI-TOF MS spectra of AF2Ac.

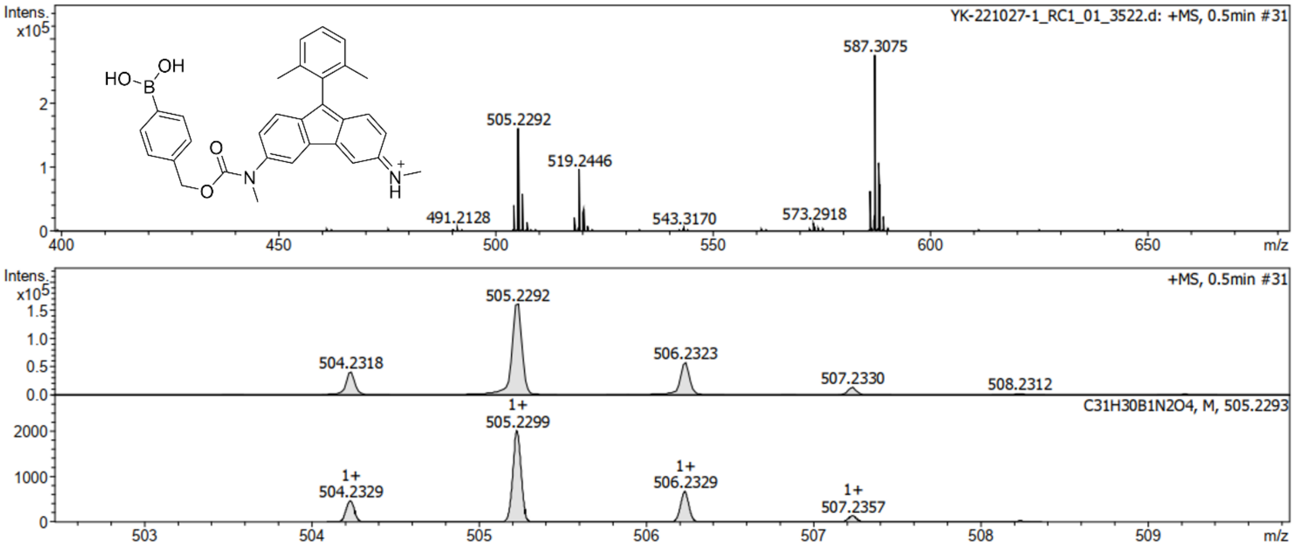

Supplementary Figure 112 | HRMS spectra of AF2B.

Supplementary Notes

Supplementary note for Supplementary Figure 22

For MCI,[45,46] the larger the MCI value, the stronger the aromaticity. Considering the fact that the typical TDDFT underestimates MCI values for S_1 state, the MCI values for T_1 state possessing similar excited state characters vs. S_1 state (Figure S21a) are obtained using unrestricted KS-DFT method. It can be seen that the MCI values are in the order of T_1-UDFT (0.1126) > S_1-TD/TDA (0.1051/0.1039) > S_1-ΔSCF (0.1030) > S_0 (0.1025). In addition, the MCI values for both S_0 and S_1 states are also computed at the CASSCF(2,2)/6-31G(d) level. All the calculated MCI values of T_1 states are larger than that of S_0 state, suggesting more aromatic character of excited state. Last but not least, it should be noted that the MCI is not a good choice to determine the antiaromaticity since the antiaromatic systems typically possessing very small MCI that is indistinguishable from those with weak aromaticity.[43]

For ELF-π index,[47] the space enclosed by the higher ELF values makes it easier for electrons to delocalize within this space and simultaneously more difficult to populate outside of this space. The position indicated by the red arrow is called a bifurcation point, on which larger ELF-π values suggest the more aromatic. According to the definition of ELF-π value,[48] the molecules with ELF-π of 0.17~0.35 correspond to an antiaromatic character, and ELF-π of > 0.70 to aromatic character. Thus, it can be seen that the ELF-π value of 0.18 for the S_0 state of AF3 indicates its antiaromaticity character. And the ELF-π value for the S_1 state is calculated to be 0.47 using the ΔSCF method. Note that usage of ELF-π to analyze the triplet state Baird-aromaticity is proved by Ottosson et al.,[49] herein the ELF-π value for T_1 state is calculated to be 0.37 using the unrestricted KS-DFT method. Unfortunately, these obtained ELF-π values for excited states are significantly smaller than the reference value of 0.70, suggesting not aromatic characters at all.

Supplementary References

(1) Semonin, O. E.; Johnson, J. C.; Luther, J. M.; Midgett, A. G.; Nozik, A. J.; Beard, M. C., Absolute Photoluminescence Quantum Yields of IR-26 Dye, PbS, and PbSe Quantum Dots. *J. Phys. Chem. Lett.* **2010,** *1* (16), 2445-2450.

(2) Uppu, R. M.; Pryor, W. A. Synthesis of peroxynitrite in a two-phase system using isoamyl nitrite and hydrogen peroxide. *Anal. Biochem.* **1996,** *236* (2), 242-249.

(3) Fiala, T.; Wang, J.; Dunn, M.; Šebej, P.; Choi, S. J.; Nwadibia, E. C.; Fialova, E.; Martinez, D. M.; Cheetham, C. E.; Fogle, K. J.; Palladino, M. J.; Freyberg, Z.; Sulzer, D.; Sames, D., Chemical Targeting of Voltage Sensitive Dyes to Specific Cells and Molecules in the Brain. *J. Am. Chem. Soc.* **2020,** *142* (20), 9285-9301.

(4) Ando, N.; Soutome, H.; Yamaguchi, S., Near-infrared fluorescein dyes containing a tricoordinate boron atom. *Chem. Sci.* **2019,** *10* (33), 7816-7821.

(5) Sibrian-Vazquez, M.; Escobedo, J. O.; Lowry, M.; Fronczek, F. R.; Strongin, R. M., Field effects induce bathochromic shifts in xanthene dyes. *J. Am. Chem. Soc.* **2012,** *134* (25), 10502-8.

(6) Fu, M.; Xiao, Y.; Qian, X.; Zhao, D.; Xu, Y., A design concept of long-wavelength fluorescent analogs of rhodamine dyes: replacement of oxygen with silicon atom. *Chem. Commun.* **2008,** (15), 1780-2.

(7) Koide, Y.; Urano, Y.; Hanaoka, K.; Terai, T.; Nagano, T., Evolution of group 14 rhodamines as platforms for near-infrared fluorescence probes utilizing photoinduced electron transfer. *ACS Chem. Biol.* **2011,** *6* (6), 600-8.

(8) Koide, Y.; Urano, Y.; Hanaoka, K.; Piao, W.; Kusakabe, M.; Saito, N.; Terai, T.; Okabe, T.; Nagano, T., Development of NIR Fluorescent Dyes Based on Si–rhodamine for in Vivo Imaging. *J. Am. Chem. Soc.* **2012,** *134* (11), 5029-5031.

(9) Liu, J.; Sun, Y. Q.; Zhang, H.; Shi, H.; Shi, Y.; Guo, W., Sulfone-Rhodamines: A New Class of Near-Infrared Fluorescent Dyes for Bioimaging. *ACS Appl. Mater. Interfaces* **2016,** *8* (35), 22953-62.

(10) Choi, A.; Miller, S. C., Silicon Substitution in Oxazine Dyes Yields Near-Infrared Azasiline Fluorophores That Absorb and Emit beyond 700 nm. *Org. Lett.* **2018,** *20* (15), 4482-4485.

(11) Fukazawa, A.; Usuba, J.; Adler, R. A.; Yamaguchi, S., Synthesis of seminaphtho-phospha-fluorescein dyes based on the consecutive arylation of aryldichlorophosphines. *Chem. Commun.* **2017,** *53* (61), 8565-8568.

(12) Sezukuri, K.; Suzuki, M.; Hayashi, H.; Kuzuhara, D.; Aratani, N.; Yamada, H., A laterally pi-expanded fluorone dye as an efficient near infrared fluorophore. *Chem. Commun.* **2016,** *52* (27), 4872-5.

(13) Rathnamalala, C. S. L.; Pino, N. W.; Herring, B. S.; Hooper, M.; Gwaltney, S. R.; Chan, J.; Scott, C. N.,

Thienylpiperidine Donor NIR Xanthene-Based Dye for Photoacoustic Imaging. *Org. Lett.* **2021,** *23* (19), 7640-7644.

(14) Lei, Z.; Li, X.; Luo, X.; He, H.; Zheng, J.; Qian, X.; Yang, Y., Bright, Stable, and Biocompatible Organic Fluorophores Absorbing/Emitting in the Deep Near-Infrared Spectral Region. *Angew. Chem. Int. Ed.* **2017,** *56* (11), 2979-2983.

(15) Lei, Z.; Zhang, F., Molecular Engineering of NIR-II Fluorophores for Improved Biomedical Detection. *Angew. Chem. Int. Ed.* **2021,** *60* (30), 16294-16308.

(16) Gandioso, A.; Bresoli-Obach, R.; Nin-Hill, A.; Bosch, M.; Palau, M.; Galindo, A.; Contreras, S.; Rovira, A.; Rovira, C.; Nonell, S.; Marchan, V., Redesigning the Coumarin Scaffold into Small Bright Fluorophores with Far-Red to Near-Infrared Emission and Large Stokes Shifts Useful for Cell Imaging. *J. Org. Chem.* **2018,** *83* (3), 1185-1195.

(17) Matikonda, S. S.; Ivanic, J.; Gomez, M.; Hammersley, G.; Schnermann, M. J., Core remodeling leads to long wavelength fluoro-coumarins. *Chem. Sci.* **2020,** *11* (28), 7302-7307.

(18) Labella, J.; Duran-Sampedro, G.; Krishna, S.; Martinez-Diaz, M. V.; Guldi, D. M.; Torres, T., Anthracene-Fused Oligo-BODIPYs: A New Class of pi-Extended NIR-Absorbing Materials. *Angew. Chem. Int. Ed.* **2023,** *62* (5), e202214543.

(19) Umezawa, K.; Nakamura, Y.; Makino, H.; Citterio, D.; Suzuki, K., Bright, Color-Tunable Fluorescent Dyes in the Visible−Near-Infrared Region. *J. Am. Chem. Soc.* **2008,** *130* (5), 1550-1551.

(20) Nakamura, M.; Tahara, H.; Takahashi, K.; Nagata, T.; Uoyama, H.; Kuzuhara, D.; Mori, S.; Okujima, T.; Yamada, H.; Uno, H., pi-Fused bis-BODIPY as a candidate for NIR dyes. *Org. Biomol. Chem.* **2012,** *10* (34), 6840-9.

(21) Patra, A.; Patalag, L. J.; Jones, P. G.; Werz, D. B., Extended Benzene-Fused Oligo-BODIPYs: In Three Steps to a Series of Large, Arc-Shaped, Near-Infrared Dyes. *Angew. Chem. Int. Ed.* **2021,** *60* (2), 747-752.

(22) Yu, C.; Jiao, L.; Li, T.; Wu, Q.; Miao, W.; Wang, J.; Wei, Y.; Mu, X.; Hao, E., Fusion and planarization of bisBODIPY: a new family of photostable near infrared dyes. *Chem. Commun.* **2015,** *51* (94), 16852-5.

(23) Bai, L.; Sun, P.; Liu, Y.; Zhang, H.; Hu, W.; Zhang, W.; Liu, Z.; Fan, Q.; Li, L.; Huang, W., Novel aza-BODIPY based small molecular NIR-II fluorophores for in vivo imaging. *Chem. Commun.* **2019,** *55* (73), 10920-10923.

(24) Huaulme, Q.; Mirloup, A.; Retailleau, P.; Ziessel, R., Synthesis of Highly Functionalized BOPHY Chromophores Displaying Large Stokes Shifts. *Org. Lett.* **2015,** *17* (9), 2246-9.

(25) Patalag, L. J.; Jones, P. G.; Werz, D. B., BOIMPYs: Rapid Access to a Family of Red-Emissive Fluorophores and NIR Dyes. *Angew. Chem. Int. Ed.* **2016,** *55* (42), 13340-13344.

(26) Killoran, J.; Allen, L.; Gallagher, J. F.; Gallagher, W. M.; O'Shea, D. F., Synthesis of BF2 chelates of tetraarylazadipyrromethenes and evidence for their photodynamic therapeutic behaviour. *Chem. Commun.* **2002,** (17),

1862-3.

(27) Zhang, X.; Yu, H.; Xiao, Y., Replacing phenyl ring with thiophene: an approach to longer wavelength aza-dipyrromethene boron difluoride (Aza-BODIPY) dyes. *J. Org. Chem.* **2012,** *77* (1), 669-73.

(28) Carr, J. A.; Franke, D.; Caram, J. R.; Perkinson, C. F.; Saif, M.; Askoxylakis, V.; Datta, M.; Fukumura, D.; Jain, R. K.; Bawendi, M. G.; Bruns, O. T., Shortwave infrared fluorescence imaging with the clinically approved near-infrared dye indocyanine green. *Proc. Natl. Acad. Sci. U.S.A.* **2018,** *115* (17), 4465-4470.

(29) Lei, Z.; Sun, C.; Pei, P.; Wang, S.; Li, D.; Zhang, X.; Zhang, F., Stable, Wavelength-Tunable Fluorescent Dyes in the NIR-II Region for In Vivo High-Contrast Bioimaging and Multiplexed Biosensing. *Angew. Chem. Int. Ed.* **2019,** *58* (24), 8166-8171.

(30) Li, B.; Lu, L.; Zhao, M.; Lei, Z.; Zhang, F., An Efficient 1064 nm NIR-II Excitation Fluorescent Molecular Dye for Deep-Tissue High-Resolution Dynamic Bioimaging. *Angew. Chem. Int. Ed.* **2018,** *57* (25), 7483-7487.

(31) Kopainsky, B.; Qiu, P.; Kaiser, W.; Sens, B.; Drexhage, K. H., Lifetime, photostability, and chemical structure of IR heptamethine cyanine dyes absorbing beyond 1 μm. *Appl. Phys. B* **1982,** *29* (1), 15-18.

(32) Cosco, E. D.; Caram, J. R.; Bruns, O. T.; Franke, D.; Day, R. A.; Farr, E. P.; Bawendi, M. G.; Sletten, E. M., Flavylium Polymethine Fluorophores for Near- and Shortwave Infrared Imaging. *Angew. Chem. Int. Ed.* **2017,** *56* (42), 13126-13129.

(33) Bandi, V. G.; Luciano, M. P.; Saccomano, M.; Patel, N. L.; Bischof, T. S.; Lingg, J. G. P.; Tsrunchev, P. T.; Nix, M. N.; Ruehle, B.; Sanders, C.; Riffle, L.; Robinson, C. M.; Difilippantonio, S.; Kalen, J. D.; Resch-Genger, U.; Ivanic, J.; Bruns, O. T.; Schnermann, M. J., Targeted multicolor in vivo imaging over 1,000 nm enabled by nonamethine cyanines. *Nat. Methods* **2022,** *19* (3), 353-358.

(34) Chen, H.; Dong, B.; Tang, Y.; Lin, W., A Unique "Integration" Strategy for the Rational Design of Optically Tunable Near-Infrared Fluorophores. *Acc. Chem. Res.* **2017,** *50* (6), 1410-1422.

(35) Yuan, L.; Lin, W.; Chen, H., Analogs of Changsha near-infrared dyes with large Stokes Shifts for bioimaging. *Biomaterials* **2013,** *34* (37), 9566-71.

(36) Zheng, K.; Lin, W.; Huang, W.; Guan, X.; Cheng, D.; Wang, J. Y., Facile synthesis of a class of aminochromene-aniliniumion conjugated far-red to near-infrared fluorescent dyes for bioimaging. *J. Mater. Chem. B* **2015,** *3* (5), 871-877.

(37) Wang, S.; Fan, Y.; Li, D.; Sun, C.; Lei, Z.; Lu, L.; Wang, T.; Zhang, F., Anti-quenching NIR-II molecular fluorophores for in vivo high-contrast imaging and pH sensing. *Nat. Commun.* **2019,** *10* (1), 1058.

(38) Qian, G.; Dai, B.; Luo, M.; Yu, D.; Zhan, J.; Zhang, Z.; Ma, D.; Wang, Z. Y., Band Gap Tunable,

Donor−Acceptor−Donor Charge-Transfer Heteroquinoid-Based Chromophores: Near Infrared Photoluminescence and Electroluminescence. *Chem. Mater.* **2008,** *20* (19), 6208-6216.

(39) Antaris, A. L.; Chen, H.; Cheng, K.; Sun, Y.; Hong, G.; Qu, C.; Diao, S.; Deng, Z.; Hu, X.; Zhang, B.; Zhang, X.; Yaghi, O. K.; Alamparambil, Z. R.; Hong, X.; Cheng, Z.; Dai, H., A small-molecule dye for NIR-II imaging. *Nat. Mater.* **2016,** *15* (2), 235-42.

(40) Kobayashi, N.; Nakajima, S.; Ogata, H.; Fukuda, T., Synthesis, spectroscopy, and electrochemistry of tetra-tert-butylated tetraazaporphyrins, phthalocyanines, naphthalocyanines, and anthracocyanines, together with molecular orbital calculations. *Chem. Eur. J.* **2004,** *10* (24), 6294-312.

(41) Aidas, K.; Angeli, C.; Bak, K. L.; Bakken, V.; Bast, R.; Boman, L.; Christiansen, O.; Cimiraglia, R.; Coriani, S.; Dahle, P.; Dalskov, E. K.; Ekström, U.; Enevoldsen, T.; Eriksen, J. J.; Ettenhuber, P.; Fernández, B.; Ferrighi, L.; Fliegl, H.; Frediani, L.; Hald, K.; Halkier, A.; Hättig, C.; Heiberg, H.; Helgaker, T.; Hennum, A. C.; Hettema, H.; Hjertenæs, E.; Høst, S.; Høyvik, I.-M.; Iozzi, M. F.; Jansík, B.; Jensen, H. J. A.; Jonsson, D.; Jørgensen, P.; Kauczor, J.; Kirpekar, S.; Kjærgaard, T.; Klopper, W.; Knecht, S.; Kobayashi, R.; Koch, H.; Kongsted, J.; Krapp, A.; Kristensen, K.; Ligabue, A.; Lutnæs, O. B.; Melo, J. I.; Mikkelsen, K. V.; Myhre, R. H.; Neiss, C.; Nielsen, C. B.; Norman, P.; Olsen, J.; Olsen, J. M. H.; Osted, A.; Packer, M. J.; Pawlowski, F.; Pedersen, T. B.; Provasi, P. F.; Reine, S.; Rinkevicius, Z.; Ruden, T. A.; Ruud, K.; Rybkin, V. V.; Sałek, P.; Samson, C. C. M.; de Merás, A. S.; Saue, T.; Sauer, S. P. A.; Schimmelpfennig, B.; Sneskov, K.; Steindal, A. H.; Sylvester-Hvid, K. O.; Taylor, P. R.; Teale, A. M.; Tellgren, E. I.; Tew, D. P.; Thorvaldsen, A. J.; Thøgersen, L.; Vahtras, O.; Watson, M. A.; Wilson, D. J. D.; Ziolkowski, M.; Ågren, H., The Dalton quantum chemistry program system. *WIREs Comput. Mol. Sci.* **2014,** 4 (3), 269-284.

(42) Dalton, a molecular electronic structure program, Release v2022.0 , see http://daltonprogram.org.

(43) Lu, T.; Chen, F., Multiwfn: a multifunctional wavefunction analyzer. *J. Comput. Chem.* **2012,** *33* (5), 580-92.

(44) Morozumi, A.; Kamiya, M.; Uno, S.-n.; Umezawa, K.; Kojima, R.; Yoshihara, T.; Tobita, S.; Urano, Y., Spontaneously Blinking Fluorophores Based on Nucleophilic Addition/Dissociation of Intracellular Glutathione for Live-Cell Super-resolution Imaging. *J. Am. Chem. Soc.* **2020,** *142* (21), 9625-9633.

(45) Bultinck, P.; Rafat, M.; Ponec, R.; Van Gheluwe, B.; Carbó-Dorca, R.; Popelier, P., Electron Delocalization and Aromaticity in Linear Polyacenes: Atoms in Molecules Multicenter Delocalization Index. *J. Phys. Chem. A* **2006,** *110* (24), 7642-7648.

(46) Pedersen, J.; Mikkelsen, K. V., A benchmark study of aromaticity indexes for benzene, pyridine and the diazines - I. Ground state aromaticity. *RSC Adv.* **2022,** *12* (5), 2830-2842.

(47) Santos, J. C.; Tiznado, W.; Contreras, R.; Fuentealba, P., Sigma-pi separation of the electron localization function

and aromaticity. *J. Chem. Phys.* **2004,** *120* (4), 1670-3.

(48) Poater, J.; Duran, M.; Solà, M.; Silvi, B., Theoretical Evaluation of Electron Delocalization in Aromatic Molecules by Means of Atoms in Molecules (AIM) and Electron Localization Function (ELF) Topological Approaches. *Chem. Rev.* **2005,** *105* (10), 3911-3947.

(49) Villaume, S.; Fogarty, H. A.; Ottosson, H., Triplet-state aromaticity of 4npi-electron monocycles: analysis of bifurcation in the pi contribution to the electron localization function. *ChemPhysChem* **2008,** *9* (2), 257-64.
