# Supplementary material for: Ultra-photostable small-molecule dyes facilitate near-infrared biophotonics
Source: Nat Commun. 2024 Mar 22;15:2593. doi: 10.1038/s41467-024-46853-0 (PMC10960032; doi:10.1038/s41467-024-46853-0)
Supplement: Supplementary file 3 — Description of Additional Supplementary Files [file 41467_2024_46853_MOESM3_ESM.pdf]

**Description of Additional Supplementary Files**

**File Name: Supplementary Data 1**
**Description: Atomic coordinates.** Atomic coordinates applied in quantum chemical calculation.

**File Name: Supplementary Movie 1**
**Description: Photostability of AF3 in live-cell imaging.** The microscopic imaging (upper right) and fluorescence intensity statistics (lower right) of each frame under 808 nm excitation after AF3 staining. Left panel shows the fluorescence intensity of the first frame along selected area (yellow dashed line) in the image.

**File Name: Supplementary Movie 2**
**Description: Photostability of Cy7 in live-cell imaging.** The microscopic imaging (upper right) and fluorescence intensity statistics (lower right) of each frame under 808 nm excitation after Cy7 staining. Left panel shows the fluorescence intensity of the first frame along selected area (yellow dashed line) in the image.
